# Global burden of traumatic brain injury from 1990 to 2021 and projections to 2050: A GBD 2021–based study using interpretable machine learning

**DOI:** 10.1097/MD.0000000000049918

**Published:** 2026-07-24

**Authors:** Shiying Dong, Ming Qiu, Mintao Li, Fangbing Li, Di Wu, Meng Nie, Jiangyuan Yuan, Chenrui Wu, Fei Song, Chuang Gao, Rongcai Jiang

**Affiliations:** a Department of Neurosurgery, Tianjin Neurological Institute, State Key Laboratory of Experimental Hematology, Laboratory of Post-Neuroinjury Neurorepair and Regeneration in Central Nervous System, Tianjin and Ministry of Education, Tianjin Medical University General Hospital, Tianjin, China; b Department of Vascular Surgery, Shanghai Traditional Chinese Medicine Integrated Hospital, Affiliated to University of Shanghai Traditional Chinese Medicine, Shanghai, China; c Department of Infectious Diseases, Tianjin Medical University General Hospital, Tianjin, China; d Department of Neurosurgery, Second Affiliated Hospital, School of Medicine, Zhejiang University, Hangzhou, Zhejiang, China; e Department of Neurosurgery, Sichuan Provincial People’s Hospital, University of Electronic Science and Technology of China, Chengdu, China; f Department of Neurosurgery, Jiayuguan Hospital of Traditional Chinese Medicine, Gansu, China; g Department of Neurosurgery, Xuanwu Hospital, Capital Medical University, Beijing, China.

**Keywords:** Global Burden of Disease, machine learning, risk factors, SHAP, sociodemographic index, traumatic brain injury, XGBoost

## Abstract

Traumatic brain injury (TBI) is a major cause of disability and socioeconomic burden worldwide. Using updated Global Burden of Disease Study 2021 estimates, this study assessed global TBI burden from 1990 to 2021 and projected future patterns to 2050. Global Burden of Disease Study 2021 estimates were used to evaluate TBI incidence, prevalence, and years lived with disability (YLDs) in 204 countries and territories. Age-standardized rates were stratified by age, sex, region, and sociodemographic index (SDI). Age-period-cohort (APC) modeling, frontier analysis, and inequality metrics were applied to characterize temporal patterns and disparities. Bayesian APC models projected population-level burden to 2050, and eXtreme Gradient Boosting-SHapley Additive exPlanation models were used as supplementary tools for prediction and interpretation. Model performance was assessed using cross-validation. From 1990 to 2021, incident, prevalent, and YLD cases increased by 22.35%, 53.27%, and 52.65%, respectively, whereas the corresponding age-standardized rates declined by 20.16%, 16.52%, and 16.19%. Men consistently had a higher burden than women. The APC model suggested a transition around 52.5 years, indicating a shift toward older populations. In low-SDI regions, the age-standardized YLD rate changed by 1.14% (95% uncertainty interval: −2.32 to 5.03), indicating no statistically significant change. SDI showed an inverted U-shaped association with YLD rates. Age-standardized incidence rate increased in the Caribbean and Oceania. Cross-validation showed close agreement between observed and predicted values (cross-validated *R*^2^ = 0.997–0.998; mean absolute error = 2.27–16.64). Projections suggested declining age-standardized rates through 2050. Global TBI burden increased in absolute numbers but declined in age-standardized rates. Persistent age, sex, socioeconomic, and regional disparities support targeted prevention, trauma-care strengthening, and rehabilitation planning.

## 1. Introduction

Traumatic brain injury (TBI) is a major cause of death, disability, and long-term neurological impairment worldwide. As both an acute injury and a potential chronic health condition, TBI imposes substantial clinical, social, and economic burdens on patients, families, healthcare systems, and society.^[1–5]^ Survivors may experience persistent cognitive, behavioral, physical, and psychosocial sequelae, which increase the need for long-term care and rehabilitation. Because many TBI events are preventable, monitoring the changing epidemiological burden of TBI is essential for guiding prevention strategies, trauma-care planning, and health-resource allocation.

Previous Global Burden of Disease (GBD)–based studies have substantially improved understanding of the epidemiology of TBI. Earlier analyses described the global, regional, and national burden of neurological disorders and TBI-related outcomes, while more recent studies have evaluated mild TBI and the global burden of TBI using GBD 2019 or GBD 2021 data.^[6–8]^ These studies showed that the absolute number of TBI cases has continued to increase in many settings, whereas age-standardized rates have generally declined, suggesting the combined influence of population growth, population aging, injury-prevention efforts, and changes in healthcare systems. However, important questions remain regarding the joint patterns of age, sex, sociodemographic development, injury mechanisms, and regional heterogeneity in shaping the burden of TBI.

The burden of TBI is not evenly distributed across populations. Age is a key determinant of TBI burden, with younger populations often affected by transport-, sports-, or occupation-related injuries, whereas older adults are increasingly affected by fall-related injuries and age-related vulnerability.^[9,10]^ Sex differences are also important, as males generally experience a higher TBI burden than females, although this difference varies across age groups, regions, and injury mechanisms.^[11,12]^ In addition, the relationship between sociodemographic index (SDI) and TBI burden is complex. Countries with different levels of socioeconomic development may differ in injury exposure, traffic and occupational safety, trauma-care access, rehabilitation capacity, surveillance systems, and population aging.^[13]^ Therefore, a multidimensional analysis stratified by age, sex, SDI, region, and injury mechanism may provide more useful evidence for targeted prevention than analyses based only on overall trends.

GBD 2021 provides updated estimates through 2021 and allows a more current assessment of TBI burden than earlier GBD datasets.^[3,14]^ Compared with analyses based on GBD 2019 or older data, GBD 2021 enables evaluation of more recent demographic and epidemiological changes and supports updated projections of future burden. However, updated burden estimates alone are insufficient for policy planning. Methods that can characterize temporal patterns, socioeconomic inequalities, and future trends are also needed. Age-period-cohort (APC) modeling can help separate age-, period-, and cohort-related patterns, while inequality and frontier analyses can quantify differences across levels of socioeconomic development. Bayesian projection can be used to estimate future population-level burden, and interpretable machine learning may further help identify major predictors and nonlinear patterns in complex epidemiological data.

Therefore, the present study used GBD 2021 data to evaluate the global, regional, and national burden of TBI from 1990 to 2021 and to project the burden to 2050. We assessed incidence, prevalence, and years lived with disability (YLDs) across 204 countries and territories, with stratification by age, sex, SDI, and region. We further applied APC modeling, frontier analysis, inequality metrics, Bayesian projection, and eXtreme Gradient Boosting (XGBoost)-SHapley Additive exPlanations (SHAP)–based interpretable machine learning to characterize temporal changes, socioeconomic disparities, regional heterogeneity, and future burden patterns.

Based on previous evidence and the analytical framework of this study, we proposed the following hypotheses: first, the absolute burden of TBI increased from 1990 to 2021, whereas age-standardized rates declined; second, the burden of TBI shifted toward older populations and remained substantial in lower-resource settings; third, sex-specific differences persisted but varied across age groups, regions, and injury mechanisms; and fourth, interpretable machine-learning models could identify important population-level predictors and support projection of future TBI burden. The findings may provide updated evidence for targeted prevention, trauma-care improvement, rehabilitation planning, and resource allocation.

## 2. Methods

### 2.1. Data sources, study design, and case definition

This study followed the Guidelines for Accurate and Transparent Health Estimates Reporting and the Strengthening the Reporting of Observational Studies in Epidemiology guidelines.^[15,16]^ We conducted a global ecological secondary analysis using publicly available estimates from the Global Burden of Disease Study 2021 (GBD 2021) to assess the burden of TBI from 1990 to 2021 and to project its future burden to 2050. The analysis included 204 countries and territories and 811 subnational locations. All available age groups in the GBD framework were included, and age-group midpoints ranging from 2.5 to 97.5 years were used for age-stratified analyses and machine-learning modeling where applicable.

TBI was defined according to the GBD 2021 injury framework as injury to the head resulting in short-term or long-term neurological dysfunction of the brain.^[14,17]^ The case definition was based on corresponding International Classification of Diseases (ICD), Ninth Revision and Tenth Revision codes and GBD cause-of-injury and nature-of-injury mapping. TBI cases were further categorized using GBD nature-of-injury categories, including ICD-N-27 and ICD-N-28, and severity was classified as mild or moderate/severe according to the GBD 2021 framework. The main burden indicators were incident cases, prevalent cases, and YLDs, together with age-standardized incidence rates (ASIRs), age-standardized prevalence rates (ASPRs), and age-standardized YLD rates. All rates were expressed per 100,000 population.

The GBD estimates used in this study were final modeled estimates rather than primary observed individual-level, hospital-level, or registry-level records. We used GBD-provided point estimates and 95% uncertainty intervals (UIs). Missingness and data sparsity were addressed within the GBD estimation framework, and no additional individual-level imputation was performed by the authors. Because coding practices, diagnostic capacity, injury surveillance, and access to medical care may differ across locations and over time, cross-country and temporal comparisons were interpreted as population-level modeled patterns rather than direct individual-level epidemiological observations.

### 2.2. Analytical framework

The analyses were organized sequentially to provide a multidimensional assessment of TBI burden. First, descriptive analyses characterized global, regional, national, age-, sex-, and SDI-specific patterns in incidence, prevalence, and YLDs from 1990 to 2021. Second, APC modeling was used to evaluate age-, period-, and cohort-related temporal patterns. Third, inequality and frontier analyses were used to assess whether TBI burden differed across levels of socioeconomic development and to identify locations with higher-than-expected burden relative to their SDI. Fourth, severity-, sex-, and mechanism-stratified analyses were conducted to describe differences in TBI burden by injury severity and cause pattern. Fifth, Bayesian age-period-cohort (BAPC) models were used to project future burden to 2050. Finally, XGBoost-SHAP modeling was applied as a supplementary exploratory approach to summarize nonlinear demographic and temporal patterns in GBD-derived aggregate estimates. These analyses were intended to complement each other and were not designed to provide independent causal inference from each method.

### 2.3. Age standardization and SDI classification

Age-standardized rates were calculated using direct standardization. For each age group, the age-specific rate was multiplied by the corresponding age weight from the World Health Organization 2000 to 2025 standard population, and the weighted sum was used to obtain the age-standardized rate per 100,000 population.^[18]^ The same approach was applied to ASIR, ASPR, and age-standardized YLD rates.

Socioeconomic development was measured using the SDI, a composite indicator developed within the GBD framework. SDI ranges from 0 to 1 and is based on lag-distributed income per capita, mean educational attainment among individuals aged 15 years or older, and total fertility rate among individuals younger than 25 years. Countries and territories were classified into five SDI categories according to GBD classifications: low, low-middle, middle, high-middle, and high SDI.^[14]^ Temporal changes between 1990 and 2021 were calculated as percentage changes using the formula ([value in 2021 − value in 1990] / value in 1990) × 100%.

### 2.4. APC modeling

APC modeling was used to decompose temporal variation in TBI burden into age, period, and birth-cohort components.^[19]^ Age was grouped according to available GBD age intervals, calendar period was defined using GBD observation years from 1990 to 2021, and birth cohort was derived from age and period. Separate APC models were constructed for incidence, prevalence, and YLD rates. The model incorporated age effects, period effects, cohort effects, and age–period interaction terms where applicable. Model parameters were estimated using constrained generalized linear modeling to address the non-identifiability problem inherent to APC analysis. Net drift, local drift, age effects, period effects, and cohort effects were used to summarize overall temporal trends, age-specific annual changes, and relative risks across periods and birth cohorts. APC results were interpreted descriptively as population-level temporal patterns.

### 2.5. Cross-country inequality analysis

Cross-country inequalities in TBI burden were evaluated using the slope index of inequality (SII) and concentration index (ConcI).^[20]^ Countries and territories were ranked according to SDI, and population weights were incorporated to account for differences in population size. The SII quantified absolute inequality and represented the modeled difference in TBI burden between the lowest and highest ends of the SDI distribution. A larger absolute SII indicated a greater absolute gap across socioeconomic development levels. The ConcI quantified relative inequality and measured whether TBI burden was disproportionately concentrated among lower- or higher-SDI populations. Negative ConcI values indicated greater concentration of burden among lower-SDI populations, whereas positive values indicated greater concentration among higher-SDI populations. Confidence intervals were estimated using bootstrap resampling or the delta method, where applicable. To assess temporal changes in inequality, differences in SII between 1990 and 2021 were calculated. Standard errors (SEs) were derived from the reported 95% confidence intervals (CIs) using the formula: SE = (upper CI − lower CI) / 3.92. The SE of the difference was calculated assuming independence between estimates. Statistical significance was evaluated using a two-sided *Z*-test, with *P* <.05 considered statistically significant. Because SII represents aggregated measures rather than paired observations, paired *t* tests or Wilcoxon tests were not applied.

### 2.6. Frontier analysis

Frontier analysis was used to benchmark the observed TBI burden of each country or territory against the lowest burden achieved at comparable levels of SDI. For each indicator, the association between age-standardized TBI burden and SDI was modeled, and a frontier curve was fitted to represent the theoretical minimum or best-achieved burden at a given SDI level. The distance-to-frontier was defined as the vertical difference between the observed age-standardized rate and the frontier-predicted value at the same SDI. Larger distance-to-frontier values indicated higher-than-expected burden relative to countries with similar socioeconomic development. Frontier results were interpreted as descriptive benchmarks for identifying potentially preventable excess burden rather than as causal estimates or fixed policy targets.

### 2.7. Risk-factor, severity, and sex-stratified analysis

Because the present study focused on GBD-estimated TBI incidence, prevalence, and YLDs, injury patterns were summarized descriptively rather than through independent population-attributable fraction analysis. TBI burden was compared across sex, age group, SDI category, GBD region, country or territory, severity category, and injury mechanism. Mild and moderate/severe TBI were analyzed separately where data were available. The distribution of TBI by injury mechanism was assessed to identify leading contributors to TBI burden in 2021, including falls and exposure to mechanical forces. Heat maps and descriptive comparisons were used to visualize differences in severity-specific and mechanism-specific burden across sex, SDI group, region, and country. Subgroup comparisons were interpreted as descriptive patterns based on modeled GBD estimates.

### 2.8. Burden projection

Future TBI burden from 2022 to 2050 was projected using BAPC models combined with United Nations population projections.^[21,22]^ Separate projection models were constructed for incidence, prevalence, and YLDs, and projections were further stratified by sex where applicable. Observed cases were assumed to follow a Poisson distribution, with the expected number of cases determined by population size and estimated age-specific rates. Random-walk priors were assigned to period and cohort effects to allow gradual temporal changes over time. Posterior distributions were generated using 1000 Markov chain Monte Carlo simulations. The 95% UI was defined as the 2.5th and 97.5th percentiles of the posterior distribution. Projection results were interpreted as conditional forecasts based on historical APC patterns and projected population structure, rather than as deterministic predictions. Potential future changes in trauma systems, road-safety policies, occupational exposure, conflict, healthcare access, coding practices, and rehabilitation capacity were not directly modeled.

### 2.9. XGBoost-SHAP modeling and web tool

As a supplementary exploratory complement to the epidemiological and projection analyses, rather than an independent predictor-discovery framework, we developed XGBoost models to summarize nonlinear patterns in GBD-derived aggregate estimates of TBI incidence, prevalence, and YLDs.^[23]^ Separate models were constructed for incidence, prevalence, and YLDs using age, sex, calendar year, and log-transformed population size as input variables. These variables were selected because they were consistently available across countries, years, age groups, and sexes within the GBD framework. Therefore, the XGBoost component was intended to provide an interpretable summary of nonlinear model-learned patterns in population-level burden estimates, rather than to identify novel etiological predictors or replace the APC/BAPC-based epidemiological analyses.

Model hyperparameters were tuned using Bayesian optimization where applicable. Model performance was internally evaluated using cross-validation. Cross-validated *R*^2^ and mean absolute error (MAE) were calculated to quantify the agreement between GBD-estimated and model-predicted values for incidence, prevalence, and YLDs. These metrics were interpreted as internal validation results within the GBD-derived aggregate-data framework rather than as external validation using independent primary datasets.

Model interpretability was assessed using SHAP.^[24,25]^ SHAP beeswarm plots were used to summarize the distribution, direction, and magnitude of feature contributions. Feature-importance plots ranked predictors according to mean absolute SHAP values, and SHAP dependence plots were used to explore nonlinear associations between individual predictors and predicted TBI burden. SHAP results were interpreted as model-based explanations of aggregate GBD-derived predictions, rather than as causal effects or independent epidemiological evidence.

An interactive Streamlit web tool was developed to provide a research-use interface for exploring population-level TBI burden predictions and SHAP visualizations. The tool allows users to input demographic and temporal variables and view model-predicted burden estimates with corresponding interpretability outputs. The web tool is intended only for research, education, and public-health exploration. It should not be used for individual clinical decision-making, diagnosis, treatment selection, prognosis estimation, or patient-level risk stratification.^[26,27]^

### 2.10. Statistical software, uncertainty, and inference scope

All statistical analyses and visualizations were performed using R software version 4.4.2, GraphPad Prism version 9.3.1, and Python software version 3.10.9. A two-sided *P* value <.05 was considered statistically significant where hypothesis testing was performed. All GBD estimates were reported with 95% UIs. For percentage changes, uncertainty was evaluated using GBD-provided uncertainty estimates, and changes were interpreted cautiously when the 95% UI crossed zero. Although UIs were incorporated into descriptive analyses and BAPC projections where available, uncertainty from GBD input estimates was not fully propagated through all secondary APC, frontier, inequality, and machine-learning analyses. Because this study used aggregate modeled estimates rather than individual-level data, all findings should be interpreted as population-level associations and modeled patterns, not as causal effects or direct individual-level observations.

### 2.11. Ethical considerations

This study used publicly available, aggregated, de-identified GBD estimates and did not include individual-level patient information. Therefore, institutional review board approval and informed consent were not required.

## 3. Results

### 3.1. Global trends

The absolute number of incident TBI cases increased by 22.35% from 1990 to 2021, from 17.0 million to 20.8 million cases. In contrast, the ASIR decreased by 20.16%. The absolute number of prevalent cases increased by 53.27%, from 24.74 million to 37.92 million cases, while the age-standardized prevalence rate decreased by 16.52%. The absolute number of YLDs increased by 52.65%, from 3.59 million to 5.48 million, whereas the age-standardized YLD rate decreased by 16.19% (Tables S1–S4, Supplemental Digital Content 1).

### 3.2. Global trends by sex

The sex ratio (male/female) was generally stable between 2.04 and 2.08, and the incidence of males was consistently higher than that of females, with significant regional differences (e.g., 2.91 to 3.22 in Central Asia), highlighting the persistent influence of sex-specific exposure risk. The male-to-female prevalence ratio remained stable at 2.15 to 2.17 globally, but the sex ratio increased in regions such as Central Africa (for example, from 1.84 to 2.22 in Central and Western sub-Saharan Africa), which may be related to occupational injuries or gender inequality in medical access. Globally, the burden of YLDs in men was always higher than that in women (the male-to-female ratio increased from 2.21 to 2.24), and the male-to-female ratio increased significantly in Central and Western sub-Saharan Africa (the male-to-female ratio increased from 1.87 to 2.27; Tables S1–S4, Supplemental Digital Content 1).

**Figure 1.**
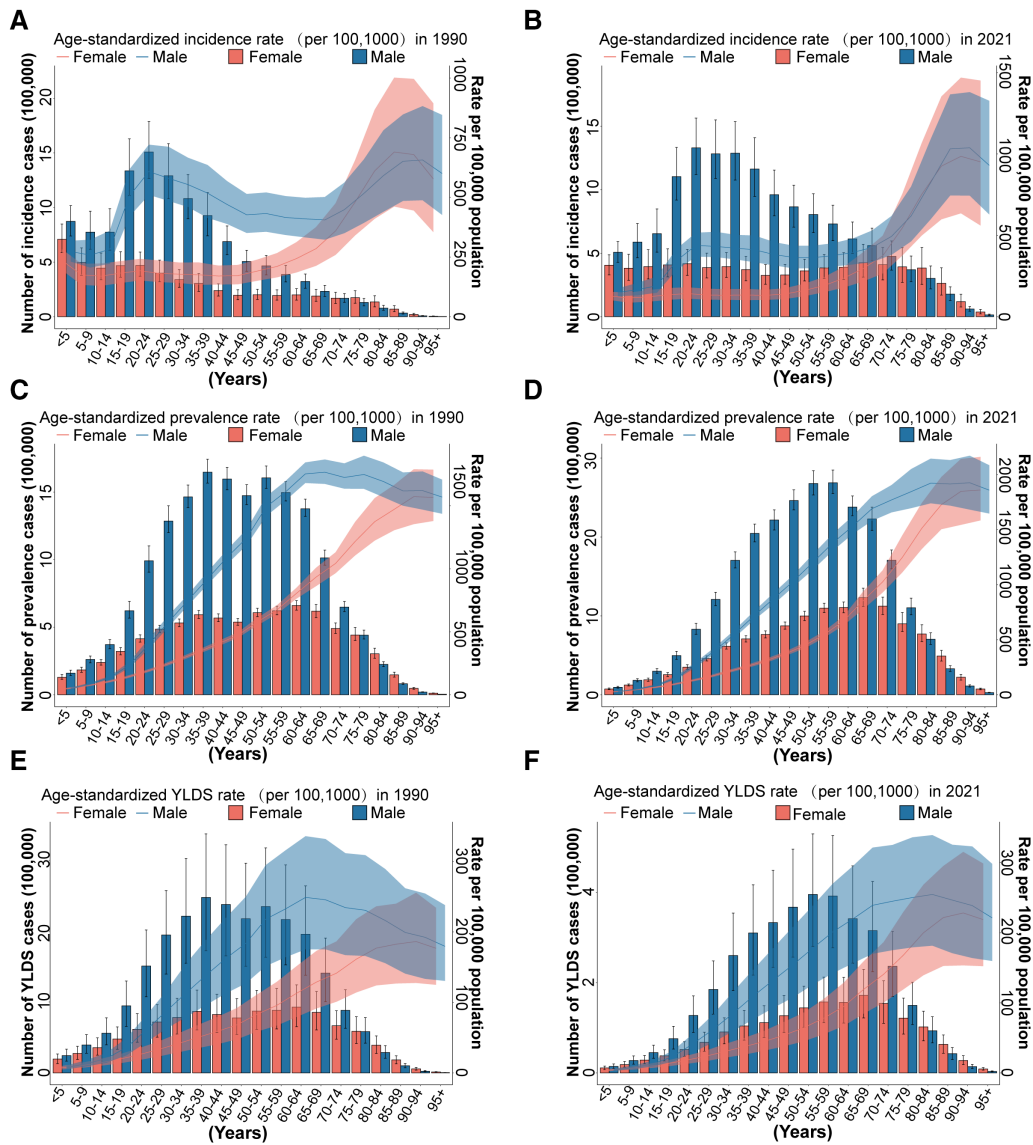
Temporal evolution of sex- and age-specific, age-standardized incidence, prevalence, and YLD rates from 1990 to 2021. (A) Age-standardized incidence rates by sex and age in 1990; (B) age-standardized incidence rates by sex and age in 2021; (C) age-standardized prevalence rates by sex and age in 1990; (D) age-standardized prevalence rates by sex and age in 2021; (E) age-standardized YLD rates by sex and age in 1990; (F) age-standardized YLD rates by sex and age in 2021. YLDs = years lived with disability.

According to the sex-age distribution of TBI incidence, prevalence, and YLDs, the temporal evolution of the burden of TBI was further analyzed. As shown in Figure 1A and B, in 1990, the age group with a high incidence of males was concentrated between 40 and 60 years old (nearly 20/100,000), while there was no obvious concentration trend in females, and the peak amplitude was gentle (about 10/100,000). In 2021, the peak amplitude of male incidence dropped to below 15/100,000, and the age of onset was still in the young and middle-aged stage. The age fluctuation of male and female incidence rates tended to be the same. As for the analysis of the prevalence burden of TBI, as shown in Figure 1C and D, the prevalence of TBI in both men and women showed an obvious upward trend in 1990 and 2021, with a peak in men at the age of 60 to 70, with a prevalence of more than 20/100,000, and a lagging peak and a lower amplitude in women. In 1990, the peak of YLDs of TBI in males was prominent at the age of 45 to 55 years (>20/100,000), while the peak in females was lower and delayed (<15/100,000). The magnitude of YLDs decreased in both sexes in 2021 (to less than 15 per 100,000 for men and nearly 10 per 100,000 for women), reflecting a narrowing gender gap (Fig. 1E and F).

In addition, descriptive correlation analysis was conducted among different TBI levels, gender differences, and different SDI differences (see Fig. S3, Supplemental Digital Content 2). In 2021, the most common cause of TBI in all degrees was falls worldwide, and there were gender and regional differences. In mild TBI, the most common cause of injury in Australasia was exposure to mechanical forces, which was higher in males than in females. The most common cause of moderate/severe TBI in South Asia was falls, which was higher in females than in males.

### 3.3. Global trends by age group

**Figure 2.**
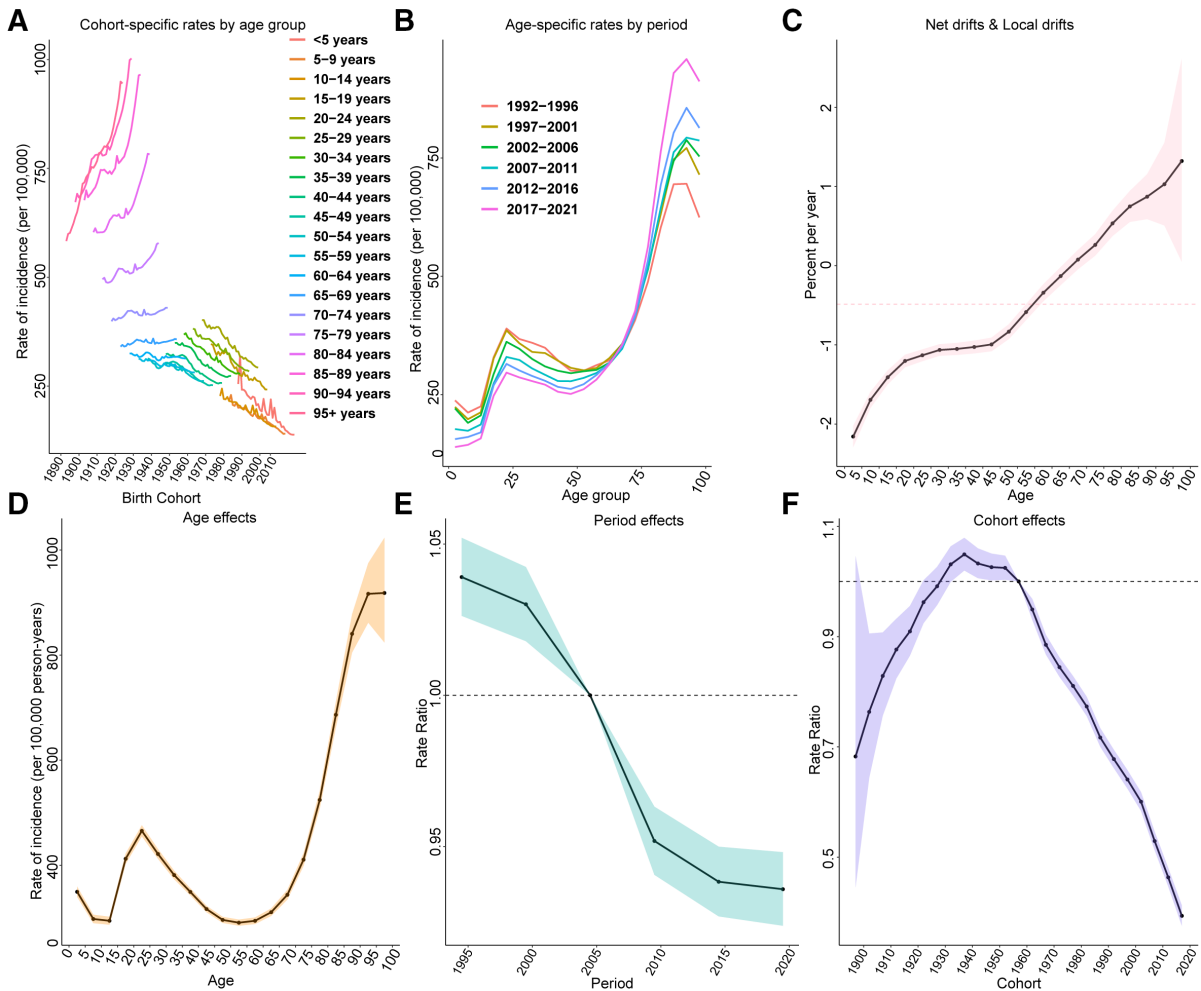
APC analysis of the burden of TBI incidence. (A) Cohort-specific incidence trends by age group; (B) age-specific incidence changes by period; (C) annual rate of change in incidence by age using net and local drift analyses; (D) nonlinear age effect on incidence; (E) period effect showing changes in relative risk over time; (F) cohort effect showing differences across birth cohorts. APC = age-period-cohort, TBI = traumatic brain injury.

According to the APC model, the incidence of TBI increased from 50 to 75 years of age, was stable from 25 to 50 years of age, and decreased from 15 years of age (Fig. 2A and B). According to the Local Drifts chart, with 52.5 years old (net drift: −0.59, −0.49 to −0.69) as the boundary, the incidence showed a downward trend in the younger group and an upward trend in the older group (Fig. 2C). In addition, according to the results in Figure 2D, the incidence of TBI increased sharply from 294.3 to 465.5 and from 343.8 to 918.3 (per 100,000 population) at ages 12.5 to 22.5 years and 67.5 to 97.5 years, respectively. The relative risk of incidence for TBI was high until 2004.5 and declined significantly thereafter (Fig. 2E). In addition, there was a gradual increase in incidence among persons born before 1937 (odds ratio: 1.05, 1.02–1.08) and a subsequent decline (Fig. 2F).

Supplementary APC analyses showed broadly similar age-related patterns for prevalence and YLD rates. Prevalence and YLD rates generally increased with age, with a higher burden among older age groups. Cohort analyses suggested higher relative risks among earlier birth cohorts, followed by a decline in later cohorts (Figs. S1 and S2, Supplemental Digital Content 3).

### 3.4. Global trends by SDI

For TBI incidence, age-standardized rates decreased in all SDI groups, with the largest reduction in the high-SDI group (−23.48%, 95% UI: −26.29 to −20.73) and the smallest reduction in the low-SDI group (−14.71%, 95% UI: −18.54 to −11.06). For prevalence, the high-SDI group (−21.08%) and high-middle-SDI group (−22.64%) showed the largest declines, while the low-SDI group showed no statistically significant change in ASPR (1.13%, 95% UI: −2.15 to 4.46). For YLDs, the most pronounced decreases were observed in the high-SDI group (−20.61%), high-middle-SDI group (−22.46%), and middle-SDI group (−10.45%). In the low-SDI group, the age-standardized YLD rate changed by 1.14% (95% UI: −2.32 to 5.03), indicating no statistically significant change (Tables S1–S4, Supplemental Digital Content 1).

**Figure 3.**
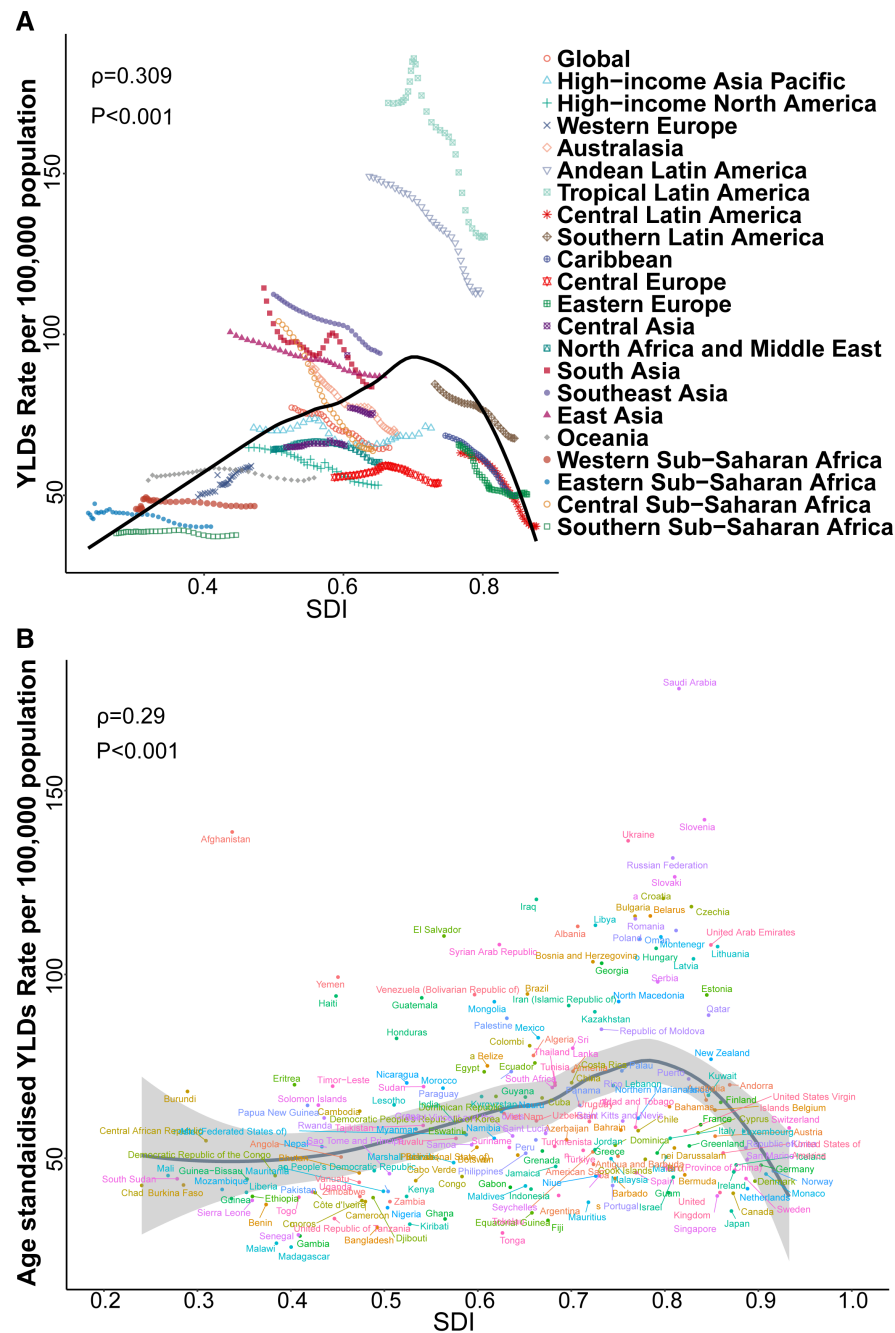
Association and heterogeneity between global age-standardized YLD rates and the SDI. (A) Regional-level association between age-standardized YLD rate and SDI; (B) country-level association between age-standardized YLD rate and SDI. SDI = sociodemographic index, YLDs = years lived with disability.

This study further examined the association between YLD rate and SDI and the development of YLDs. Both regional subgroups (Fig. 3A, ρ = 0.309, *P* < .001) and country subgroups (Fig. 3B, ρ = 0.29, *P* < .001) showed an “inverted U-shaped” nonlinear relationship between YLDs rate and SDI. At lower stages of SDI (SDI < 0.701), YLDs increased as SDI increased. After reaching a peak of 185.8 per 100,000 people, it started to decline. In terms of country and region heterogeneity, low-SDI regions such as sub-Saharan Africa had a higher peak and persistent YLDs rate, while high-income regions such as North America and Western Europe had a decline in high SDI burden.

**Figure 4.**
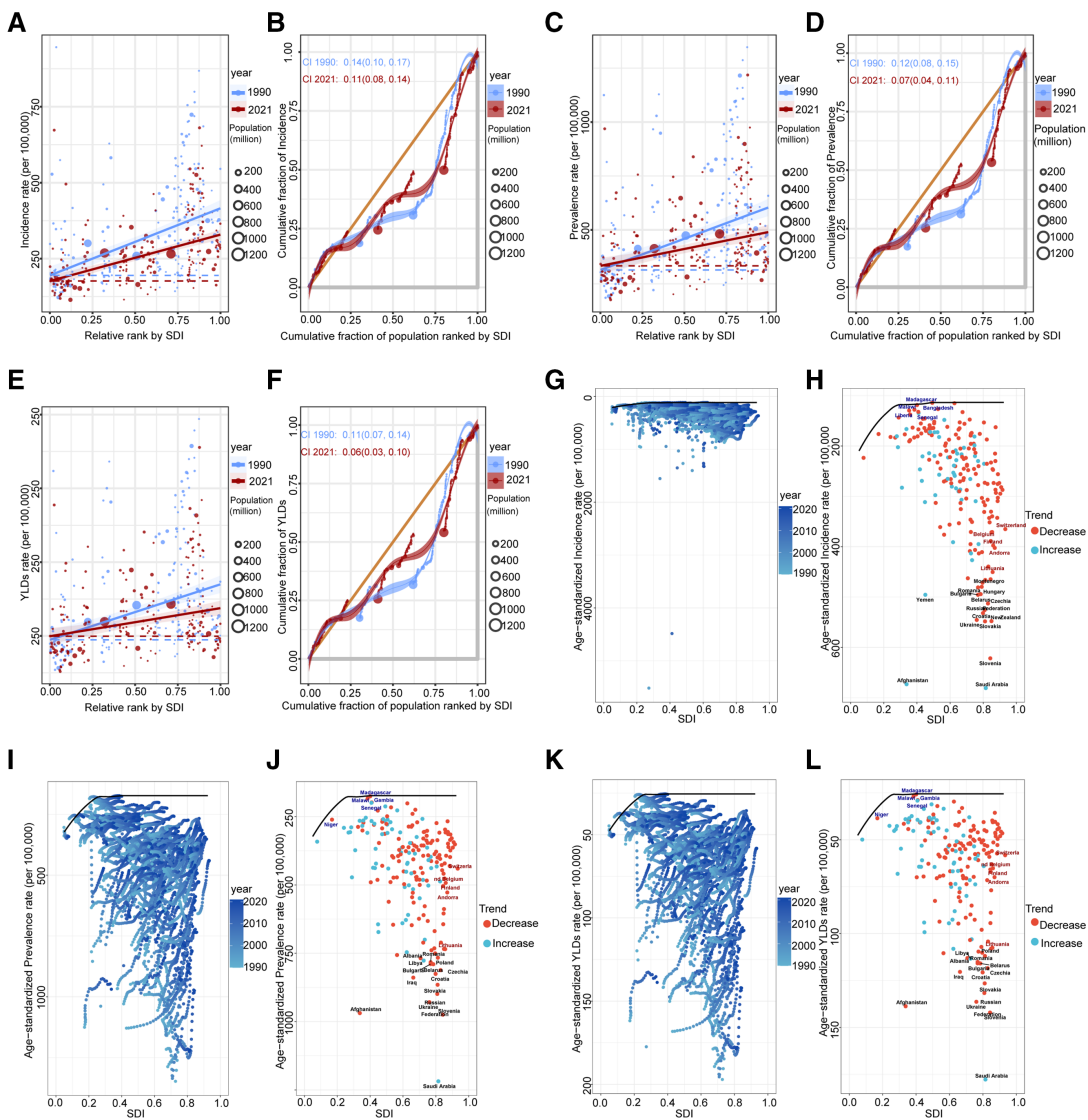
Spatiotemporal distribution and evolution of TBI health indicators according to SDI. (A–F) Absolute and relative inequality analyses for incidence, prevalence, and YLD burden; (G–L) spatiotemporal associations between age-standardized rates and SDI for incidence, prevalence, and YLDs. SDI = sociodemographic index, TBI = traumatic brain injury, YLDs = years lived with disability.

The study also conducted a health inequality analysis of each epidemiological factor of TBI, as shown in Figure 4A to F. Health inequality analysis based on the slope index of inequality (SII) showed statistically significant reductions in absolute disparities in TBI burden between 1990 and 2021 (Table 1). The SII for ASIR decreased from 220.94 (95% CI: 167.65–274.24) to 152.81 (117.92–187.70), with a difference of −68.13 (95% CI: −110.5 to −25.8; *P* = .002). The SII for ASPR declined from 289.41 (212.22–366.61) to 156.69 (94.85–218.52), corresponding to a reduction of −132.72 (−194.6 to −70.8; *P* < .001). The SII for YLDs rate decreased from 37.61 (26.34–48.88) to 19.06 (9.98–28.13), with a difference of −18.55 (−28.6 to −8.5; *P* < .001). But clear disparities still exist, with regions with higher levels of socioeconomic development generally having lower TBI burden. Higher burdens persisted in several lower-SDI settings. In addition, as shown in Figure 4G to L, the incidence of TBI was relatively stable with the increase of SDI, while the prevalence and YLDs showed a decreasing trend, but there were also differences. For example, the incidence of TBI increased while the prevalence and YLDs decreased in Afghanistan with low SDI (SDI < 0.3). Regions with high SDI (SDI > 0.8), such as Slovenia, showed a decrease in incidence, whereas Saudi Arabia showed an increase in incidence, prevalence, and YLDs.

**Table 1:** Changes in gross disparities of ASIR, ASPR, and YLD rate for TBI for all ages between the highest- and lowest-SDI countries, 1990–2021.

Indicator	1990 SII (95% CI)	2021 SII (95% CI)	Difference (95% CI)	*P* value
ASIR	220.94 (167.65 to 274.24)	152.81 (117.92 to 187.70)	−68.13 (−110.5 to −25.8)	.002
ASPR	289.41 (212.22 to 366.61)	156.69 (94.85 to 218.52)	−132.72 (−194.6 to −70.8)	<.001
YLDs rate	37.61 (26.34 to 48.88)	19.06 (9.98 to 28.13)	−18.55 (−28.6 to −8.5)	<.001

ASIR = age-standardized incidence rate, ASPR = age-standardized prevalence rate, CI = confidence interval, SDI = sociodemographic index, SII = slope index of inequality, TBI = traumatic brain injury, YLDs = years lived with disability.

### 3.5. Global trends by region and country

**Figure 5.**
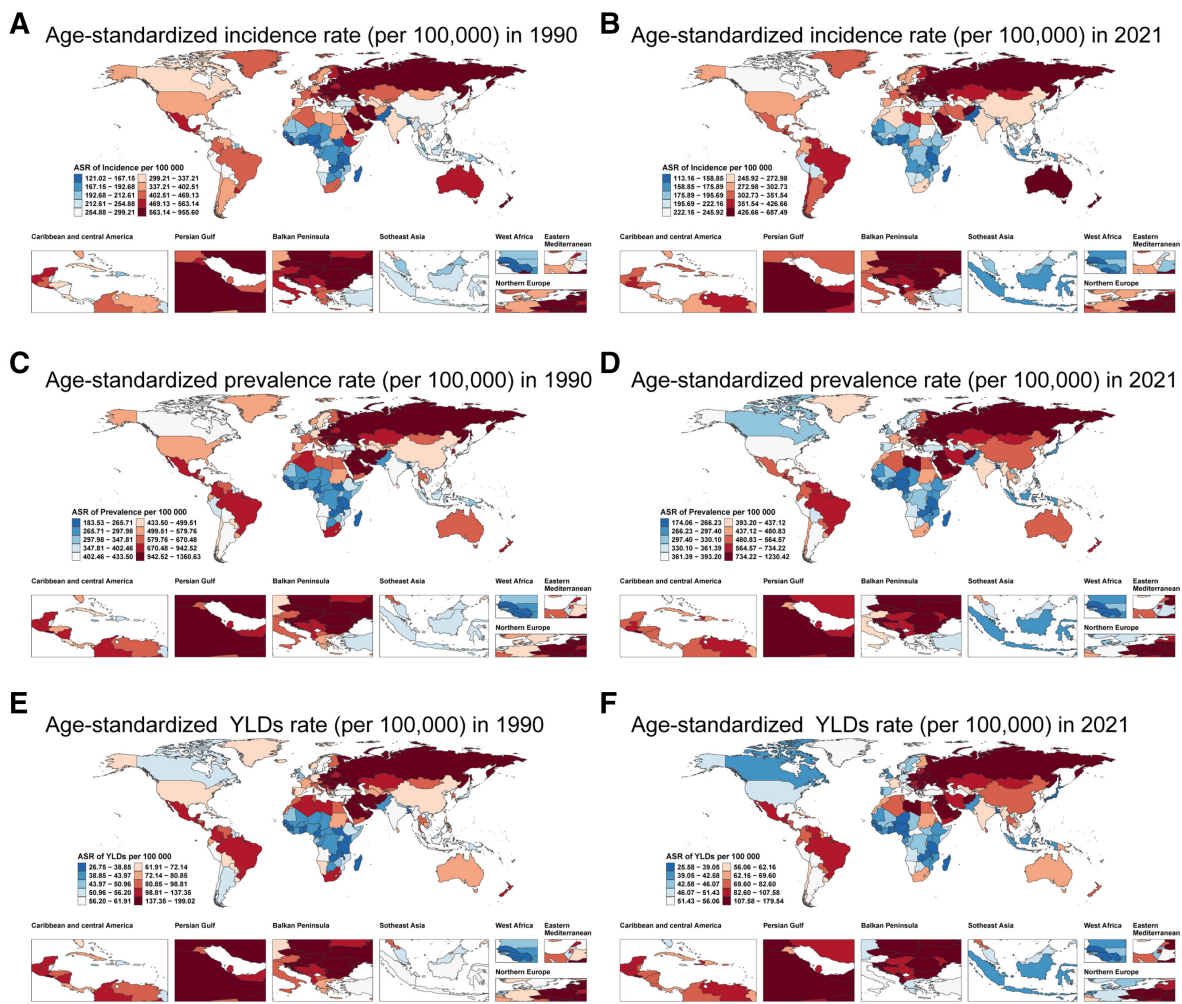
Global and regional incidence, prevalence, and YLD burden of TBI from 1990 to 2021. (A) age‐standardized incidence rate per 100,000 population in 1990; (B) age‐standardized incidence rate per 100,000 population in 2021; (C) age‐standardized prevalence rate per 100,000 population in 1990; (D) age‐standardized prevalence rate per 100,000 population in 2021; (E) age‐standardized years lived with disability (YLD) rate per 100,000 population in 1990; and (F) age‐standardized YLD rate per 100,000 population in 2021. TBI = traumatic brain injury, YLDs = years lived with disability.

Regional analysis showed that ASIR decreased in most regions, including Eastern Europe (−25.19%) and Western Europe (−25.92%). However, ASIR increased by 14.34% (95% UI: 9.59–20.65) in the Caribbean and by 15.55% (95% UI: 11.65–19.05) in Oceania. Regions with notable reductions in TBI prevalence included high-income Asia Pacific (−36.13%), Western Europe (−27.11%), and Southern sub-Saharan Africa (−38.20%). In contrast, age-standardized prevalence increased in the Caribbean (17.11%, 95% UI: 9.64–29.81) and Oceania (19.85%, 95% UI: 17.15–23.26). For YLDs, improvements were observed in high-income Asia Pacific (−36.09%), Southern sub-Saharan Africa (−38.72%), and Western Europe (−27.22%), whereas increases were observed in the Caribbean (16.28%, 95% UI: 8.47–29.79) and Oceania (19.55%, 95% UI: 14.73–25.80). These regional differences may reflect variations in injury patterns, trauma-care capacity, rehabilitation access, and surveillance systems. The age-standardized rate of YLDs in East Asia remained almost unchanged (−0.05%, 95% UI: −2.01 to 2.12), suggesting stagnation in age-standardized burden (Fig. 5 and Tables S1–S4, Supplemental Digital Content 1).

### 3.6. Machine-learning prediction and SHAP interpretation

**Figure 6.**
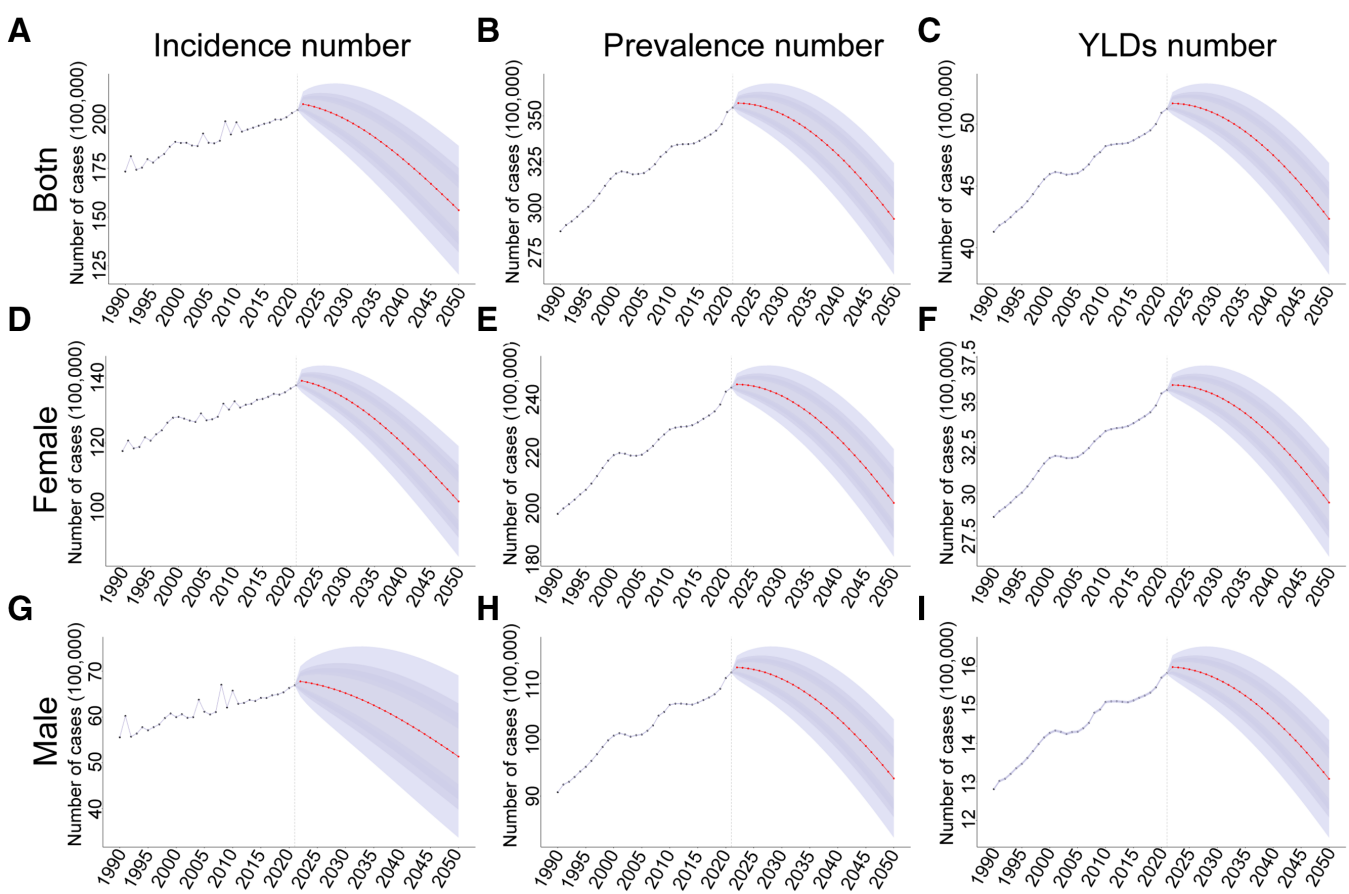
Historical burden of TBI from 1990 to 2021 and projected burden from 2022 to 2050. (A) Overall incidence, (B) overall prevalence, (C) overall YLDs, (D) female incidence, (E) female prevalence, (F) female YLDs, (G) male incidence, (H) male prevalence, (I) male YLDs. Projected lines represent central estimates; corresponding 95% uncertainty intervals are provided in Tables S5 and S6, Supplemental Digital Content 5. TBI = traumatic brain injury, YLDs = years lived with disability.

Historical absolute numbers generally increased from 1990 to 2021, whereas projected age-standardized rates showed a declining pattern through 2050 (Fig. 6 and Fig. S4, Supplemental Digital Content 4). Figure 6 presents central projected trajectories for visual clarity, while the corresponding 95% UIs for the 2050 projections are provided in Tables S5 and S6, Supplemental Digital Content 5.

**Figure 7.**
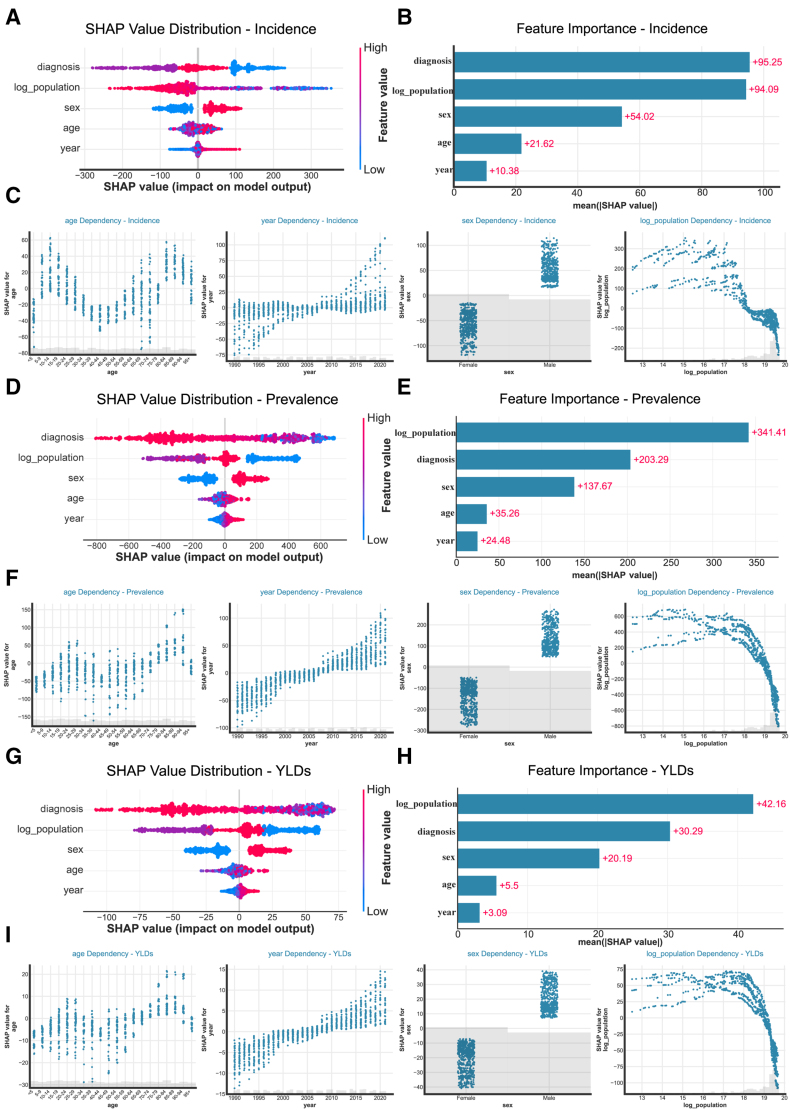
SHAP interpretation of XGBoost model predictions for TBI incidence (A–C), prevalence (D–F), and YLDs (G–I) among individuals aged 2.5 to 97.5 years. Beeswarm plots show the direction and magnitude of feature contributions; feature-importance plots rank predictors by mean absolute SHAP values; dependence plots show nonlinear associations between predictors and predicted burden. Approximate model-derived transition values from the SHAP dependence plots included age 5 to 24 and 80 to 94 years for incidence, age >75 years for prevalence and YLDs, calendar year around 2005, and natural log-transformed population size around 19.1. SHAP = SHapley Additive exPlanations, TBI = traumatic brain injury, YLDs = years lived with disability.

SHAP analysis was used to interpret XGBoost predictions for TBI incidence, prevalence, and YLDs among individuals aged 2.5 to 97.5 years (Fig. 7). Beeswarm plots illustrated the direction and magnitude of feature contributions, and feature-importance plots ranked predictors according to mean absolute SHAP values. Age showed nonlinear associations with TBI burden. For incidence, positive SHAP contributions were mainly observed at approximately 5 to 24 years and 80 to 94 years, suggesting a bimodal age-related pattern. For prevalence and YLDs, age-related SHAP contributions shifted toward positive values after approximately 75 years of age. The calendar year showed an approximate transition around 2005, after which SHAP contributions became more positive. For population size, SHAP values changed markedly when the natural log-transformed population size was approximately 19.1, corresponding to roughly 2.0 × 10^8^ people, above which SHAP contributions tended to become more negative. Male sex was associated with higher predicted incidence, prevalence, and YLDs. These threshold values were derived from SHAP dependence plots and should be interpreted as model-based transition points rather than causal or clinical thresholds.

**Figure 8.**
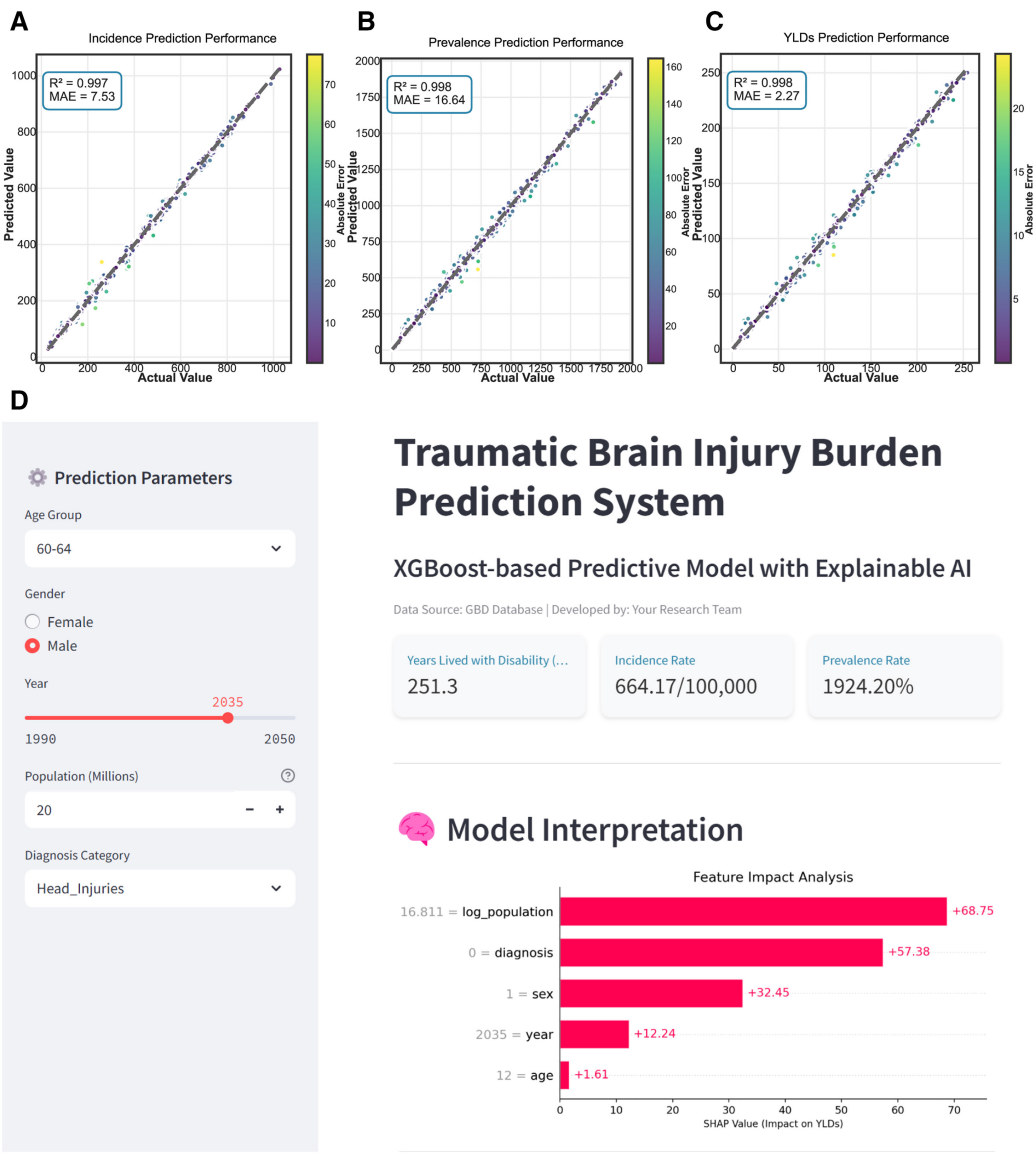
Internal cross-validation and web deployment of the XGBoost model for population-level TBI burden prediction. (A–C) Scatter plots comparing observed and predicted values under cross-validation for incidence, prevalence, and YLDs. Cross-validated *R*^2^ and MAE values indicate internal validation performance within the GBD-derived aggregate-data framework. (D) Interactive Streamlit interface for research and public-health exploration of TBI burden prediction and SHAP visualization. The tool is not intended for individual clinical decision-making, diagnosis, treatment selection, prognosis estimation, or patient-level risk stratification. GBD = Global Burden of Disease, MAE = mean absolute error, SHAP = SHapley Additive exPlanations, TBI = traumatic brain injury, YLDs = years lived with disability.

Figure 8 shows the model’s internal cross-validation results and web deployment. In cross-validation, the scatter plots showed close agreement between observed and predicted values, with cross-validated *R*^2^ values of 0.997 to 0.998 and MAE values of 7.53 for incidence, 16.64 for prevalence, and 2.27 for YLDs. These metrics indicate good internal consistency under cross-validation within the GBD-derived aggregate-data framework. The Streamlit web tool provides a research-use-only interface for exploring population-level TBI burden predictions and SHAP visualizations; it is not intended for clinical decision-making.

## 4. Discussion

In this study, we evaluated the global, regional, and national burden of TBI from 1990 to 2021 using GBD 2021 estimates and projected future burden to 2050 using population-level modeling and interpretable machine learning. Several key patterns emerged. First, the absolute burden of TBI increased substantially over the past 3 decades, whereas age-standardized incidence, prevalence, and YLD rates generally declined. Second, men consistently experienced a higher burden of TBI than women, although sex differences varied across age groups and regions. Third, age-specific analyses suggested a shift of TBI burden toward older populations, with the APC model identifying approximately 52.5 years as a transition point. Fourth, the association between SDI and age-standardized YLD rate showed an inverted U-shaped pattern, and health inequalities narrowed over time but remained evident. Fifth, regional heterogeneity persisted, including ASIR increases of 14.34% (95% UI: 9.59–20.65) in the Caribbean and 15.55% (95% UI: 11.65–19.05) in Oceania. Finally, XGBoost-SHAP modeling showed good internal cross-validated agreement between observed and predicted values and helped identify age, sex, calendar year, and population size as important predictors of TBI burden.

Our findings are broadly consistent with previous GBD-based studies, showing that the absolute number of TBI cases has increased over time, whereas age-standardized rates have tended to decline.^[6–8]^ Prior studies, including the GBD neurological disorders study and the recent analysis by Zhong et al, reported substantial global and regional TBI burden and highlighted important differences across countries and sociodemographic settings.^[3,8]^ The present study extends these previous analyses by using updated GBD 2021 estimates, incorporating age–sex–SDI stratification, examining temporal patterns with APC modeling, assessing socioeconomic disparities using inequality metrics and frontier analysis, and applying interpretable machine learning to support prediction and interpretation. Therefore, rather than replacing previous GBD-based studies, our work adds updated evidence on the demographic, socioeconomic, and predictive dimensions of TBI burden.

The divergent pattern between increasing absolute numbers and declining age-standardized rates is important. The increase in incident cases, prevalent cases, and YLDs suggests that TBI continues to impose a growing total burden on healthcare systems and societies. However, the decline in age-standardized rates indicates that changes in population size and age structure may partly explain the increasing absolute burden. This pattern also suggests that prevention, trauma care, and rehabilitation may have improved in many settings, although such gains are not evenly distributed.^[1,3,14]^ For public health planning, both absolute numbers and standardized rates should be considered. Absolute counts reflect the total demand for emergency care, neurosurgical capacity, rehabilitation, and long-term support, whereas age-standardized rates provide a clearer indication of underlying risk after accounting for demographic change.

Age was one of the most important dimensions of TBI burden in this study. The APC analysis indicated a transition around 52.5 years, after which the burden tended to increase with age. This finding supports the growing importance of TBI among older adults. In aging populations, fall-related injuries are likely to play an increasingly important role in TBI burden.^[9,10]^ However, given the ecological and observational nature of this study, these findings should be interpreted as associations rather than causal relationships. In younger and middle-aged populations, occupational exposure, road traffic injuries, and high-risk activities may contribute to sex- and age-specific differences. Therefore, TBI prevention strategies should be age-specific: fall-prevention programs, home-safety modification, osteoporosis and frailty management, and medication review may be particularly relevant for older adults, while road safety, helmet use, workplace protection, and sports safety remain important for younger populations.

The persistent sex difference in TBI burden also deserves attention. Men had higher incidence, prevalence, and YLD burden than women during the study period. This finding is consistent with previous epidemiological evidence and may be associated with differences in occupational exposure, road traffic exposure, risk-taking behaviors, and injury mechanisms.^[11,12]^ Nevertheless, sex differences were not uniform across regions and age groups. In some low-resource settings, sex disparities may also be influenced by differences in healthcare access, injury reporting, and rehabilitation availability. These findings suggest that sex-specific prevention and care strategies may be useful. For example, occupational safety programs and traffic injury prevention may be particularly important for working-age men, whereas fall prevention and postinjury rehabilitation access should be strengthened for both sexes among older adults.

Socioeconomic development was closely related to the burden of TBI. The inverted U-shaped relationship between SDI and age-standardized YLD rate suggests that TBI burden may increase during certain stages of socioeconomic transition before declining at higher development levels. This pattern may reflect changing injury exposure, motorization, urbanization, occupational risk, trauma-system capacity, and rehabilitation availability.^[13]^ In low-SDI regions, the change in age-standardized YLD rate was 1.14%, but the 95% UI crossed zero, indicating no statistically significant change. Therefore, this result should not be interpreted as evidence of an increasing trend. However, the relatively limited decline in low-SDI settings compared with higher-SDI settings suggests that low-resource regions may not have benefited equally from improvements in trauma prevention, acute care, and rehabilitation. Strengthening emergency medical systems, access to neurotrauma care, rehabilitation services, and injury surveillance should therefore be prioritized in these settings.

Regional heterogeneity was another important finding. Although many regions experienced declines in age-standardized rates, the Caribbean and Oceania showed increases in several TBI burden indicators; for incidence specifically, ASIR increased by 14.34% (95% UI: 9.59–20.65) in the Caribbean and by 15.55% (95% UI: 11.65–19.05) in Oceania. These regional differences may reflect variations in injury patterns, demographic change, healthcare infrastructure, surveillance quality, and access to post-injury care.^[13]^ The finding that health inequalities narrowed over time but persisted also suggests that global progress in TBI prevention and treatment has been uneven. Regions with rising or persistently high burden should be prioritized for targeted public health interventions. These may include strengthening trauma registries, improving prehospital transport, expanding neurosurgical and rehabilitation capacity, and developing region-specific injury-prevention policies.

The projection results provide additional evidence for planning future TBI-related healthcare needs. The BAPC-based projections suggested that age-standardized incidence, prevalence, and YLD rates may continue to decline through 2050. However, these projections should be interpreted cautiously because future TBI burden may be influenced by demographic aging, road-safety policies, urbanization, conflict, occupational patterns, healthcare access, and improvements in surveillance. Even if age-standardized rates decline, older populations and vulnerable regions may continue to account for a substantial share of TBI-related care needs. Therefore, policymakers should not interpret declining standardized rates as a reason to reduce investment in TBI prevention and care. Instead, resource allocation should be adjusted according to age structure, regional burden, SDI level, and healthcare capacity.

The XGBoost-SHAP framework provided an interpretable machine-learning perspective on TBI burden prediction. The model showed strong internal cross-validated agreement between observed and predicted values, and SHAP analysis identified key contributors to model predictions. The SHAP results also suggested nonlinear relationships between predictors and TBI burden, supporting the value of interpretable machine learning for exploring complex population-level epidemiological data.^[23,25,28]^ General principles from prediction-model development, validation, hyperparameter optimization, and explainable tree-based modeling further support cautious interpretation of the model as an exploratory population-level tool rather than an independent clinical prediction instrument.^[24,26,27]^ However, this model should be understood as a research and public-health exploration tool rather than a clinical decision-making instrument. Because the model was developed using population-level GBD estimates, it cannot be directly applied to diagnose, prognosticate, or guide treatment for individual patients. Future individual-level models may improve performance and clinical relevance by incorporating clinical severity scores, neuroimaging features, physiological indicators, blood biomarkers, inflammatory markers, gut–brain axis indicators, rehabilitation data, and multimodal sources such as clinical text or video-derived injury information.^[29–31]^

The present findings have several public health implications. First, prevention strategies should be tailored by age and injury mechanism, which is consistent with broader evidence on the global burden, changing epidemiology, surveillance patterns, and clinical consequences of TBI.^[32–38]^ Fall prevention should be strengthened for older adults, including community screening for fall risk, home-safety assessment, balance and strength training, and management of frailty-related risk factors. Second, traffic and occupational safety interventions remain important for younger and working-age populations, particularly males. These interventions may include helmet promotion, enforcement of road-safety regulations, workplace safety training, and targeted injury-prevention education. Third, low-resource settings require investment in trauma systems, including prehospital care, emergency transport, neurosurgical capacity, intensive care, and rehabilitation services. Fourth, regions with increasing or persistently high burden, such as the Caribbean and Oceania, should be prioritized for surveillance strengthening and locally adapted prevention programs. Finally, public health planning should consider both absolute burden and age-standardized rates to avoid underestimating future service needs in aging societies.

Several limitations should be acknowledged. First, this study was based on population-level GBD estimates; therefore, ecological fallacy is possible, and associations observed at the population level may not apply to individual patients. Second, although cross-validation was performed for internal model evaluation, the model and projections were not externally validated using independent datasets. Therefore, the reported cross-validated *R*^2^ and MAE should be interpreted as internal performance metrics within the GBD-derived aggregate-data framework rather than evidence of generalizability to independent real-world datasets. Third, formal comparison with alternative forecasting models such as Autoregressive integrated moving average model or linear-trend models was not performed, which may limit assessment of the relative predictive advantage of XGBoost. Fourth, although GBD estimates include UIs, uncertainty from GBD inputs was not fully propagated through all secondary APC, frontier, inequality, BAPC, and machine-learning analyses. Fifth, the analysis did not fully adjust for healthcare-system factors such as trauma-center density, emergency medical service capacity, neurosurgical availability, rehabilitation coverage, or insurance systems, all of which may influence observed TBI burden and outcomes. Sixth, temporal and regional differences may be affected by surveillance bias, including changes in diagnostic capacity, coding practices, injury reporting, and healthcare access over time. Seventh, the XGBoost-SHAP model was developed for population-level prediction and should not be used for individual clinical decision-making. Eighth, Figure 6 primarily displays point estimates to maintain readability across multiple projection panels; therefore, projection uncertainty should be interpreted using the 95% UIs reported in Tables S5 and S6, Supplemental Digital Content 5. Finally, because this study used secondary GBD data, the findings depend on the quality and assumptions of the original data sources and estimation procedures.

## 5. Conclusion

In summary, the global burden of TBI showed a dual pattern from 1990 to 2021: absolute numbers increased, whereas age-standardized rates generally declined. The burden remained higher among males, shifted toward older populations, and varied substantially by SDI and region. Although socioeconomic inequalities narrowed, they persisted, and certain regions continued to show increasing rates. Population-level projection and interpretable machine learning may help identify future burden patterns and support public health planning. Targeted prevention, strengthened trauma care, improved rehabilitation, and risk-stratified resource allocation are needed to reduce the future burden of TBI, particularly among older adults and populations in low-resource settings.

## Acknowledgments

This study was generously supported by Jingding Medical Tech, to whom we extend our sincere gratitude. We especially thank them for providing authorization and technical support for the JD_GBDR software. The team at Jingding Medical Tech offered invaluable assistance in data processing.

## Author contributions

**Conceptualization:** Shiying Dong, Ming Qiu, Mintao Li, Fangbing Li.

**Data curation:** Shiying Dong, Fangbing Li, Jiangyuan Yuan.

**Investigation:** Mintao Li, Di Wu, Jiangyuan Yuan, Chenrui Wu.

**Formal analysis:** Meng Nie, Jiangyuan Yuan.

**Project administration:** Meng Nie.

**Methodology:** Jiangyuan Yuan, Chenrui Wu.

**Funding acquisition:** Chenrui Wu.

**Software:** Fei Song.

**Visualization:** Fei Song.

**Validation:** Chuang Gao.

**Resources:** Rongcai Jiang.

**Supervision:** Rongcai Jiang.

**Writing – review & editing:** Rongcai Jiang.

**Writing – original draft:** Shiying Dong, Ming Qiu.




















