# Supplementary material for: Global burden of traumatic brain injury from 1990 to 2021 and projections to 2050: A GBD 2021–based study using interpretable machine learning
Source: Medicine (Baltimore). 2026 Jul 24;105(30):e49918. doi: 10.1097/MD.0000000000049918 (PMC13406132; doi:10.1097/MD.0000000000049918)
Supplement: Supplementary file 1 [file medi-105-e49918-s001.docx]

**Supplementary Table S1.** The incidence cases and age-standardized incidence rate of traumatic brain injury in 1990 and 2021, and its temporal trends from 1990 to 2021 ASR Age-standardized rate

| Characteristics | 1990 | | |  | 2021 | | | 1990-2021 |
| --- | --- | --- | --- | --- | --- | --- | --- | --- |
|  | Incidence cases No.x10'5 (95% UI) | ASR per 100,000 No. (95% UI) | Male/Female |  | Incidence cases No.x10'5 (95% UI) | ASR per 100,000 No. (95% UI) | Male/Female | Percentage change in age-standardised rates |
| Global | 17001269 (14859015, 19464639) | 324.43(283.29, 370.12) | 2.08 |  | 20837466 (18128307, 23839394) | 259.02(225.50, 296.20) | 2.04 | -20.16%(-22.55, -17.40) |
| High SDl | 3524337 (2940300, 4306393) | 398.98(333.84, 491.21) | 1.99 |  | 3549306 (2894216, 4298865) | 305.28(254.54, 369.15) | 1.84 | -23.48%(-26.29, -20.73) |
| High-middle SDl | 4364788 (3795085, 4997370) | 405.60(353.03, 465.01) | 2.39 |  | 4463101 (3843588, 5142883) | 320.23(277.41, 370.22) | 2.21 | -21.05%(-23.81, -18.49) |
| Middle SDl | 5029618 (4434654, 5693728) | 300.79(264.17, 337.96) | 2.05 |  | 6465390 (5624880, 7383688) | 258.59(225.07, 295.03) | 2.10 | -14.03%(-17.31, -10.73) |
| Low-middle SDl | 2865759 (2535090, 3239564) | 271.00(239.01, 305.88) | 1.73 |  | 4205683 (3708320, 4760273) | 234.40(205.51, 266.65) | 1.77 | -13.51%(-15.95, -11.18) |
| Low SDI | 1195663 (1030975, 1426572) | 254.29(223.51, 294.78) | 2.01 |  | 2133439 (1877862, 2466373) | 216.88(191.54, 246.74) | 1.93 | -14.71%(-18.54, -11.06) |
| Andean Latin America | 104976 (92262, 120576) | 275.47(243.83, 313.82) | 2.88 |  | 152864 (133550, 173685) | 229.66(200.53, 260.20) | 2.69 | -16.63%(-23.42, -10.64) |
| Australasia | 114283 (87639, 152342) | 583.03(440.29, 789.61) | 1.82 |  | 139248 (105001, 182132) | 479.03(355.14, 652.10) | 1.59 | -17.84%(-21.79, -13.48) |
| Caribbean | 91006 (79930, 102626) | 261.88(229.23, 295.28) | 2.31 |  | 144663 (125192, 163811) | 299.44(259.92, 338.81) | 2.02 | 14.34% (9.59, 20.65) |
| Central Asia | 257707 (226936, 292068) | 365.19(324.10, 412.41) | 3.22 |  | 269848 (237410, 305727) | 280.47(246.76, 318.55) | 2.91 | -23.20%(-24.57, -21.94) |
| Central Europe | 824838 (700734, 951181) | 654.48(557.54, 755.66) | 2.44 |  | 601947 (506536, 701295) | 478.59(405.13, 560.62) | 2.41 | -26.87%(-28.58, -24.93) |
| Central Latin America | 730579 (635119, 832663) | 451.25(396.18, 510.49) | 2.85 |  | 810783 (714280, 915908) | 318.27(279.80, 360.77) | 2.77 | -29.47%(-30.90, -28.23) |
| Central Sub-Saharan Africa | 107892 (95210, 123224) | 204.38(181.30, 230.75) | 1.88 |  | 224835 (200748, 253866) | 183.11(163.45, 204.88) | 2.02 | -10.41%(-13.16, -7.84) |
| East Asia | 3061528 (2657684, 3540501) | 258.43(224.65, 294.90) | 1.85 |  | 4313633 (3671116, 4998871) | 262.65(224.70, 304.65) | 1.99 | 1.63% (-3.71, 6.45) |
| Eastern Europe | 1597158 (1392361, 1821789) | 698.30(608.20, 797.56) | 3.16 |  | 1115069 (963661, 1274684) | 522.41(454.09, 602.15) | 3.07 | -25.19%(-27.54, -22.84) |
| Eastern Sub-Saharan Africa | 535434 (430002, 708467) | 283.27(233.28, 361.10) | 2.52 |  | 620976 (544555, 720066) | 167.41(148.15, 190.52) | 2.23 | -40.90%(-49.93, -31.33) |
| High-income Asia Pacifc | 662604 (540910, 824064) | 386.13(313.80, 485.20) | 1.98 |  | 446418 (357113, 551239) | 243.07(191.46, 312.81) | 1.75 | -37.05%(-40.56, -33.64) |
| High-income North America | 1041429 (858750, 1285968) | 368.11(304.36, 456.48) | 1.88 |  | 1100573 (895390, 1336999) | 269.16(222.73, 324.36) | 1.64 | -26.88%(-30.98, -22.39) |
| North Africa and Middle East | 1297463 (1148050, 1471208) | 384.67(341.68, 434.23) | 1.97 |  | 2050666 (1801675, 2347332) | 333.41(293.35, 379.97) | 2.35 | -13.33%(-18.28, -8.05) |
| Oceania | 11708 (10404, 13074) | 198.15(176.46, 220.84) | 1.48 |  | 28899 (25664, 32512) | 228.96(203.66, 258.52) | 1.30 | 15.55%(11.65, 19.05) |
| South Asia | 2517620 (2194621, 2883746) | 267.66(228.70, 308.52) | 1.43 |  | 4149217 (3543281, 4823244) | 242.98(203.78, 283.05) | 1.40 | -9.22%(-12.60, -5.81) |
| Southeast Asia | 1161655 (1032055, 1316039) | 258.45(228.54, 291.82) | 2.14 |  | 1387482 (1217084, 1564118) | 201.14(175.30, 227.42) | 2.14 | -22.17%(-25.37, -18.79) |
| Southern Latin America | 191701 (146863, 261616) | 382.27(294.16, 520.00) | 2.42 |  | 238178 (184978, 321973) | 362.68(280.07, 495.88) | 2.13 | -5.12% (-7.88, -1.80) |
| Southern Sub-Saharan Africa | 176839 (152480, 202646) | 357.97(309.20, 409.65) | 2.81 |  | 203466 (176836, 229679) | 248.70(217.66, 279.55) | 3.41 | -30.52%(-32.85, -27.76) |
| Tropical Latin America | 661138 (568152, 770714) | 440.61(381.20, 509.76) | 3.11 |  | 827052 (722081, 948791) | 351.28(304.73, 402.79) | 3.23 | -20.27%(-22.56, -18.15) |
| Western Europe | 1543939 (1256345, 1898870) | 405.32(330.02, 504.93) | 2.15 |  | 1323853 (1043018, 1667371) | 300.27(234.34, 380.98) | 1.77 | -25.92%(-30.02, -21.69) |
| Western Sub-Saharan Africa | 309774 (274882, 348015) | 172.35(154.13, 192.29) | 1.92 |  | 687797 (612198, 774831) | 161.97(144.21, 180.89) | 2.03 | -6.02% (-8.09, -3.60) |
