# Supplementary figures and images for: Global burden of traumatic brain injury from 1990 to 2021 and projections to 2050: A GBD 2021–based study using interpretable machine learning
Source: Medicine (Baltimore). 2026 Jul 24;105(30):e49918. doi: 10.1097/MD.0000000000049918 (PMC13406132; doi:10.1097/MD.0000000000049918)

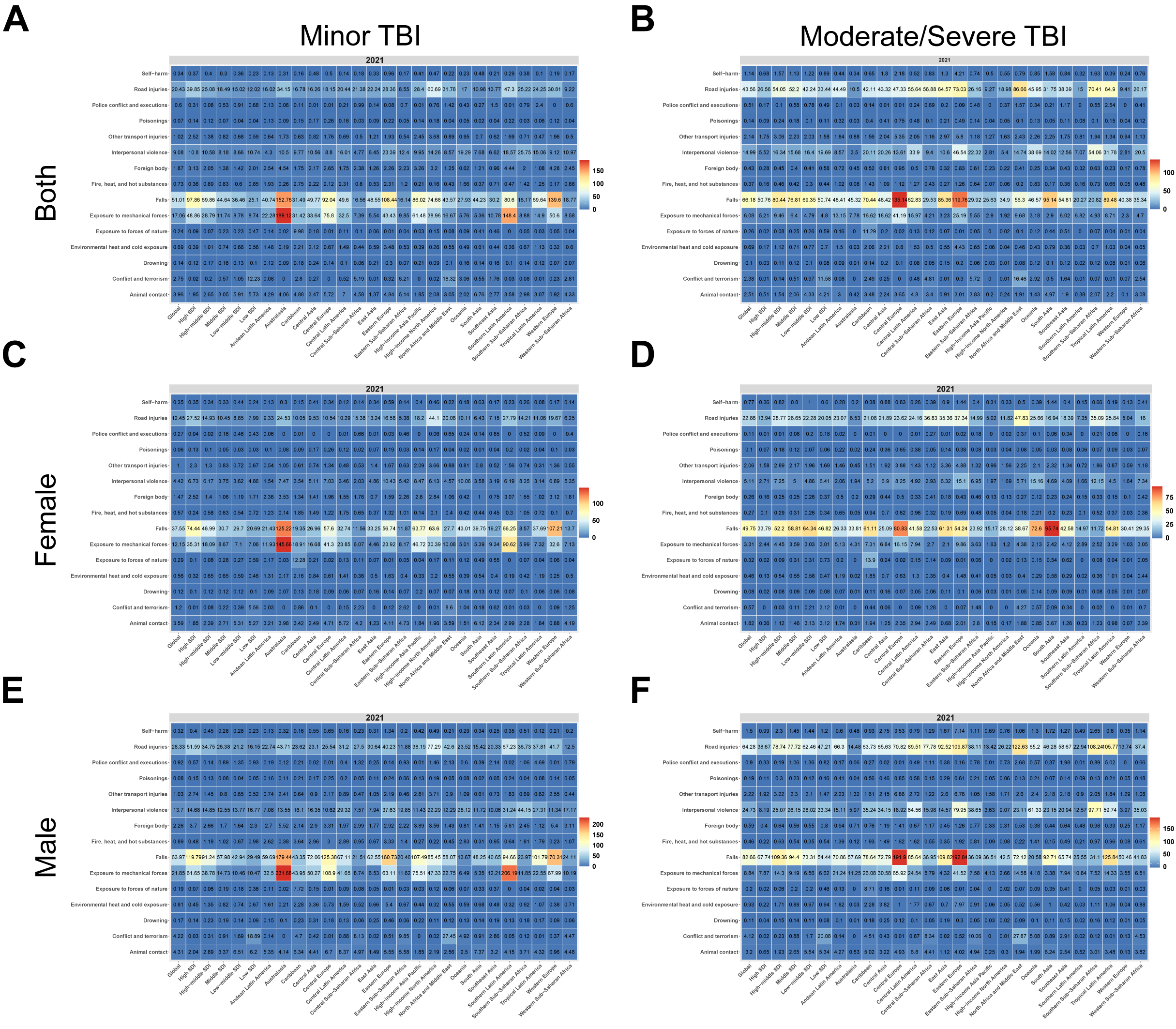

Supplement: Supplementary file 2 [file medi-105-e49918-s002.tif]

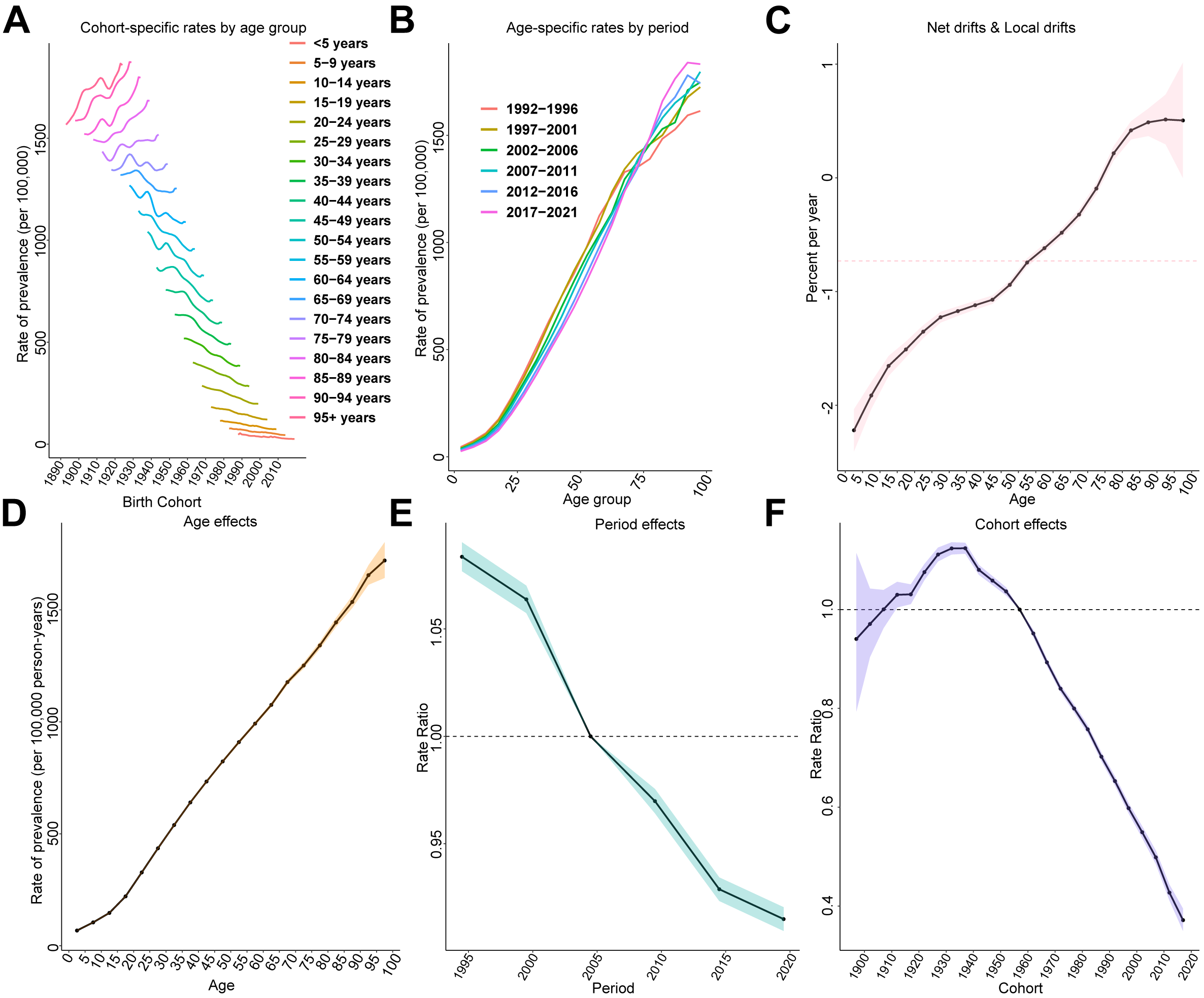

Supplement: Supplementary file 3 [file medi-105-e49918-s003.tif]

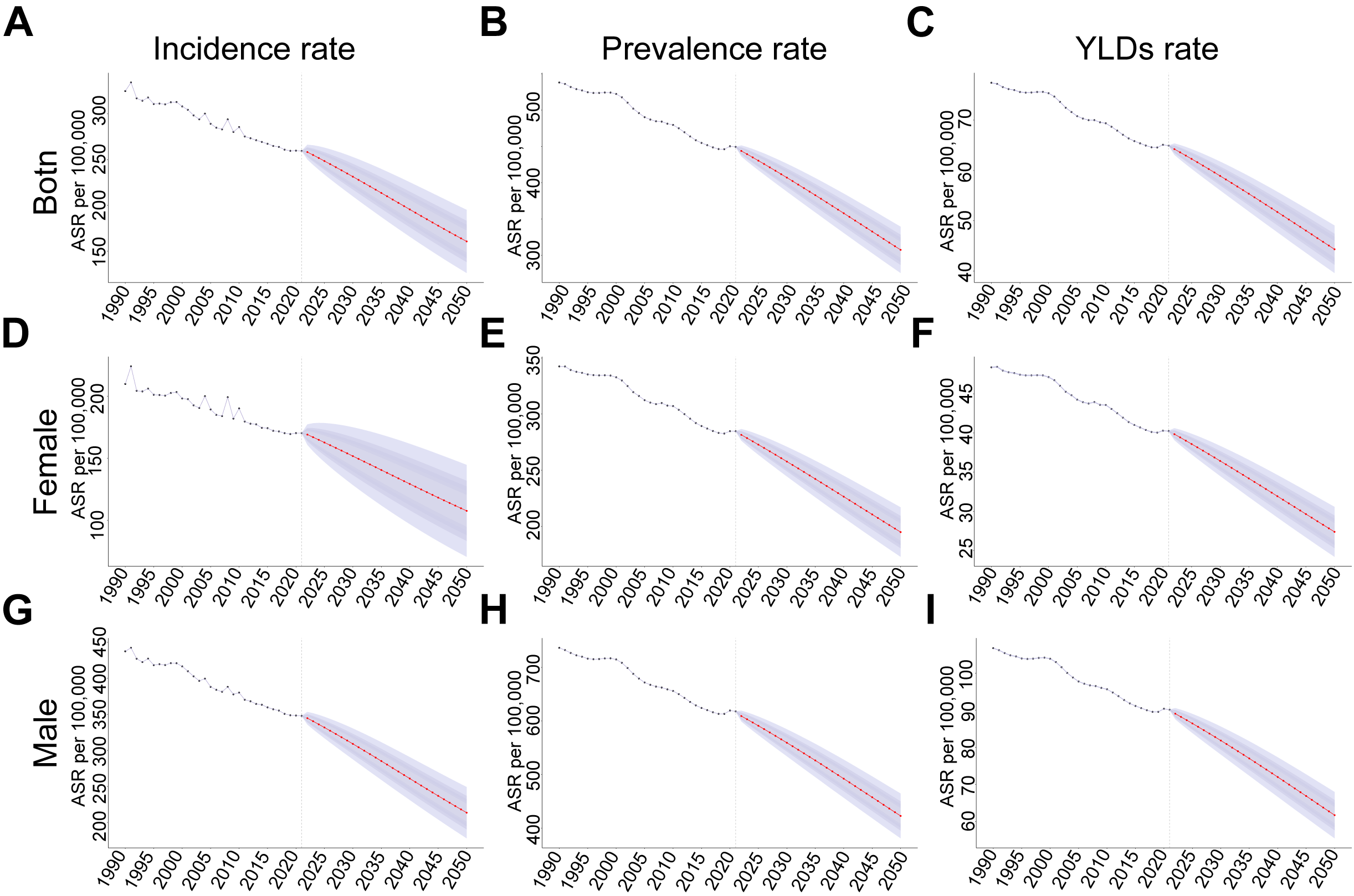

Supplement: Supplementary file 4 [file medi-105-e49918-s004.tif]

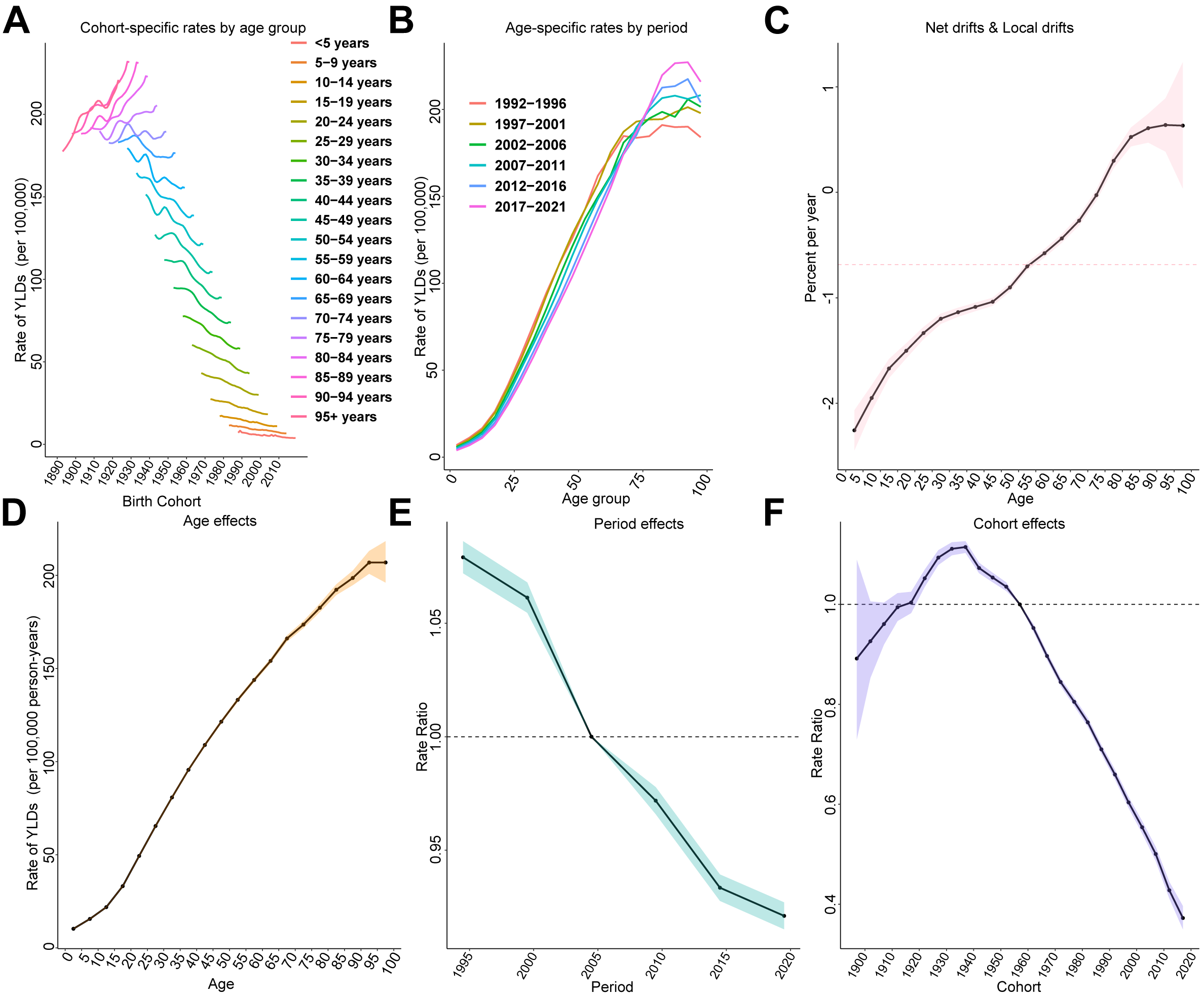

Supplement: Supplementary file 10 [file medi-105-e49918-s010.tif]
