# Supplementary material for: Global burden of traumatic brain injury from 1990 to 2021 and projections to 2050: A GBD 2021–based study using interpretable machine learning
Source: Medicine (Baltimore). 2026 Jul 24;105(30):e49918. doi: 10.1097/MD.0000000000049918 (PMC13406132; doi:10.1097/MD.0000000000049918)
Supplement: Supplementary file 5 [file medi-105-e49918-s005.docx]

**Supplementary Table S5.** Historical and projected number of incidence, prevalence, and YLDs cases for TBI for all ages by sex and both, 1990-2050.

| **Year** | **Incidence cases (×10⁵) (95% UI)** | |  |  | **Prevalence cases (×10⁵) (95% UI)** | |  |  | **YLDs cases (×10⁵) (95% UI)** | | |
| --- | --- | --- | --- | --- | --- | --- | --- | --- | --- | --- | --- |
|  | **Male** | **Female** | **Both** |  | **Male** | **Female** | **Both** |  | **Male** | **Female** | **Both** |
| 1990 | 11750980(11741267, 11760692) | 5556911(5550277, 5563544) | 17329076(17317335, 17340816) |  | 19778620(19765489, 19791751) | 9066338(9057643, 9075032) | 28691898(28676286, 28707511) |  | 2867876(2862922, 2872830) | 1281195(1277944, 1284447) | 4129537(4123643, 4135430) |
| 1991 | 12068977(12059145, 12078808) | 6021893(6015010, 6028776) | 18104154(18092178, 18116130) |  | 19981207(19968045, 19994369) | 9202096(9193359, 9210833) | 29029674(29014006, 29045342) |  | 2899056(2894093, 2904018) | 1302190(1298925, 1305456) | 4181628(4175714, 4187541) |
| 1992 | 11834019(11824286, 11843752) | 5572929(5566294, 5579564) | 17423603(17411845, 17435361) |  | 20135863(20122679, 20149047) | 9251512(9242766, 9260258) | 29233080(29217386, 29248773) |  | 2920974(2916003, 2925944) | 1308143(1304876, 1311410) | 4209129(4203207, 4215051) |
| 1993 | 11876777(11867032, 11886523) | 5637814(5631146, 5644482) | 17528242(17516456, 17540028) |  | 20304640(20291429, 20317850) | 9336947(9328177, 9345717) | 29486026(29470295, 29501756) |  | 2946615(2941632, 2951597) | 1320672(1317394, 1323949) | 4247023(4241084, 4252961) |
| 1994 | 12169794(12159940, 12179648) | 5774715(5767972, 5781458) | 17956359(17944443, 17968275) |  | 20503152(20489908, 20516397) | 9435288(9426489, 9444088) | 29781423(29765646, 29797200) |  | 2977742(2972744, 2982741) | 1335285(1331995, 1338575) | 4292499(4286540, 4298458) |
| 1995 | 12070945(12061134, 12080757) | 5710521(5703816, 5717225) | 17789478(17777620, 17801336) |  | 20668968(20655696, 20682240) | 9517451(9508628, 9526273) | 30026709(30010896, 30042522) |  | 3001038(2996029, 3006048) | 1346820(1343521, 1350120) | 4326771(4320798, 4332744) |
| 1996 | 12263435(12253553, 12273316) | 5778341(5771601, 5785082) | 18047031(18035097, 18058966) |  | 20902875(20889555, 20916195) | 9623108(9614252, 9631963) | 30361770(30345899, 30377641) |  | 3035800(3030770, 3040830) | 1361881(1358568, 1365193) | 4375882(4369886, 4381879) |
| 1997 | 12387085(12377159, 12397012) | 5838978(5832205, 5845750) | 18228191(18216203, 18240179) |  | 21180867(21167487, 21194248) | 9742230(9733337, 9751123) | 30753349(30737408, 30769291) |  | 3076837(3071782, 3081892) | 1379572(1376243, 1382900) | 4433772(4427747, 4439797) |
| 1998 | 12620993(12610980, 12631007) | 5977358(5970511, 5984205) | 18597216(18585116, 18609317) |  | 21481993(21468546, 21495441) | 9870668(9861733, 9879603) | 31177398(31161378, 31193417) |  | 3121682(3116599, 3126764) | 1397985(1394641, 1401330) | 4496265(4490209, 4502321) |
| 1999 | 12774053(12763984, 12784122) | 6075004(6068104, 6081903) | 18844862(18832689, 18857035) |  | 21755787(21742284, 21769290) | 9989122(9980152, 9998092) | 31564751(31548666, 31580835) |  | 3162840(3157734, 3167945) | 1415062(1411703, 1418421) | 4553829(4547746, 4559912) |
| 2000 | 12798240(12788170, 12808310) | 5998707(5991853, 6005561) | 18792580(18780430, 18804729) |  | 21959570(21946033, 21973106) | 10071135(10062145, 10080124) | 31846809(31830686, 31862932) |  | 3192953(3187832, 3198073) | 1426716(1423349, 1430083) | 4595013(4588914, 4601112) |
| 2001 | 12749026(12738981, 12759071) | 6058810(6051927, 6065693) | 18800845(18788701, 18812989) |  | 22037416(22023887, 22050945) | 10114247(10105258, 10123236) | 31964829(31948712, 31980947) |  | 3204712(3199593, 3209831) | 1432983(1429615, 1436350) | 4612616(4606518, 4618714) |
| 2002 | 12686730(12676718, 12696742) | 5982530(5975694, 5989367) | 18661640(18649550, 18673730) |  | 22010519(21997030, 22024008) | 10092104(10083143, 10101064) | 31913966(31897897, 31930034) |  | 3201332(3196227, 3206437) | 1429606(1426250, 1432963) | 4605506(4599425, 4611587) |
| 2003 | 12648288(12638299, 12658277) | 5995898(5989059, 6002737) | 18635138(18623065, 18647210) |  | 21942660(21929222, 21956097) | 10055023(10046097, 10063948) | 31807782(31791775, 31823789) |  | 3192394(3187307, 3197481) | 1424372(1421028, 1427716) | 4591008(4584950, 4597067) |
| 2004 | 12904228(12894147, 12914308) | 6376228(6369183, 6383274) | 19268333(19256070, 19280597) |  | 21936205(21922802, 21949608) | 10078765(10069849, 10087681) | 31823711(31807737, 31839685) |  | 3193055(3187979, 3198132) | 1429090(1425747, 1432433) | 4596320(4590271, 4602368) |
| 2005 | 12697532(12687541, 12707523) | 6115776(6108879, 6122674) | 18802344(18790237, 18814451) |  | 21976075(21962690, 21989459) | 10092120(10083217, 10101023) | 31875371(31859419, 31891323) |  | 3198923(3193853, 3203993) | 1430092(1426755, 1433430) | 4602847(4596807, 4608888) |
| 2006 | 12727254(12717262, 12737247) | 6056772(6049913, 6063631) | 18774187(18762099, 18786275) |  | 22118629(22105235, 22132023) | 10159390(10150476, 10168303) | 32083833(32067866, 32099800) |  | 3220172(3215096, 3225247) | 1439041(1435700, 1442382) | 4632817(4626770, 4638864) |
| 2007 | 12805304(12795291, 12815318) | 6108168(6101285, 6115051) | 18903933(18891814, 18916053) |  | 22299194(22285780, 22312608) | 10254100(10245166, 10263034) | 32357677(32341679, 32373675) |  | 3247959(3242873, 3253045) | 1452894(1449544, 1456244) | 4674183(4668122, 4680244) |
| 2008 | 13205573(13195412, 13215735) | 6702764(6695559, 6709969) | 19895133(19882708, 19907558) |  | 22538775(22525322, 22552227) | 10426817(10417828, 10435806) | 32767966(32751905, 32784028) |  | 3285153(3280049, 3290257) | 1479267(1475894, 1482641) | 4737706(4731617, 4743795) |
| 2009 | 13030151(13020068, 13040233) | 6208642(6201714, 6215570) | 19231568(19219363, 19243773) |  | 22725981(22712506, 22739455) | 10494482(10485484, 10503481) | 33021522(33005435, 33037609) |  | 3312610(3307496, 3317724) | 1487637(1484261, 1491013) | 4773017(4766918, 4779115) |
| 2010 | 13277519(13267349, 13287689) | 6573570(6566445, 6580696) | 19843267(19830877, 19855657) |  | 22910886(22897391, 22924382) | 10619913(10610880, 10628945) | 33331603(33315477, 33347728) |  | 3341743(3336618, 3346869) | 1506941(1503550, 1510333) | 4821628(4815511, 4827745) |
| 2011 | 13091796(13081704, 13101888) | 6289337(6282375, 6296300) | 19375363(19363129, 19387598) |  | 22979125(22965644, 22992607) | 10641018(10631997, 10650038) | 33421256(33405146, 33437366) |  | 3351857(3346735, 3356979) | 1508689(1505303, 1512075) | 4833312(4827201, 4839423) |
| 2012 | 13174678(13164563, 13184794) | 6308835(6301869, 6315802) | 19479352(19467094, 19491611) |  | 23011546(22998089, 23025002) | 10641576(10632577, 10650576) | 33454791(33438710, 33470872) |  | 3357934(3352819, 3363049) | 1508927(1505549, 1512306) | 4839652(4833550, 4845754) |
| 2013 | 13204424(13194303, 13214546) | 6371276(6364281, 6378272) | 19571640(19559359, 19583921) |  | 23024115(23010687, 23037543) | 10632200(10623225, 10641176) | 33458156(33442110, 33474202) |  | 3361292(3356185, 3366398) | 1507677(1504307, 1511047) | 4841680(4835589, 4847771) |
| 2014 | 13325479(13315318, 13335641) | 6349745(6342768, 6356721) | 19674378(19662073, 19686684) |  | 23070845(23057435, 23084256) | 10624164(10615212, 10633116) | 33497031(33481010, 33513051) |  | 3369480(3364377, 3374582) | 1506416(1503055, 1509777) | 4848512(4842429, 4854595) |
| 2015 | 13354607(13344438, 13364777) | 6417459(6410450, 6424468) | 19772073(19759742, 19784404) |  | 23175135(23161723, 23188547) | 10658781(10649833, 10667728) | 33635706(33619685, 33651726) |  | 3385273(3380169, 3390378) | 1511162(1507802, 1514521) | 4869018(4862934, 4875102) |
| 2016 | 13418012(13407823, 13428201) | 6416558(6409557, 6423559) | 19837824(19825479, 19850169) |  | 23307732(23294310, 23321153) | 10704815(10695868, 10713763) | 33814655(33798624, 33830685) |  | 3404604(3399495, 3409714) | 1517970(1514610, 1521331) | 4895177(4889088, 4901266) |
| 2017 | 13508780(13498562, 13518999) | 6473935(6466910, 6480961) | 19988941(19976555, 20001327) |  | 23432025(23418598, 23445453) | 10750605(10741658, 10759551) | 33985513(33969475, 34001551) |  | 3423475(3418361, 3428589) | 1523834(1520474, 1527195) | 4919961(4913868, 4926053) |
| 2018 | 13487023(13476817, 13497228) | 6494274(6487245, 6501304) | 19989800(19977421, 20002180) |  | 23584264(23570822, 23597705) | 10808474(10799523, 10817424) | 34196189(34180135, 34212243) |  | 3445705(3440585, 3450825) | 1531478(1528116, 1534839) | 4949850(4943751, 4955949) |
| 2019 | 13558117(13547891, 13568344) | 6538960(6531913, 6546007) | 20108697(20096288, 20121106) |  | 23839964(23826481, 23853447) | 10905813(10896843, 10914784) | 34549467(34533365, 34565569) |  | 3483080(3477942, 3488217) | 1545091(1541722, 1548460) | 5000809(4994691, 5006928) |
| 2020 | 13676539(13666273, 13686805) | 6632774(6625681, 6639867) | 20322923(20310454, 20335392) |  | 24314289(24300700, 24327878) | 11096326(11087296, 11105357) | 35210470(35194246, 35226694) |  | 3549513(3544336, 3554690) | 1570127(1566737, 1573518) | 5091956(5085793, 5098119) |
| 2021 | 13772188(13761889, 13782487) | 6691946(6684824, 6699068) | 20479588(20467075, 20492101) |  | 24465383(24451774, 24478993) | 11194667(11185611, 11203722) | 35457495(35441241, 35473749) |  | 3569229(3564041, 3574416) | 1582422(1579021, 1585824) | 5123701(5117526, 5129876) |
| 2022 | 13912944(13560996, 14264891) | 6776684(6446832, 7106535) | 20777999(21422036, 21422036) |  | 24586791(24180401, 24993181) | 11285452(11077524, 11493381) | 35701109(35093402, 36308816) |  | 3596178(3535354, 3657002) | 1597715(1567119, 1628310) | 5168124(5077021, 5259226) |
| 2023 | 13872695(13419795, 14325595) | 6757912(6302377, 7213446) | 20718601(21583124, 21583124) |  | 24575630(24051212, 25100049) | 11278901(11008411, 11549392) | 35683813(34894302, 36473324) |  | 3595012(3516468, 3673557) | 1596583(1556943, 1636223) | 5165780(5047843, 5283716) |
| 2024 | 13824186(13285773, 14362599) | 6734954(6177604, 7292304) | 20645642(21691863, 21691863) |  | 24554532(23929858, 25179207) | 11266439(10943075, 11589802) | 35650442(34707146, 36593738) |  | 3592233(3498638, 3685829) | 1594578(1547277, 1641879) | 5160953(5020261, 5301645) |
| 2025 | 13765389(13150754, 14380024) | 6707742(6061435, 7354049) | 20557594(21763651, 21763651) |  | 24520150(23805690, 25234610) | 11248199(10877594, 11618805) | 35598389(34517634, 36679143) |  | 3587385(3480312, 3694458) | 1591723(1537570, 1645875) | 5153261(4992206, 5314315) |
| 2026 | 13697723(13013201, 14382244) | 6677065(5950163, 7403967) | 20457368(21808791, 21808791) |  | 24473408(23675964, 25270853) | 11226247(10812027, 11640468) | 35531329(34323654, 36739004) |  | 3580785(3461253, 3700316) | 1588323(1527844, 1648802) | 5143390(4963523, 5323257) |
| 2027 | 13622091(12872330, 14371852) | 6643169(5841714, 7444623) | 20346229(21832525, 21832525) |  | 24410106(23534448, 25285764) | 11197809(10742562, 11653057) | 35441223(34114098, 36768349) |  | 3571891(3440616, 3703166) | 1584041(1517611, 1650471) | 5130310(4932734, 5327886) |
| 2028 | 13539775(12728611, 14350939) | 6606411(5735138, 7477683) | 20225596(21838369, 21838369) |  | 24336822(23387236, 25286409) | 11164156(10670142, 11658170) | 35335899(33895905, 36775893) |  | 3561451(3419076, 3703827) | 1579016(1506963, 1651070) | 5114994(4900679, 5329308) |
| 2029 | 13449057(12579875, 14318238) | 6565760(5628737, 7502782) | 20091732(21823611, 21823611) |  | 24254399(23234616, 25274182) | 11124967(10594168, 11655766) | 35215611(33668478, 36762743) |  | 3549507(3396594, 3702420) | 1573179(1495792, 1650567) | 5097346(4867144, 5327548) |
| 2030 | 13348700(12424526, 14272874) | 6521318(5522074, 7520562) | 19944046(21788529, 21788529) |  | 24159036(23072379, 25245693) | 11080387(10514504, 11646271) | 35077298(33428056, 36726539) |  | 3535512(3372565, 3698460) | 1566536(1484064, 1649009) | 5076894(4831555, 5322233) |
| 2031 | 13240235(12263650, 14216821) | 6473967(5415522, 7532412) | 19785504(21737044, 21737044) |  | 24051717(22900893, 25202541) | 11032535(10432883, 11632187) | 34924539(33177209, 36671870) |  | 3519820(3347243, 3692396) | 1559411(1472048, 1646775) | 5054324(4794447, 5314201) |
| 2032 | 13124874(12098053, 14151696) | 6423956(5308991, 7538920) | 19617708(21671523, 21671523) |  | 23928908(22716194, 25141623) | 10978700(10346449, 11610951) | 34750177(32908257, 36592098) |  | 3501986(3320120, 3683853) | 1551473(1459389, 1643556) | 5028759(4754864, 5302654) |
| 2033 | 13003889(11928864, 14078914) | 6371480(5202480, 7540480) | 19441939(21593589, 21593589) |  | 23797887(22525594, 25070180) | 10920362(10256717, 11584008) | 34563197(32630195, 36496199) |  | 3482891(3292082, 3673700) | 1542905(1446276, 1639533) | 5001369(4713975, 5288763) |
| 2034 | 12875957(11754762, 13997152) | 6315635(5095125, 7536144) | 19255158(21500084, 21500084) |  | 23659200(22329628, 24988771) | 10857027(10163222, 11550831) | 34363242(32342694, 36383791) |  | 3462486(3263087, 3661884) | 1533593(1432603, 1634583) | 4971918(4671559, 5272278) |
| 2035 | 12740463(11575032, 13905894) | 6256545(4986947, 7526143) | 19057314(21391149, 21391149) |  | 23508974(22124492, 24893456) | 10788834(10066040, 11511628) | 34147055(32042495, 36251616) |  | 3440238(3232614, 3647863) | 1523554(1418375, 1628732) | 4939916(4627128, 5252705) |
| 2036 | 12598698(11390698, 13806697) | 6195146(4878623, 7511669) | 18851023(21269916, 21269916) |  | 23347615(21910319, 24784911) | 10717520(9966679, 11468360) | 33917037(31731504, 36102570) |  | 3416382(3200848, 3631916) | 1513041(1403816, 1622266) | 4905852(4581094, 5230610) |
| 2037 | 12451825(11202629, 13701022) | 6131594(4770193, 7492994) | 18637716(21138250, 21138250) |  | 23172231(21684017, 24660445) | 10640585(9862635, 11418534) | 33667023(31403407, 35930639) |  | 3390618(3167453, 3613782) | 1501776(1388640, 1614912) | 4869051(4532750, 5205352) |
| 2038 | 12301032(11011912, 13590152) | 6066167(4661858, 7470475) | 18418713(20997645, 20997645) |  | 22990785(21453438, 24528133) | 10559966(9755847, 11364086) | 33407374(31068383, 35746366) |  | 3363911(3133386, 3594435) | 1489999(1373089, 1606908) | 4830880(4483436, 5178323) |
| 2039 | 12145238(10817519, 13472957) | 5997967(4552908, 7443025) | 18191408(20845196, 20845196) |  | 22803339(21218715, 24387963) | 10475080(9645815, 11304345) | 33137035(30725560, 35548509) |  | 3336152(3098557, 3573747) | 1477592(1357061, 1598123) | 4790994(4432849, 5149139) |
| 2040 | 11983955(10618902, 13349009) | 5927163(4443473, 7410853) | 17955855(20681044, 20681044) |  | 22605901(20976062, 24235740) | 10386022(9532613, 11239432) | 32852558(30371670, 35333446) |  | 3306764(3062419, 3551108) | 1464546(1340545, 1588547) | 4748850(4380476, 5117223) |
| 2041 | 11818255(10416897, 13219614) | 5854648(4334220, 7375077) | 17714274(20507839, 20507839) |  | 22398274(20725122, 24071426) | 10294104(9417372, 11170835) | 32555090(30007534, 35102646) |  | 3275900(3025094, 3526706) | 1451069(1323721, 1578417) | 4704756(4326572, 5082940) |
| 2042 | 11649024(10212146, 13085902) | 5780555(4225216, 7335894) | 17467767(20327006, 20327006) |  | 22178083(20463361, 23892806) | 10197100(9297908, 11096293) | 32239522(29627969, 34851074) |  | 3243331(2986319, 3500342) | 1436912(1306343, 1567482) | 4658202(4270600, 5045804) |
| 2043 | 11477369(10005672, 12949066) | 5705195(4116708, 7293683) | 17217667(20140030, 20140030) |  | 21953648(20198784, 23708511) | 10097439(9176558, 11018320) | 31917182(29243823, 34590542) |  | 3210116(2947116, 3473116) | 1422402(1288723, 1556081) | 4610728(4214028, 5007427) |
| 2044 | 11302318(9796594, 12808043) | 5627739(4008100, 7247378) | 16961739(19944319, 19944319) |  | 21724522(19931036, 23518008) | 9994373(9052680, 10936065) | 31586315(28853571, 34319059) |  | 3176040(2907292, 3444787) | 1407382(1270724, 1544039) | 4561859(4156436, 4967281) |
| 2045 | 11123524(9584538, 12662511) | 5548306(3899498, 7197115) | 16700059(19739994, 19739994) |  | 21486657(19656320, 23316995) | 9887821(8926191, 10849450) | 31243181(28453719, 34032643) |  | 3140542(2866326, 3414757) | 1391829(1252326, 1531331) | 4511027(4097295, 4924758) |
| 2046 | 10941799(9370141, 12513456) | 5467810(3791563, 7144057) | 16434531(19529329, 19529329) |  | 21239280(19373751, 23104809) | 9778517(8797690, 10759343) | 30887641(28043912, 33731370) |  | 3103649(2824218, 3383079) | 1375858(1233623, 1518093) | 4458327(4036662, 4879993) |
| 2047 | 10757930(9154007, 12361853) | 5386319(3684327, 7088311) | 16166063(19313491, 19313491) |  | 20980717(19081473, 22879960) | 9664583(8665349, 10663817) | 30515858(27620210, 33411505) |  | 3065270(2780840, 3349700) | 1359286(1214430, 1504141) | 4403466(3974207, 4832726) |
| 2048 | 10573019(8937166, 12208872) | 5304282(3578124, 7030441) | 15896191(19094206, 19094206) |  | 20719691(18787668, 22651713) | 9549025(8531969, 10566081) | 30140235(27194180, 33086290) |  | 3026515(2737227, 3315802) | 1342505(1195114, 1489896) | 4348128(3911492, 4784764) |
| 2049 | 10386419(8719073, 12053765) | 5220867(3472389, 6969346) | 15623140(18869355, 18869355) |  | 20455702(18491871, 22419533) | 9431130(8396931, 10465328) | 29758954(26764188, 32753720) |  | 2987182(2693198, 3281167) | 1325374(1175550, 1475197) | 4291841(3848094, 4735588) |
| 2050 | 10197817(8499435, 11896199) | 5136189(3367219, 6905160) | 15346921(18638953, 18638953) |  | 20184881(18190447, 22179314) | 9310633(8259978, 10361288) | 29368232(26326688, 32409776) |  | 2946712(2648227, 3245196) | 1307828(1155679, 1459977) | 4234011(3783458, 4684563) |
| YLDs, years lived with disability; TBI, traumatic brain injury; UI, uncertainty interval | | | | | | | | | | | |
