# Supplementary material for: Global burden of traumatic brain injury from 1990 to 2021 and projections to 2050: A GBD 2021–based study using interpretable machine learning
Source: Medicine (Baltimore). 2026 Jul 24;105(30):e49918. doi: 10.1097/MD.0000000000049918 (PMC13406132; doi:10.1097/MD.0000000000049918)
Supplement: Supplementary file 6 [file medi-105-e49918-s006.docx]

**Supplementary Table S2.** The prevalence cases and age-standardized prevalence rate of traumatic brain injury in 1990 and 2021, and its temporal trends from 1990 to 2021 ASR Age-standardized rate

| Characteristics | 1990 | | |  | 2021 | | | 1990-2021 |
| --- | --- | --- | --- | --- | --- | --- | --- | --- |
|  | Prevalence cases No.x10'5 (95% UI) | ASR per 100,000 No. (95% UI) | Male/Female |  | Prevalence cases No.x10'5 (95% UI) | ASR per 100,000 No. (95% UI) | Male/Female | Percentage change in age-standardised rates |
| Global | 24745606 (23861550, 25847393) | 536.72(517.44, 560.46) | 2.15 |  | 37928494 (36333777, 39771327) | 448.04(429.33, 469.75) | 2.17 | -16.52%(-17.62, -15.25) |
| High SDl | 5418484 (5191537, 5656586) | 539.65(517.84, 563.45) | 2.04 |  | 6755018 (6460860, 7077437) | 425.88(407.03, 445.42) | 1.92 | -21.08%(-22.20, -19.93) |
| High-middle SDl | 7452799 (7173616, 7780227) | 702.29(676.46, 733.40) | 2.38 |  | 9420449 (9028814, 9842899) | 543.29(520.89, 567.07) | 2.27 | -22.64%(-23.58, -21.64) |
| Middle SDl | 7185406 (6896458, 7515548) | 514.87(494.59, 539.23) | 2.23 |  | 12596395 (12025453, 13235723) | 464.06(442.99, 487.30) | 2.31 | -9.87%(-11.25, -8.34) |
| Low-middle SDl | 3491497 (3337414, 3667389) | 415.74(398.59, 435.82) | 1.91 |  | 6539292 (6262026, 6849086) | 391.62(374.42, 410.83) | 2.00 | -5.80 %(-7.05, -4.50) |
| Low SDI | 1163643 (1070925, 1324342) | 340.30(317.91, 376.90) | 2.06 |  | 2578216 (2377837, 2857229) | 344.15(318.95, 376.63) | 2.12 | 1.13 %(-2.15, 4.46) |
| Andean Latin America | 124051 (117812, 131659) | 434.00(415.70, 455.49) | 2.86 |  | 263002 (251379, 274740) | 409.98(392.14, 428.33) | 2.92 | -5.53 %(-7.54, -3.61) |
| Australasia | 142047 (134149, 152284) | 646.99(609.99, 692.99) | 1.85 |  | 210178 (198130, 223338) | 518.13(483.07, 557.01) | 1.67 | -19.92%(-21.96, -17.93) |
| Caribbean | 132354 (127181, 137843) | 439.26(422.80, 457.58) | 2.52 |  | 262836 (243264, 290669) | 514.44(473.08, 572.89) | 1.99 | 17.11 %(9.64, 29.81) |
| Central Asia | 354154 (339544, 368705) | 611.27(587.29, 634.99) | 3.15 |  | 450495 (430153, 470411) | 470.69(450.06, 490.75) | 2.90 | -23.00%(-24.17, -21.79) |
| Central Europe | 1422638 (1362744, 1488637) | 1019.12(976.56, 1066.63) | 2.52 |  | 1276908 (1217810, 1343114) | 771.30(736.95, 811.82) | 2.51 | -24.32%(-25.28, -23.46) |
| Central Latin America | 941286 (896039, 990453) | 779.41(746.98, 815.59) | 3.26 |  | 1489308 (1426868, 1556450) | 569.76(545.79, 595.33) | 3.21 | -26.90%(-27.88, -25.85) |
| Central Sub-Saharan Africa | 117829 (110798, 126707) | 313.56(297.52, 333.44) | 1.84 |  | 290898 (269365, 319018) | 319.29(297.91, 348.67) | 2.22 | 1.83 %(-1.07, 6.06) |
| East Asia | 5164961 (4960266, 5422702) | 474.20(455.28, 498.08) | 1.86 |  | 9523998 (9077425, 10041469) | 477.23(455.96, 501.02) | 2.03 | 0.64 %(-1.03, 2.46) |
| Eastern Europe | 3022502 (2886154, 3170285) | 1169.35(1116.93, 1224.19) | 3.05 |  | 2492282 (2372412, 2618739) | 888.95(846.90, 933.90) | 3.00 | -23.98%(-25.05, -22.92) |
| Eastern Sub-Saharan Africa | 393107 (346421, 460599) | 303.78(277.83, 340.60) | 2.36 |  | 748723 (682271, 849678) | 276.20(252.86, 312.48) | 2.58 | -9.08%(-10.74, -7.54) |
| High-income Asia Pacifc | 927145 (884834, 975842) | 474.20(452.24, 499.66) | 2.04 |  | 922121 (876101, 972923) | 302.88(286.76, 320.54) | 1.84 | -36.13%(-37.39, -34.70) |
| High-income North America | 1576217 (1505955, 1666557) | 495.85(473.79, 524.10) | 1.90 |  | 2052302 (1945820, 2160393) | 383.93(364.90, 402.92) | 1.66 | -22.57%(-24.36, -20.56) |
| North Africa and Middle East | 1727152 (1608149, 1900293) | 680.32(639.23, 740.87) | 2.32 |  | 3447607 (3204256, 3744044) | 593.00(553.40, 641.83) | 2.54 | -12.83%(-15.55, -10.46) |
| Oceania | 15211 (14574, 15995) | 334.46(320.41, 351.33) | 1.69 |  | 43104 (40952, 45459) | 400.84(382.36, 421.07) | 1.51 | 19.85%(17.15, 23.26) |
| South Asia | 2923634 (2787072, 3067028) | 377.76(359.22, 396.20) | 1.58 |  | 6311107 (5994081, 6657380) | 383.23(362.52, 405.62) | 1.61 | 1.45 %(-0.05, 3.13) |
| Southeast Asia | 1576449 (1494938, 1716389) | 437.00(416.53, 466.62) | 2.41 |  | 2572303 (2438853, 2741690) | 360.10(341.87, 382.75) | 2.35 | -17.60%(-19.84, -15.00) |
| Southern Latin America | 201375 (189367, 214814) | 422.27(397.55, 449.74) | 2.48 |  | 317384 (300858, 337514) | 409.67(386.45, 437.84) | 2.26 | -2.98 %(-4.32, -1.75) |
| Southern Sub-Saharan Africa | 262822 (246572, 282051) | 703.15(657.83, 753.29) | 2.75 |  | 324082 (305063, 344922) | 434.53(409.48, 462.04) | 3.51 | -38.20%(-39.26, -37.29) |
| Tropical Latin America | 940304 (897603, 989465) | 768.68(733.85, 807.47) | 3.27 |  | 1647002 (1576925, 1732312) | 643.42(615.58, 676.47) | 3.59 | -16.30%(-18.31, -13.97) |
| Western Europe | 2447795 (2329584, 2567650) | 516.30(490.92, 542.27) | 2.17 |  | 2491552 (2352567, 2640652) | 376.34(353.99, 399.85) | 1.82 | -27.11%(-28.59, -25.53) |
| Western Sub-Saharan Africa | 332570 (319782, 347200) | 260.33(249.50, 271.50) | 2.10 |  | 791303 (756628, 834310) | 256.07(244.79, 269.01) | 2.32 | -1.64 %(-3.10, 0.33) |
