# Supplementary material for: Global burden of traumatic brain injury from 1990 to 2021 and projections to 2050: A GBD 2021–based study using interpretable machine learning
Source: Medicine (Baltimore). 2026 Jul 24;105(30):e49918. doi: 10.1097/MD.0000000000049918 (PMC13406132; doi:10.1097/MD.0000000000049918)
Supplement: Supplementary file 7 [file medi-105-e49918-s007.docx]

**Supplementary Table S3.** The YLDs cases and age-standardized YLDs rate of traumatic brain injury in 1990 and 2021, and its temporal trends from 1990 to 2021 ASR Age-standardized rate

| Characteristics | 1990 | | |  | 2021 | | | 1990-2021 |
| --- | --- | --- | --- | --- | --- | --- | --- | --- |
|  | YLDs cases No.x10'5 (95% UI) | ASR per 100,000 No. (95% UI) | Male/Female |  | YLDs cases No.x10'5 (95% UI) | ASR per 100,000 No. (95% UI) | Male/Female | Percentage change in age-standardised rates |
| Global | 3590358 (2532780, 4830321) | 77.27 (54.52, 103.67) | 2.21 |  | 5480354 (3870216, 7331092) | 64.76 (45.75, 86.69) | 2.24 | -16.19%(-17.39, -14.70) |
| High SDl | 734591 (517021, 985434) | 73.37 (51.66, 98.57) | 2.09 |  | 910871 (642616, 1214374) | 58.24 (40.87, 78.36) | 1.99 | -20.61%(-21.93, -19.35) |
| High-middle SDl | 1087477 (759060, 1476761) | 102.13 (71.31, 138.51) | 2.42 |  | 1366204 (954653, 1854000) | 79.20 (55.27, 107.53) | 2.32 | -22.46%(-23.51, -21.20) |
| Middle SDl | 1075380 (755150, 1450217) | 76.24 (53.58, 102.62) | 2.28 |  | 1855632 (1310201, 2494075) | 68.27 (48.19, 91.92) | 2.37 | -10.45%(-12.07, -8.64) |
| Low-middle SDl | 516289 (363976, 697481) | 60.71 (42.84, 82.18) | 1.96 |  | 961050 (681936, 1287923) | 57.06 (40.50, 76.59) | 2.07 | -6.00 %(-7.48, -4.33) |
| Low SDI | 171688 (123473, 226278) | 49.52 (35.66, 65.84) | 2.13 |  | 380918 (275738, 495522) | 50.08 (36.47, 65.35) | 2.20 | 1.14 %(-2.32, 5.03) |
| Andean Latin America | 18514 (13323, 24554) | 64.17 (45.75, 85.79) | 2.92 |  | 38823 (27406, 52570) | 60.33 (42.66, 81.50) | 2.99 | -5.99 %(-9.43, -2.76) |
| Australasia | 18564 (13027, 24738) | 84.59 (59.16, 112.34) | 1.88 |  | 27273 (19167, 36326) | 67.70 (47.56, 90.74) | 1.71 | -19.96%(-23.34, -16.60) |
| Caribbean | 19682 (13629, 26791) | 64.98 (45.15, 88.08) | 2.58 |  | 38527 (27780, 49939) | 75.56 (54.43, 97.83) | 2.03 | 16.28 %(8.47, 29.79) |
| Central Asia | 52750 (36740, 71853) | 90.53 (63.12, 123.12) | 3.22 |  | 66723 (46504, 90670) | 69.43 (48.49, 94.20) | 2.96 | -23.31%(-25.05, -21.41) |
| Central Europe | 207751 (145133, 282814) | 149.14 (103.94, 203.23) | 2.56 |  | 184635 (129916, 250590) | 112.88 (78.80, 153.71) | 2.55 | -24.31%(-25.50, -23.07) |
| Central Latin America | 139783 (99091, 188123) | 114.42 (81.22, 153.71) | 3.34 |  | 219154 (153910, 296100) | 83.70 (58.82, 113.08) | 3.29 | -26.85%(-28.13, -25.57) |
| Central Sub-Saharan Africa | 17462 (12450, 22781) | 45.76 (32.52, 60.29) | 1.87 |  | 43145 (30984, 56807) | 46.64 (33.45, 60.89) | 2.27 | 1.93 %(-2.96, 7.39) |
| East Asia | 778947 (543920, 1061344) | 70.93 (49.53, 96.68) | 1.90 |  | 1409201 (987681, 1922142) | 70.89 (49.38, 96.59) | 2.08 | -0.05 %(-2.01, 2.12) |
| Eastern Europe | 442906 (310063, 604346) | 171.93 (120.43, 234.96) | 3.09 |  | 361905 (253832, 493679) | 130.33 (91.33, 178.34) | 3.05 | -24.20%(-25.56, -22.88) |
| Eastern Sub-Saharan Africa | 58432 (41762, 76852) | 44.50 (32.28, 58.17) | 2.44 |  | 110855 (80127, 144604) | 40.25 (29.33, 52.25) | 2.66 | -9.54%(-11.67, -7.41) |
| High-income Asia Pacifc | 123661 (86852, 167092) | 63.19 (44.50, 85.42) | 2.08 |  | 121024 (85487, 160286) | 40.38 (28.42, 54.28) | 1.88 | -36.09%(-37.76, -34.28) |
| High-income North America | 208244 (145442, 279859) | 65.73 (45.94, 88.44) | 1.95 |  | 265812 (187933, 353325) | 50.29 (35.33, 66.63) | 1.71 | -23.49%(-25.51, -21.11) |
| North Africa and Middle East | 258449 (186082, 338720) | 100.69 (72.47, 132.66) | 2.36 |  | 509844 (367555, 666956) | 87.01 (62.91, 113.60) | 2.62 | -13.59%(-16.20, -10.90) |
| Oceania | 2284 (1575, 3122) | 49.45 (34.27, 67.70) | 1.73 |  | 6452 (4603, 8834) | 59.12 (42.04, 80.98) | 1.56 | 19.55%(14.73, 25.80) |
| South Asia | 429195 (302745, 588397) | 54.66 (38.69, 74.74) | 1.64 |  | 920914 (652975, 1243160) | 55.43 (39.41, 74.82) | 1.67 | 1.42 %(-0.51, 3.62) |
| Southeast Asia | 236489 (166654, 315545) | 64.79 (45.67, 86.65) | 2.46 |  | 381660 (273314, 505887) | 53.15 (38.01, 70.56) | 2.40 | -17.97%(-20.34, -14.94) |
| Southern Latin America | 26522 (18543, 35588) | 55.56 (38.79, 74.45) | 2.53 |  | 41628 (29466, 55531) | 53.86 (38.16, 72.07) | 2.33 | -3.06 %(-6.32, 0.40) |
| Southern Sub-Saharan Africa | 39340 (27142, 53316) | 104.09 (72.10, 141.22) | 2.81 |  | 47961 (33098, 65222) | 63.78 (44.04, 86.44) | 3.61 | -38.72%(-40.13, -37.33) |
| Tropical Latin America | 138950 (97456, 190267) | 112.45 (78.98, 153.77) | 3.35 |  | 240869 (169319, 325629) | 94.12 (66.15, 127.40) | 3.69 | -16.30%(-18.59, -13.65) |
| Western Europe | 323095 (228063, 433656) | 68.54 (48.51, 92.25) | 2.22 |  | 325842 (229154, 437166) | 49.89 (35.11, 67.59) | 1.88 | -27.22%(-28.89, -25.26) |
| Western Sub-Saharan Africa | 49336 (34517, 67131) | 38.15 (26.78, 51.92) | 2.16 |  | 118108 (82770, 159110) | 37.64 (26.60, 50.71) | 2.40 | -1.34 %(-3.30, 1.09) |
