# Supplementary material for: Global burden of traumatic brain injury from 1990 to 2021 and projections to 2050: A GBD 2021–based study using interpretable machine learning
Source: Medicine (Baltimore). 2026 Jul 24;105(30):e49918. doi: 10.1097/MD.0000000000049918 (PMC13406132; doi:10.1097/MD.0000000000049918)
Supplement: Supplementary file 8 [file medi-105-e49918-s008.docx]

**Supplementary Table S4.** Incidence, prevalence, and YLDs for traumatic brain injury for all ages in 1990 and 2021, and percentage change in age-standardised rates from 1990 to 2021 for 204 countries and regions

| Nation (or region) | Incidence | | | | | Prevalence | | | | | YLDs | | | | |
| --- | --- | --- | --- | --- | --- | --- | --- | --- | --- | --- | --- | --- | --- | --- | --- |
|  | 1990 counts | 1990 age-standardised rates (per 100 000) | 2021 counts | 2021 age-standardised rates (per 100 000) | Percentage change in age-standardised rates, 1990–2021 | 1990 counts | 1990 age-standardised rates (per 100 000) | 2021 counts | 2021 age-standardised rates (per 100 000) | Percentage change in age-standardised rates, 1990–2021 | 1990 counts | 1990 age-standardised rates (per 100 000) | 2021 counts | 2021 age-standardised rates (per 100 000) | Percentage change in age-standardised rates, 1990–2021 |
| American Samoa | 87 (78, 98) | 195.56 (174.63, 217.75) | 85 (75, 96) | 179.18 (156.98, 201.13) | -8.38% (-11.78, -5.32) | 116 (110, 121) | 338.15 (320.05, 352.77) | 166 (156, 177) | 330.10 (310.39, 352.65) | -2.38% (-6.40, 3.51) | 17 (12, 24) | 50.11 (34.65, 68.73) | 24 (18, 33) | 48.34 (34.78, 64.68) | -3.54% (-9.85, 5.39) |
| Antigua and Barbuda | 134 (115, 161) | 217.65 (187.47, 259.72) | 193 (163, 232) | 220.10 (184.06, 265.74) | 1.13% (-3.62, 6.35) | 191 (183, 200) | 347.59 (333.32, 364.17) | 361 (346, 380) | 346.28 (330.97, 364.50) | -0.38% (-2.54, 2.47) | 28 (20, 38) | 51.18 (35.63, 68.99) | 53 (37, 72) | 50.57 (35.45, 68.76) | -1.19% (-6.30, 4.92) |
| Arab Republic of Egypt | 196368 (172179, 226731) | 353.77 (310.40, 402.83) | 273843 (240114, 309032) | 273.96 (240.61, 307.66) | -22.56% (-25.60, -19.51) | 266229 (253097, 282403) | 629.14 (599.70, 667.38) | 432497 (411994, 465067) | 497.20 (474.25, 533.07) | -20.97% (-23.04, -18.62) | 40177 (27398, 54193) | 93.75 (64.27, 127.17) | 64698 (44969, 86055) | 73.51 (51.36, 97.82) | -21.58% (-25.21, -17.53) |
| Argentine Republic | 125488 (95205, 172217) | 376.91 (287.39, 514.56) | 153943 (120191, 206676) | 346.27 (268.45, 467.93) | -8.13% (-11.21, -4.63) | 132576 (124394, 141723) | 410.49 (385.71, 438.80) | 199466 (188778, 212379) | 393.20 (371.20, 420.12) | -4.21% (-5.75, -2.68) | 17445 (12149, 23398) | 54.00 (37.59, 72.35) | 26204 (18593, 35031) | 51.75 (36.66, 69.27) | -4.17% (-8.73, 0.41) |
| Australia | 91127 (69611, 121899) | 562.01 (422.54, 764.42) | 113190 (84885, 148219) | 464.61 (342.72, 637.46) | -17.33% (-21.84, -12.52) | 113127 (106576, 121404) | 618.31 (582.15, 663.30) | 172062 (162169, 182803) | 504.04 (469.86, 542.06) | -18.48% (-20.64, -16.17) | 14789 (10357, 19707) | 80.85 (56.40, 107.60) | 22317 (15722, 29808) | 65.85 (46.28, 88.23) | -18.56% (-22.47, -14.72) |
| Barbados | 473 (414, 541) | 180.89 (158.30, 206.91) | 550 (474, 623) | 181.17 (157.16, 207.56) | 0.15% (-2.98, 3.65) | 797 (765, 834) | 304.66 (292.31, 318.74) | 1206 (1161, 1262) | 302.00 (290.44, 317.08) | -0.87% (-2.71, 0.87) | 118 (82, 160) | 45.20 (31.61, 61.46) | 176 (123, 238) | 44.48 (31.11, 60.76) | -1.60% (-7.36, 4.61) |
| Belize | 468 (409, 539) | 242.32 (213.15, 275.85) | 1239 (1093, 1402) | 280.53 (247.55, 317.13) | 15.77% (10.49, 21.03) | 531 (510, 557) | 413.24 (397.75, 431.95) | 1948 (1875, 2039) | 507.70 (489.18, 530.00) | 22.86% (20.10, 25.82) | 80 (56, 108) | 61.47 (43.07, 83.90) | 291 (203, 393) | 75.13 (52.77, 101.10) | 22.22% (16.47, 28.59) |
| Bermuda | 127 (111, 144) | 211.92 (185.03, 241.39) | 131 (110, 151) | 187.08 (159.87, 218.38) | -11.72% (-15.96, -7.54) | 229 (219, 238) | 347.81 (333.34, 362.60) | 300 (286, 313) | 308.69 (295.09, 323.86) | -11.25% (-13.58, -8.84) | 34 (23, 46) | 51.76 (35.59, 69.71) | 44 (31, 59) | 45.47 (31.73, 62.43) | -12.15% (-17.45, -6.58) |
| Bolivarian Republic of Venezuela | 73517 (63979, 83997) | 384.25 (337.86, 435.19) | 92110 (81321, 103889) | 356.12 (313.57, 402.92) | -7.32% (-10.48, -3.35) | 91910 (88282, 95998) | 639.03 (615.15, 665.50) | 188059 (180731, 196499) | 639.97 (614.14, 668.53) | 0.15% (-2.31, 2.87) | 13723 (9554, 18889) | 94.45 (66.02, 129.23) | 27738 (19342, 37879) | 94.51 (65.99, 129.00) | 0.07% (-4.24, 4.82) |
| Bosnia and Herzegovina | 25173 (20926, 30597) | 557.14 (464.40, 676.00) | 12891 (10894, 15000) | 394.81 (330.52, 468.32) | -29.14% (-35.12, -23.52) | 37332 (35409, 39651) | 802.11 (760.62, 851.89) | 32356 (29836, 35138) | 711.01 (654.71, 775.93) | -11.36% (-16.33, -5.01) | 5475 (3845, 7386) | 117.17 (82.47, 157.83) | 4658 (3377, 6025) | 103.45 (74.79, 134.45) | -11.71% (-16.76, -4.15) |
| Brunei Darussalam | 1175 (964, 1476) | 433.10 (358.92, 534.95) | 1462 (1201, 1828) | 317.60 (259.50, 399.79) | -26.67% (-30.49, -22.79) | 1085 (1028, 1151) | 570.00 (545.93, 598.83) | 1806 (1723, 1905) | 399.54 (381.86, 419.79) | -29.91% (-31.71, -28.29) | 146 (100, 198) | 75.56 (51.64, 101.89) | 242 (168, 325) | 52.79 (36.75, 70.58) | -30.13% (-33.52, -26.36) |
| Burkina Faso | 15496 (13722, 17440) | 184.69 (165.62, 205.10) | 40319 (35753, 46294) | 202.40 (180.48, 226.92) | 9.59% (4.30, 18.00) | 15984 (15379, 16661) | 267.70 (257.69, 279.23) | 40905 (39330, 42880) | 289.33 (278.03, 301.97) | 8.08% (6.27, 10.04) | 2363 (1628, 3192) | 39.06 (27.00, 52.47) | 6123 (4297, 8265) | 42.71 (30.04, 58.17) | 9.35% (3.91, 15.44) |
| Canada | 84259 (69537, 101770) | 304.00 (251.22, 369.59) | 99747 (78226, 123012) | 229.90 (184.10, 279.69) | -24.38% (-29.29, -19.06) | 125678 (119686, 132140) | 406.94 (387.50, 428.32) | 177962 (168180, 188033) | 306.12 (288.78, 322.23) | -24.78% (-27.01, -22.29) | 16742 (11713, 22485) | 54.25 (37.80, 72.97) | 23304 (16463, 30852) | 40.48 (28.31, 53.81) | -25.39% (-29.86, -21.10) |
| Central African Republic | 5105 (4543, 5718) | 202.71 (180.82, 225.99) | 15417 (13200, 18861) | 282.73 (247.08, 335.89) | 39.47% (24.33, 62.64) | 5555 (5325, 5857) | 297.04 (284.87, 313.09) | 15323 (14098, 16943) | 373.06 (348.93, 405.26) | 25.59% (18.46, 35.58) | 824 (569, 1114) | 43.33 (30.16, 58.88) | 2283 (1633, 3002) | 54.76 (39.28, 72.37) | 26.37% (16.58, 39.40) |
| Commonwealth of Dominica | 142 (125, 161) | 194.43 (171.74, 219.44) | 135 (119, 152) | 198.80 (175.75, 224.02) | 2.25% (-0.66, 5.23) | 202 (194, 212) | 326.22 (313.65, 342.03) | 281 (268, 294) | 363.63 (346.11, 382.78) | 11.47% (8.02, 15.97) | 30 (21, 41) | 48.50 (33.64, 66.11) | 41 (29, 55) | 53.54 (37.86, 71.70) | 10.38% (3.89, 18.21) |
| Commonwealth of the Bahamas | 608 (543, 682) | 233.52 (208.87, 260.72) | 925 (823, 1035) | 235.31 (208.48, 264.50) | 0.77% (-2.58, 4.29) | 865 (830, 910) | 406.15 (390.09, 427.33) | 1859 (1779, 1943) | 432.48 (413.72, 453.10) | 6.48% (3.16, 10.27) | 130 (89, 178) | 60.42 (41.81, 82.62) | 276 (195, 377) | 64.03 (45.46, 87.32) | 5.98% (0.34, 12.39) |
| Cook Islands | 42 (37, 48) | 232.66 (204.62, 261.77) | 33 (29, 38) | 175.89 (152.41, 200.69) | -24.40% (-31.17, -18.55) | 60 (57, 63) | 383.94 (366.72, 406.35) | 74 (71, 78) | 338.49 (320.55, 357.87) | -11.84% (-14.51, -8.62) | 9 (6, 12) | 57.24 (39.76, 78.04) | 11 (8, 14) | 49.71 (35.60, 66.64) | -13.16% (-18.42, -6.76) |
| Czech Republic | 83357 (69295, 97246) | 764.77 (640.52, 894.29) | 62239 (51437, 73214) | 512.65 (431.23, 603.33) | -32.97% (-35.04, -30.46) | 134503 (127764, 141419) | 1108.23 (1055.23, 1165.92) | 127501 (120994, 134634) | 811.61 (772.22, 853.81) | -26.77% (-28.18, -25.28) | 19552 (13695, 26587) | 161.82 (112.86, 222.01) | 18332 (13025, 24713) | 118.44 (83.48, 161.33) | -26.81% (-29.56, -24.11) |
| Democratic People's Republic of Korea | 44683 (39614, 49962) | 215.28 (190.89, 240.99) | 58042 (51289, 64556) | 199.30 (176.61, 221.82) | -7.42% (-11.12, -3.90) | 79474 (76357, 84495) | 407.05 (391.23, 431.47) | 128884 (123542, 135520) | 394.23 (378.13, 414.01) | -3.15% (-4.98, -1.41) | 12051 (8434, 16264) | 61.34 (42.95, 82.72) | 19399 (13511, 26479) | 59.41 (41.45, 80.85) | -3.15% (-7.42, 1.31) |
| Democratic Republic of Sao Tome and Principe | 198 (171, 228) | 167.15 (145.58, 191.34) | 418 (359, 479) | 219.81 (188.97, 249.23) | 31.50% (24.45, 39.59) | 208 (199, 217) | 248.80 (238.82, 260.21) | 483 (461, 507) | 309.81 (295.84, 324.32) | 24.52% (21.36, 28.39) | 31 (22, 42) | 36.70 (25.61, 49.99) | 72 (50, 99) | 45.80 (31.91, 62.42) | 24.80% (17.17, 31.83) |
| Democratic Republic of the Congo | 67208 (59250, 76062) | 185.10 (164.15, 207.25) | 148651 (132159, 168365) | 183.19 (162.80, 205.31) | -1.03% (-4.85, 4.02) | 73576 (70559, 77725) | 291.39 (278.89, 308.55) | 187243 (174974, 202759) | 309.49 (291.28, 331.98) | 6.21% (1.75, 13.20) | 10905 (7564, 14544) | 42.45 (29.81, 56.76) | 27795 (19759, 36690) | 45.27 (32.48, 59.88) | 6.64% (-1.13, 15.49) |
| Democratic Republic of Timor-Leste | 2848 (2137, 4033) | 348.14 (267.11, 481.23) | 2175 (1901, 2476) | 164.25 (144.25, 184.93) | -52.82% (-65.33, -38.14) | 3442 (2493, 4800) | 532.10 (412.19, 702.44) | 4812 (3712, 6328) | 481.37 (365.59, 640.18) | -9.53% (-12.04, -7.09) | 509 (374, 695) | 78.03 (57.99, 102.67) | 701 (532, 909) | 69.60 (52.92, 90.97) | -10.81% (-15.53, -5.83) |
| Democratic Socialist Republic of Sri Lanka | 86254 (66207, 117921) | 480.03 (377.33, 643.32) | 55768 (47448, 64071) | 239.18 (203.93, 274.30) | -50.17% (-62.80, -35.15) | 72767 (66584, 81698) | 489.95 (455.29, 536.05) | 138402 (120937, 161052) | 548.67 (477.14, 643.03) | 11.98% (2.61, 23.31) | 10933 (7833, 14515) | 72.88 (52.33, 96.69) | 20134 (15251, 25223) | 79.93 (60.62, 99.90) | 9.68% (-0.31, 22.21) |
| Dominican Republic | 14890 (13205, 16860) | 206.43 (184.54, 232.23) | 28358 (25239, 31772) | 253.01 (224.99, 283.88) | 22.56% (19.15, 25.91) | 19793 (18916, 20864) | 374.83 (358.94, 394.17) | 49139 (47090, 51536) | 451.70 (433.00, 473.63) | 20.51% (18.15, 22.81) | 2971 (2057, 4053) | 55.71 (38.81, 76.27) | 7291 (5107, 9854) | 66.82 (46.84, 90.32) | 19.95% (13.92, 25.62) |
| Eastern Republic of Uruguay | 14877 (10691, 21506) | 484.69 (346.74, 703.66) | 13410 (10239, 18345) | 408.69 (308.23, 571.13) | -15.68% (-23.07, -8.07) | 17016 (15848, 18354) | 501.79 (466.58, 543.35) | 19365 (18362, 20576) | 456.42 (429.02, 490.60) | -9.04% (-11.52, -6.42) | 2217 (1536, 2999) | 65.57 (45.30, 89.00) | 2525 (1793, 3401) | 59.99 (42.23, 80.99) | -8.51% (-13.31, -3.69) |
| Federal Democratic Republic of Ethiopia | 271485 (195379, 391467) | 527.95 (398.57, 722.19) | 184685 (149992, 245267) | 188.17 (157.27, 233.33) | -64.36% (-71.76, -55.26) | 130572 (116736, 148588) | 378.42 (351.06, 414.52) | 185943 (169230, 226970) | 272.26 (249.23, 322.04) | -28.05% (-31.75, -20.51) | 19606 (13588, 26216) | 55.71 (39.14, 73.92) | 27525 (19754, 37063) | 39.62 (28.66, 53.29) | -28.90% (-33.13, -20.73) |
| Federal Democratic Republic of Nepal | 36901 (31401, 43349) | 209.62 (176.62, 245.59) | 65317 (54177, 76692) | 220.61 (181.94, 260.54) | 5.24% (0.95, 9.55) | 41168 (39054, 43572) | 307.74 (292.71, 324.00) | 99736 (91512, 108773) | 367.80 (339.51, 398.27) | 19.51% (13.97, 27.02) | 6015 (4251, 8295) | 44.37 (31.47, 60.38) | 14519 (10516, 19091) | 53.15 (38.71, 69.68) | 19.78% (10.63, 29.35) |
| Federal Republic of Germany | 293773 (236332, 361605) | 377.81 (304.37, 468.54) | 255808 (199130, 326475) | 294.65 (228.67, 375.18) | -22.01% (-27.05, -17.01) | 487969 (464694, 513842) | 477.37 (453.64, 504.30) | 487321 (458715, 519136) | 363.41 (341.09, 387.29) | -23.87% (-26.33, -21.50) | 64519 (45451, 86239) | 63.50 (44.71, 85.22) | 63554 (45078, 84804) | 48.11 (33.79, 65.31) | -24.24% (-28.33, -19.78) |
| Federal Republic of Nigeria | 139542 (122299, 158539) | 166.20 (146.76, 187.63) | 308475 (272807, 349316) | 154.98 (136.76, 174.10) | -6.75% (-10.00, -3.06) | 166848 (159115, 174757) | 270.22 (256.15, 283.16) | 359805 (342789, 379729) | 248.85 (236.91, 261.85) | -7.91% (-9.41, -6.09) | 24711 (17307, 34004) | 39.58 (27.80, 54.14) | 53701 (37670, 73002) | 36.55 (25.86, 49.65) | -7.67% (-9.84, -5.05) |
| Federal Republic of Somalia | 22955 (18017, 31489) | 298.27 (240.10, 390.64) | 43527 (37992, 51917) | 223.50 (198.56, 256.27) | -25.07% (-36.08, -14.01) | 15822 (13336, 25819) | 303.66 (268.79, 437.66) | 45599 (40111, 54008) | 341.69 (306.05, 409.75) | 12.52% (-8.39, 25.51) | 2369 (1603, 3807) | 44.76 (31.30, 65.17) | 6780 (4937, 8836) | 49.92 (36.57, 65.57) | 11.51% (-10.17, 25.96) |
| Federated States of Micronesia | 202 (180, 225) | 221.88 (198.25, 246.46) | 220 (197, 244) | 222.72 (199.24, 247.52) | 0.38% (-3.11, 3.93) | 257 (245, 271) | 373.66 (355.26, 394.09) | 354 (337, 374) | 381.02 (362.56, 401.66) | 1.97% (-0.29, 4.22) | 39 (27, 53) | 55.50 (38.72, 75.69) | 53 (37, 73) | 56.43 (39.03, 77.77) | 1.67% (-2.77, 7.03) |
| Federative Republic of Brazil | 650439 (558901, 757785) | 445.07 (385.10, 514.95) | 806412 (703840, 925786) | 353.32 (306.70, 405.45) | -20.61% (-22.90, -18.47) | 926754 (884571, 974967) | 776.28 (741.12, 815.21) | 1613283 (1544456, 1696907) | 647.23 (619.21, 680.92) | -16.62% (-18.64, -14.30) | 136944 (96106, 187542) | 113.55 (79.80, 155.28) | 235895 (165777, 318897) | 94.68 (66.53, 128.11) | -16.62% (-18.91, -13.98) |
| French Republic | 275064 (222874, 337608) | 469.13 (377.80, 577.45) | 247115 (192492, 313529) | 351.54 (272.89, 446.58) | -25.06% (-29.83, -20.30) | 421722 (400662, 442816) | 604.83 (574.38, 635.56) | 450831 (424887, 478650) | 446.29 (421.89, 473.81) | -26.21% (-28.33, -23.86) | 55526 (38683, 74797) | 80.13 (55.65, 108.09) | 58806 (41161, 78832) | 59.12 (41.30, 80.44) | -26.21% (-29.80, -22.44) |
| Gabonese Republic | 1746 (1551, 1952) | 195.23 (173.34, 218.52) | 2876 (2557, 3217) | 177.23 (157.24, 197.98) | -9.22% (-11.77, -6.71) | 2181 (2095, 2288) | 306.41 (294.26, 321.00) | 4027 (3872, 4230) | 286.54 (275.10, 301.10) | -6.48% (-7.89, -4.89) | 323 (223, 441) | 45.06 (31.24, 61.52) | 599 (414, 813) | 42.10 (29.09, 57.19) | -6.58% (-12.06, -0.86) |
| Georgia | 22202 (19087, 25444) | 402.51 (346.36, 463.06) | 15037 (13054, 17235) | 426.66 (368.72, 491.30) | 6.00% (2.08, 9.64) | 38135 (36406, 39897) | 640.01 (611.01, 670.55) | 31858 (30366, 33447) | 700.22 (667.48, 734.77) | 9.41% (7.03, 11.77) | 5640 (3920, 7713) | 94.81 (65.98, 129.90) | 4646 (3303, 6346) | 103.05 (72.98, 141.69) | 8.69% (3.82, 13.77) |
| Grand Duchy of Luxembourg | 1779 (1467, 2190) | 474.04 (389.02, 584.71) | 2159 (1710, 2742) | 342.62 (266.48, 438.70) | -27.72% (-32.91, -22.43) | 2855 (2717, 3001) | 606.31 (576.08, 637.62) | 3758 (3549, 3984) | 430.54 (405.12, 457.03) | -28.99% (-31.10, -26.67) | 378 (267, 514) | 80.56 (56.59, 109.52) | 497 (346, 666) | 57.33 (39.81, 77.42) | -28.84% (-32.43, -24.88) |
| Greenland | 230 (189, 279) | 413.44 (338.91, 499.48) | 181 (145, 226) | 306.62 (246.07, 380.51) | -25.84% (-29.59, -22.01) | 246 (234, 258) | 525.46 (499.89, 549.79) | 270 (255, 284) | 404.53 (382.80, 426.65) | -23.01% (-24.54, -21.57) | 33 (23, 44) | 69.06 (48.23, 93.01) | 36 (25, 48) | 53.36 (36.87, 72.22) | -22.73% (-26.50, -18.99) |
| Grenada | 183 (158, 212) | 212.61 (185.01, 244.53) | 224 (192, 263) | 216.58 (184.43, 254.72) | 1.86% (-2.68, 6.92) | 272 (248, 310) | 378.05 (348.31, 424.22) | 412 (389, 442) | 355.60 (336.39, 379.41) | -5.94% (-11.99, -1.56) | 40 (29, 52) | 55.80 (40.10, 73.18) | 60 (43, 80) | 51.96 (37.22, 69.29) | -6.87% (-14.76, -0.44) |
| Guam | 233 (206, 264) | 174.23 (154.07, 196.10) | 252 (220, 285) | 153.29 (133.98, 172.94) | -12.02% (-14.90, -8.86) | 355 (339, 373) | 311.80 (298.43, 326.62) | 509 (488, 533) | 273.79 (262.41, 286.69) | -12.19% (-14.14, -10.29) | 54 (37, 74) | 46.63 (32.55, 63.69) | 75 (53, 102) | 40.61 (28.39, 55.48) | -12.91% (-18.60, -7.10) |
| Hashemite Kingdom of Jordan | 9510 (8422, 10909) | 259.88 (230.08, 292.14) | 23833 (20750, 27143) | 195.69 (171.06, 221.03) | -24.70% (-27.70, -21.56) | 11062 (10585, 11597) | 464.96 (446.01, 488.42) | 38132 (36463, 39970) | 355.81 (340.24, 372.21) | -23.47% (-25.28, -21.73) | 1668 (1172, 2267) | 68.95 (48.90, 94.23) | 5675 (3923, 7705) | 52.31 (36.39, 70.60) | -24.13% (-28.08, -20.07) |
| Hellenic Republic | 42392 (34760, 52149) | 422.48 (345.39, 523.78) | 27239 (22244, 32993) | 303.15 (243.00, 382.95) | -28.24% (-31.34, -24.66) | 70013 (66672, 73777) | 553.93 (527.01, 585.73) | 58886 (56349, 62089) | 387.21 (367.12, 412.79) | -30.10% (-31.90, -28.02) | 9286 (6528, 12508) | 73.81 (52.04, 100.43) | 7708 (5374, 10322) | 51.43 (35.91, 69.89) | -30.33% (-33.99, -26.57) |
| Hungary | 85882 (70917, 99570) | 755.29 (630.31, 876.19) | 55161 (44846, 65602) | 479.58 (396.52, 563.83) | -36.50% (-38.96, -33.49) | 140952 (134209, 148561) | 1098.77 (1048.92, 1153.45) | 106349 (100668, 112771) | 731.76 (695.31, 772.45) | -33.40% (-34.90, -31.73) | 20438 (14429, 27790) | 160.25 (112.93, 218.50) | 15353 (10824, 20747) | 107.10 (74.91, 145.61) | -33.16% (-35.85, -30.45) |
| Independent State of Papua New Guinea | 7519 (6650, 8446) | 206.56 (182.25, 230.97) | 22819 (20255, 25702) | 246.38 (218.39, 278.83) | 19.28% (14.59, 23.56) | 9698 (9292, 10233) | 347.35 (332.35, 366.38) | 33557 (31829, 35424) | 437.12 (415.70, 459.86) | 25.85% (22.47, 29.95) | 1455 (1002, 1991) | 51.24 (35.56, 70.19) | 5030 (3606, 6916) | 64.38 (45.90, 88.54) | 25.64% (19.34, 34.19) |
| Independent State of Samoa | 340 (301, 387) | 214.99 (190.92, 241.99) | 362 (321, 405) | 187.37 (165.80, 209.08) | -12.85% (-19.25, -7.40) | 389 (371, 409) | 340.15 (325.18, 356.81) | 627 (585, 678) | 363.44 (341.55, 390.01) | 6.85% (2.61, 12.76) | 58 (40, 80) | 50.53 (35.06, 69.00) | 93 (66, 124) | 53.67 (37.76, 71.71) | 6.21% (-0.60, 15.08) |
| Ireland | 12986 (10413, 16350) | 360.82 (288.67, 454.15) | 13354 (10281, 17036) | 286.84 (216.86, 370.10) | -20.50% (-26.59, -14.21) | 16428 (15646, 17413) | 441.39 (420.22, 467.97) | 21900 (20623, 23379) | 348.96 (327.57, 373.68) | -20.94% (-23.49, -18.03) | 2176 (1517, 2950) | 58.60 (40.87, 79.38) | 2890 (2005, 3867) | 46.28 (31.97, 62.47) | -21.02% (-25.75, -16.52) |
| Islamic Republic of Afghanistan | 35806 (31473, 42040) | 365.78 (323.46, 424.46) | 221979 (163359, 322603) | 673.01 (506.45, 940.92) | 83.99% (47.26, 128.78) | 86677 (50589, 177308) | 1125.00 (657.76, 2252.60) | 199337 (146802, 282226) | 968.87 (702.56, 1447.27) | -13.88% (-37.09, 20.15) | 12530 (6847, 23435) | 161.78 (88.15, 297.25) | 29284 (21486, 41300) | 138.75 (99.15, 203.59) | -14.24% (-38.19, 20.93) |
| Islamic Republic of Iran | 332689 (282368, 392604) | 582.09 (496.51, 680.99) | 260809 (227290, 297125) | 304.29 (265.73, 346.87) | -47.72% (-54.33, -42.06) | 388950 (362228, 423946) | 948.48 (888.39, 1017.55) | 566561 (534003, 602094) | 623.69 (589.12, 661.38) | -34.24% (-35.95, -32.56) | 58515 (41777, 76219) | 140.76 (100.42, 183.66) | 83596 (59689, 110791) | 91.53 (65.43, 120.92) | -34.97% (-36.74, -33.12) |
| Islamic Republic of Mauritania | 3763 (3349, 4206) | 204.03 (182.70, 227.63) | 6214 (5513, 6965) | 164.21 (146.05, 183.23) | -19.52% (-22.21, -16.47) | 4462 (4284, 4682) | 328.27 (315.13, 345.02) | 8047 (7740, 8419) | 276.81 (265.01, 289.39) | -15.68% (-17.19, -14.04) | 668 (463, 904) | 48.63 (33.79, 65.79) | 1206 (844, 1644) | 41.02 (28.80, 55.72) | -15.66% (-20.09, -11.15) |
| Islamic Republic of Pakistan | 163096 (143066, 185160) | 161.17 (142.88, 181.60) | 336611 (297330, 376708) | 151.70 (135.00, 169.85) | -5.87% (-8.82, -3.23) | 203496 (193601, 213769) | 269.75 (256.39, 283.28) | 513484 (480130, 554103) | 279.16 (263.35, 298.73) | 3.49% (-0.57, 8.87) | 30238 (21047, 41405) | 39.64 (27.82, 54.34) | 76382 (54353, 100125) | 40.96 (29.19, 53.97) | 3.32% (-2.27, 10.88) |
| Jamaica | 4797 (4108, 5662) | 197.90 (170.73, 233.84) | 5835 (5016, 6741) | 203.46 (174.57, 236.20) | 2.81% (-1.66, 7.84) | 6267 (6019, 6582) | 320.33 (307.53, 335.53) | 9928 (9566, 10353) | 324.66 (312.69, 339.03) | 1.35% (-0.37, 3.12) | 926 (657, 1276) | 47.22 (33.55, 64.74) | 1460 (1057, 1963) | 47.77 (34.55, 64.35) | 1.15% (-4.88, 7.46) |
| Japan | 399569 (324094, 505857) | 327.07 (262.10, 420.11) | 264964 (207640, 330982) | 215.06 (168.63, 283.64) | -34.25% (-37.62, -31.29) | 637903 (607340, 673300) | 417.30 (397.03, 441.93) | 586786 (556933, 621351) | 267.20 (252.42, 284.17) | -35.97% (-37.55, -34.45) | 84929 (59578, 114033) | 55.63 (39.09, 74.84) | 76657 (54561, 101543) | 35.63 (25.30, 47.81) | -35.95% (-37.74, -34.26) |
| Kingdom of Bahrain | 1287 (1136, 1457) | 254.88 (225.30, 285.74) | 3289 (2877, 3751) | 213.52 (187.85, 241.42) | -16.23% (-20.13, -11.92) | 1858 (1757, 1976) | 453.96 (431.73, 480.81) | 6460 (6135, 6849) | 391.60 (373.33, 413.62) | -13.74% (-16.00, -11.25) | 282 (195, 390) | 67.53 (46.80, 92.42) | 965 (666, 1299) | 57.66 (39.92, 77.15) | -14.60% (-19.45, -9.65) |
| Kingdom of Belgium | 43982 (35704, 53909) | 449.52 (363.93, 555.76) | 45295 (35257, 57466) | 370.82 (287.88, 468.05) | -17.51% (-23.07, -12.07) | 72082 (68586, 75917) | 578.19 (549.38, 609.99) | 83438 (78255, 88367) | 476.54 (448.54, 504.06) | -17.58% (-20.07, -14.91) | 9530 (6682, 12781) | 76.88 (54.02, 103.57) | 10899 (7638, 14431) | 63.10 (44.15, 84.65) | -17.92% (-21.94, -13.43) |
| Kingdom of Bhutan | 958 (828, 1102) | 167.38 (145.24, 191.36) | 1513 (1279, 1738) | 214.34 (179.16, 246.98) | 28.05% (18.98, 36.29) | 999 (958, 1044) | 249.81 (239.55, 261.06) | 2174 (2058, 2298) | 314.34 (296.67, 332.58) | 25.83% (21.69, 30.33) | 149 (104, 204) | 36.64 (25.74, 49.42) | 320 (227, 432) | 46.01 (32.56, 62.16) | 25.58% (17.74, 34.49) |
| Kingdom of Cambodia | 23287 (20508, 26647) | 241.50 (213.41, 273.89) | 35925 (31159, 41015) | 227.27 (194.14, 261.08) | -5.89% (-13.86, 1.43) | 40503 (27966, 74567) | 521.73 (389.75, 875.60) | 64312 (55796, 84496) | 429.95 (374.34, 563.38) | -17.59% (-35.66, -2.60) | 5984 (3818, 10399) | 76.48 (52.21, 123.40) | 9473 (6886, 12812) | 62.80 (45.72, 84.56) | -17.88% (-37.07, -2.71) |
| Kingdom of Denmark | 19820 (15868, 24547) | 373.13 (302.60, 463.81) | 15657 (12191, 19994) | 273.60 (208.60, 353.97) | -26.67% (-31.56, -21.64) | 31149 (29447, 32877) | 467.64 (443.07, 494.47) | 27428 (25896, 29334) | 329.50 (310.13, 352.85) | -29.54% (-31.95, -26.78) | 4095 (2877, 5505) | 61.91 (43.12, 83.12) | 3605 (2557, 4839) | 43.78 (31.04, 58.90) | -29.30% (-33.46, -24.75) |
| Kingdom of Eswatini | 1733 (1541, 1944) | 241.97 (216.80, 269.70) | 3045 (2687, 3412) | 265.24 (235.51, 294.55) | 9.62% (3.86, 17.15) | 2020 (1927, 2114) | 413.24 (394.08, 433.35) | 3564 (3399, 3756) | 388.01 (370.41, 408.63) | -6.11% (-8.20, -4.43) | 306 (210, 419) | 61.62 (42.80, 83.93) | 530 (368, 730) | 56.93 (39.53, 77.92) | -7.61% (-12.22, -2.66) |
| Kingdom of Lesotho | 2833 (2523, 3173) | 200.44 (178.84, 222.56) | 5509 (4934, 6148) | 297.63 (267.14, 330.45) | 48.49% (43.01, 53.93) | 3606 (3453, 3778) | 329.35 (315.04, 345.66) | 6876 (6526, 7224) | 440.09 (417.93, 461.25) | 33.62% (30.96, 36.34) | 539 (373, 727) | 48.89 (33.82, 66.29) | 1020 (717, 1390) | 64.51 (45.56, 87.43) | 31.95% (25.16, 39.03) |
| Kingdom of Morocco | 85028 (74731, 97815) | 339.07 (298.42, 386.11) | 100129 (88585, 113508) | 270.48 (238.65, 307.04) | -20.23% (-23.21, -16.89) | 116906 (112012, 123022) | 606.19 (580.03, 638.47) | 177608 (169925, 186786) | 472.28 (451.47, 496.15) | -22.09% (-23.66, -20.50) | 17485 (12179, 24081) | 89.77 (62.64, 122.91) | 26072 (18342, 35443) | 69.07 (48.71, 93.89) | -23.06% (-26.29, -19.39) |
| Kingdom of Norway | 17898 (14187, 22326) | 407.78 (326.62, 518.32) | 16725 (12690, 21465) | 287.11 (221.25, 365.36) | -29.59% (-33.79, -25.31) | 27992 (26510, 29583) | 518.62 (492.77, 546.06) | 27644 (25986, 29472) | 351.59 (330.70, 375.11) | -32.21% (-33.82, -30.33) | 3668 (2590, 4913) | 68.60 (48.70, 92.27) | 3621 (2540, 4868) | 46.55 (32.49, 62.85) | -32.14% (-34.23, -29.87) |
| Kingdom of Saudi Arabia | 82532 (71585, 95339) | 580.46 (504.25, 659.14) | 281085 (241210, 321930) | 680.68 (584.24, 776.81) | 17.27% (12.68, 22.01) | 100216 (94953, 105337) | 991.28 (943.57, 1042.18) | 435361 (409714, 462031) | 1218.24 (1156.03, 1288.15) | 22.90% (19.75, 26.16) | 14959 (10541, 20421) | 145.35 (102.37, 196.56) | 64918 (46215, 89166) | 177.76 (125.98, 241.85) | 22.30% (17.84, 27.07) |
| Kingdom of Spain | 148014 (122552, 179959) | 386.50 (319.98, 470.88) | 115993 (91625, 146220) | 277.64 (212.90, 352.43) | -28.17% (-34.90, -20.36) | 228067 (218071, 240207) | 501.25 (478.61, 528.69) | 235603 (223152, 250439) | 346.74 (325.86, 369.48) | -30.83% (-33.74, -27.56) | 30293 (21279, 40692) | 66.91 (46.99, 89.93) | 30943 (21561, 41269) | 46.07 (31.98, 61.79) | -31.15% (-35.11, -26.66) |
| Kingdom of Sweden | 31403 (24905, 39178) | 368.60 (291.50, 473.32) | 30362 (23312, 38856) | 275.42 (210.89, 355.20) | -25.28% (-29.29, -21.95) | 52374 (49520, 55578) | 459.43 (434.51, 487.57) | 52379 (49197, 56098) | 335.32 (315.50, 358.94) | -27.01% (-28.92, -24.96) | 6900 (4854, 9148) | 61.10 (43.12, 81.68) | 6856 (4872, 9187) | 44.46 (31.52, 59.88) | -27.23% (-30.30, -23.84) |
| Kingdom of Thailand | 191045 (170801, 213488) | 325.05 (290.36, 362.52) | 176302 (153076, 198675) | 245.15 (215.10, 275.46) | -24.58% (-28.64, -20.23) | 301608 (288743, 315883) | 614.47 (589.84, 642.26) | 438925 (421029, 460873) | 470.51 (451.38, 494.78) | -23.43% (-25.40, -21.46) | 45447 (31526, 61835) | 91.55 (63.55, 124.35) | 64710 (45077, 88232) | 69.86 (48.47, 95.07) | -23.70% (-27.04, -19.90) |
| Kingdom of the Netherlands | 41529 (33752, 51139) | 282.37 (227.82, 357.16) | 49149 (37758, 62729) | 255.96 (197.83, 327.27) | -9.35% (-15.14, -3.41) | 62071 (58940, 65757) | 352.38 (334.22, 372.73) | 84129 (79060, 89946) | 315.45 (295.59, 337.08) | -10.48% (-12.94, -7.43) | 8245 (5822, 11118) | 46.94 (33.12, 63.59) | 11007 (7801, 14841) | 41.72 (29.52, 56.55) | -11.13% (-16.50, -6.02) |
| Kingdom of Tonga | 140 (124, 159) | 155.29 (138.95, 173.33) | 115 (101, 132) | 115.11 (101.05, 130.84) | -25.87% (-30.19, -21.49) | 186 (178, 196) | 269.78 (257.08, 283.76) | 179 (172, 187) | 201.60 (193.66, 211.52) | -25.28% (-27.59, -22.85) | 28 (20, 39) | 40.11 (27.97, 55.38) | 26 (18, 35) | 29.67 (20.71, 39.71) | -26.01% (-31.28, -20.68) |
| Kyrgyz Republic | 18137 (16039, 20499) | 404.72 (359.55, 454.06) | 18348 (16119, 20773) | 265.68 (233.75, 300.31) | -34.35% (-36.73, -32.10) | 24018 (23080, 25027) | 666.52 (641.97, 694.15) | 27748 (26517, 29043) | 450.53 (431.25, 471.23) | -32.41% (-33.93, -30.99) | 3580 (2467, 4873) | 98.81 (68.19, 134.59) | 4140 (2881, 5633) | 66.79 (46.67, 90.74) | -32.41% (-35.36, -28.90) |
| Lao People's Democratic Republic | 11295 (9524, 14081) | 280.76 (240.11, 341.76) | 12758 (11408, 14274) | 178.52 (158.57, 199.42) | -36.42% (-47.00, -28.93) | 12085 (11565, 12719) | 406.41 (389.89, 425.79) | 19673 (18893, 20525) | 312.96 (301.24, 326.04) | -22.99% (-24.74, -21.27) | 1822 (1273, 2505) | 60.61 (42.58, 83.56) | 2962 (2059, 4045) | 46.61 (32.48, 63.46) | -23.09% (-27.04, -19.24) |
| Lebanese Republic | 14653 (12340, 18044) | 521.67 (439.39, 633.61) | 11867 (10312, 13579) | 206.30 (180.32, 235.90) | -60.45% (-67.01, -53.82) | 24773 (19034, 38000) | 942.52 (741.91, 1414.96) | 28717 (24127, 38007) | 484.09 (403.51, 649.45) | -48.64% (-54.71, -44.20) | 3640 (2595, 5618) | 137.22 (98.75, 206.51) | 4142 (3056, 5596) | 69.78 (51.41, 95.04) | -49.14% (-55.50, -43.96) |
| Malaysia | 32978 (29227, 37030) | 203.48 (180.31, 228.23) | 62435 (54960, 69584) | 195.39 (171.81, 218.20) | -3.97% (-7.40, -0.40) | 46509 (44612, 48885) | 356.40 (342.32, 372.98) | 106899 (102725, 111927) | 337.43 (324.18, 353.26) | -5.32% (-7.01, -3.51) | 6995 (4905, 9523) | 52.92 (37.41, 71.66) | 15906 (11211, 21497) | 49.88 (35.18, 67.51) | -5.74% (-10.08, -0.89) |
| Mongolia | 7994 (6999, 9080) | 368.61 (325.65, 414.70) | 13220 (11502, 15091) | 391.00 (340.12, 445.83) | 6.07% (1.88, 10.54) | 8967 (8537, 9350) | 581.23 (554.35, 605.11) | 20013 (19194, 20820) | 628.22 (603.61, 652.99) | 8.09% (5.83, 10.62) | 1341 (931, 1841) | 86.10 (59.91, 117.45) | 2970 (2064, 4024) | 92.58 (64.32, 125.11) | 7.52% (2.70, 12.82) |
| Montenegro | 3530 (2996, 4101) | 561.43 (476.00, 653.34) | 2986 (2555, 3463) | 471.43 (401.66, 552.19) | -16.03% (-17.98, -13.90) | 5790 (5538, 6066) | 897.99 (858.13, 940.79) | 6023 (5768, 6310) | 750.42 (718.21, 785.90) | -16.43% (-17.76, -14.93) | 854 (594, 1163) | 132.23 (92.00, 180.31) | 878 (619, 1191) | 110.22 (77.16, 150.10) | -16.65% (-19.55, -13.52) |
| New Zealand | 23157 (17826, 30921) | 685.50 (525.66, 918.23) | 26058 (19898, 34068) | 547.71 (411.87, 735.40) | -20.10% (-25.58, -14.16) | 28920 (27285, 30902) | 791.41 (745.93, 847.36) | 38116 (35990, 40452) | 588.17 (551.93, 630.25) | -25.68% (-28.01, -23.15) | 3775 (2648, 5041) | 103.40 (72.54, 138.31) | 4956 (3467, 6571) | 76.93 (53.85, 103.26) | -25.60% (-28.89, -22.08) |
| North Macedonia | 8554 (7303, 10142) | 429.61 (366.37, 508.55) | 8711 (7382, 10099) | 406.40 (343.11, 477.31) | -5.40% (-11.73, 0.31) | 13629 (12991, 14275) | 670.48 (640.09, 701.26) | 18010 (17208, 18905) | 631.95 (604.02, 664.01) | -5.75% (-7.81, -3.95) | 2005 (1392, 2731) | 98.39 (68.29, 134.17) | 2625 (1815, 3560) | 92.63 (64.02, 126.18) | -5.86% (-9.85, -1.58) |
| Northern Mariana Islands | 116 (101, 131) | 262.20 (231.79, 295.63) | 114 (100, 128) | 240.00 (209.00, 270.77) | -8.47% (-12.81, -4.65) | 166 (158, 174) | 472.05 (451.99, 493.27) | 225 (215, 235) | 412.84 (395.13, 431.22) | -12.54% (-14.20, -10.67) | 25 (17, 34) | 70.18 (48.74, 94.87) | 33 (24, 45) | 60.89 (42.93, 82.83) | -13.24% (-17.46, -8.43) |
| Palestine | 6559 (5438, 8331) | 308.26 (260.73, 381.73) | 12189 (10543, 13998) | 240.09 (209.52, 273.77) | -22.11% (-30.62, -13.94) | 8623 (6908, 11090) | 582.89 (483.27, 727.82) | 24436 (19464, 31580) | 609.65 (489.06, 776.92) | 4.59% (-1.87, 11.22) | 1276 (972, 1643) | 85.51 (64.96, 108.96) | 3567 (2719, 4573) | 88.08 (67.53, 112.42) | 3.01% (-4.76, 11.51) |
| People's Democratic Republic of Algeria | 102004 (89465, 116368) | 407.54 (359.19, 460.93) | 117089 (102189, 131607) | 270.48 (236.58, 303.48) | -33.63% (-36.70, -30.55) | 139562 (132496, 148113) | 786.86 (747.03, 837.11) | 223898 (212543, 236763) | 528.70 (502.79, 557.98) | -32.81% (-34.77, -30.71) | 21030 (14595, 28747) | 117.17 (81.27, 158.46) | 33219 (23298, 44731) | 77.96 (54.72, 105.21) | -33.46% (-36.68, -30.37) |
| People's Republic of Bangladesh | 135655 (118999, 155191) | 122.24 (108.34, 138.21) | 188600 (164279, 218503) | 114.31 (99.89, 131.92) | -6.49% (-11.12, -1.43) | 148509 (140530, 158127) | 192.80 (184.14, 203.52) | 336502 (311923, 368036) | 212.72 (197.92, 231.66) | 10.33% (5.89, 15.87) | 22100 (15708, 29779) | 28.30 (20.00, 38.04) | 49448 (35241, 65032) | 31.12 (22.19, 40.93) | 9.96% (1.36, 19.37) |
| People's Republic of China | 2955574 (2563349, 3423630) | 258.53 (224.46, 295.09) | 4208587 (3574631, 4884216) | 265.59 (226.84, 308.75) | 2.73% (-2.80, 7.75) | 4978622 (4778745, 5227317) | 473.63 (454.53, 497.38) | 9282890 (8842361, 9789406) | 481.66 (460.21, 505.73) | 1.69% (-0.02, 3.56) | 750769 (524411, 1023101) | 70.83 (49.48, 96.57) | 1373267 (962256, 1872386) | 71.53 (49.83, 97.40) | 0.99% (-1.01, 3.25) |
| Plurinational State of Bolivia | 15977 (14133, 18120) | 261.01 (231.09, 293.33) | 25425 (22295, 28897) | 222.16 (194.19, 251.70) | -14.88% (-18.28, -11.31) | 19272 (18538, 20036) | 421.82 (406.06, 439.51) | 38482 (36972, 40077) | 361.35 (347.38, 375.97) | -14.33% (-16.14, -12.44) | 2868 (2023, 3919) | 62.13 (43.82, 84.45) | 5677 (3996, 7728) | 52.98 (37.33, 72.28) | -14.73% (-19.26, -9.87) |
| Portuguese Republic | 47156 (39308, 56752) | 475.89 (394.22, 577.13) | 25395 (20452, 31089) | 245.56 (198.75, 305.60) | -48.40% (-52.07, -44.21) | 73591 (70651, 77095) | 623.07 (596.81, 653.66) | 53574 (50892, 56386) | 320.07 (303.21, 338.48) | -48.63% (-50.46, -46.89) | 9740 (6820, 13083) | 82.80 (57.88, 111.52) | 7029 (5008, 9371) | 42.58 (30.17, 57.50) | -48.57% (-51.40, -45.59) |
| Principality of Andorra | 229 (183, 288) | 420.84 (332.74, 526.31) | 359 (280, 460) | 402.32 (311.96, 512.67) | -4.40% (-8.39, -0.82) | 328 (309, 348) | 549.38 (517.35, 581.61) | 686 (646, 730) | 527.56 (496.50, 560.17) | -3.97% (-6.30, -1.87) | 44 (30, 59) | 73.14 (50.51, 97.65) | 90 (63, 122) | 69.97 (48.61, 94.89) | -4.33% (-8.87, 0.23) |
| Principality of Monaco | 82 (66, 101) | 305.09 (242.08, 389.89) | 93 (74, 118) | 281.77 (218.66, 362.49) | -7.64% (-11.56, -3.70) | 169 (161, 179) | 376.17 (356.44, 400.17) | 202 (192, 215) | 343.72 (323.39, 366.13) | -8.63% (-10.88, -6.03) | 22 (16, 30) | 50.25 (34.88, 67.57) | 27 (19, 35) | 45.70 (32.14, 61.35) | -9.05% (-13.69, -3.94) |
| Puerto Rico | 10033 (8839, 11275) | 276.37 (243.27, 310.93) | 10236 (8684, 11754) | 275.22 (239.05, 313.85) | -0.42% (-4.95, 3.59) | 17545 (16797, 18312) | 485.30 (464.59, 506.37) | 24222 (23039, 25593) | 487.01 (463.67, 515.45) | 0.35% (-2.88, 5.70) | 2600 (1818, 3528) | 71.92 (50.32, 97.52) | 3498 (2479, 4723) | 71.57 (50.45, 96.58) | -0.49% (-5.46, 7.34) |
| Republic of Albania | 19099 (15955, 23182) | 553.87 (464.25, 670.64) | 11908 (10069, 13843) | 462.63 (386.12, 545.65) | -16.47% (-20.84, -12.17) | 25530 (24240, 27007) | 911.21 (868.33, 961.95) | 26084 (24853, 27378) | 767.96 (730.36, 808.98) | -15.72% (-18.01, -13.58) | 3778 (2622, 5124) | 133.83 (93.59, 180.98) | 3809 (2665, 5140) | 113.07 (78.68, 153.94) | -15.51% (-19.20, -11.95) |
| Republic of Angola | 29029 (23898, 37353) | 280.53 (235.70, 351.87) | 48103 (42784, 54046) | 169.98 (151.03, 190.15) | -39.41% (-50.16, -29.36) | 31124 (25932, 38158) | 406.92 (352.00, 480.74) | 68987 (62127, 78206) | 346.50 (310.50, 397.47) | -14.85% (-18.30, -11.66) | 4607 (3447, 5934) | 59.53 (44.64, 76.40) | 10195 (7562, 12996) | 50.34 (37.66, 63.60) | -15.43% (-21.05, -9.31) |
| Republic of Armenia | 14037 (12149, 16082) | 404.62 (350.83, 463.43) | 7020 (6127, 7968) | 237.22 (205.37, 272.34) | -41.37% (-43.68, -39.05) | 26515 (23327, 31715) | 821.30 (724.26, 980.27) | 18109 (16268, 20539) | 499.91 (447.84, 569.66) | -39.13% (-42.29, -34.97) | 3920 (2889, 5084) | 120.72 (89.06, 156.52) | 2654 (1984, 3398) | 73.65 (55.23, 93.74) | -38.99% (-43.31, -33.67) |
| Republic of Austria | 39228 (31861, 48032) | 506.35 (406.93, 630.66) | 30367 (23912, 38247) | 339.60 (264.04, 429.68) | -32.93% (-37.25, -28.50) | 59596 (56451, 62787) | 619.98 (586.90, 653.57) | 56097 (52673, 59649) | 421.24 (394.81, 446.70) | -32.06% (-33.99, -30.02) | 7873 (5512, 10578) | 82.39 (57.63, 110.66) | 7372 (5111, 9886) | 56.00 (38.88, 75.56) | -32.03% (-35.50, -28.57) |
| Republic of Azerbaijan | 23891 (21093, 27032) | 311.64 (276.29, 351.71) | 23405 (20247, 26797) | 223.64 (193.64, 256.71) | -28.24% (-31.49, -25.18) | 33242 (31586, 34791) | 521.11 (495.77, 544.38) | 43590 (41426, 45875) | 372.30 (354.38, 391.89) | -28.56% (-31.21, -25.54) | 4984 (3399, 6845) | 77.67 (53.07, 106.35) | 6459 (4527, 8756) | 55.01 (38.36, 74.27) | -29.17% (-33.18, -24.40) |
| Republic of Belarus | 59011 (51343, 66952) | 563.14 (489.74, 640.98) | 48413 (41308, 55807) | 494.12 (422.06, 572.62) | -12.26% (-16.17, -8.46) | 109946 (105361, 114706) | 930.66 (892.02, 971.29) | 101860 (96489, 106880) | 790.04 (749.68, 829.31) | -15.11% (-17.02, -13.15) | 16167 (11301, 22170) | 137.35 (95.99, 188.69) | 14811 (10406, 20052) | 115.92 (81.10, 157.52) | -15.60% (-19.17, -12.33) |
| Republic of Benin | 7428 (6552, 8378) | 165.14 (147.71, 183.42) | 19127 (17101, 21426) | 163.50 (146.04, 181.73) | -0.99% (-3.54, 1.57) | 7447 (7157, 7721) | 249.89 (240.49, 259.06) | 20957 (20135, 21874) | 254.14 (243.99, 265.11) | 1.70% (0.33, 3.28) | 1102 (773, 1485) | 36.48 (25.95, 49.49) | 3136 (2149, 4297) | 37.41 (26.04, 51.06) | 2.56% (-3.35, 8.57) |
| Republic of Botswana | 2135 (1892, 2410) | 176.93 (156.63, 198.66) | 5164 (4617, 5722) | 215.08 (192.91, 238.53) | 21.56% (16.64, 27.06) | 2379 (2272, 2502) | 277.11 (264.92, 292.59) | 7590 (7249, 7969) | 346.43 (331.04, 363.38) | 25.02% (22.05, 28.32) | 356 (244, 489) | 40.90 (28.02, 55.97) | 1127 (773, 1540) | 50.86 (34.96, 69.16) | 24.35% (16.08, 32.73) |
| Republic of Bulgaria | 52397 (45134, 60323) | 622.96 (535.99, 723.06) | 31817 (27143, 36869) | 495.44 (418.86, 580.68) | -20.47% (-23.51, -17.49) | 104146 (99954, 108891) | 989.48 (949.63, 1036.63) | 74974 (71621, 78753) | 789.42 (751.33, 833.84) | -20.22% (-21.78, -18.68) | 15202 (10631, 20736) | 145.34 (101.40, 198.94) | 10838 (7626, 14605) | 115.85 (80.67, 157.52) | -20.29% (-23.45, -17.37) |
| Republic of Burundi | 8170 (7198, 9253) | 163.22 (145.26, 182.81) | 16383 (14465, 18509) | 143.46 (126.83, 160.91) | -12.11% (-14.62, -9.07) | 7819 (7523, 8153) | 218.75 (210.44, 227.65) | 41942 (29268, 60431) | 472.14 (339.83, 664.20) | 115.84% (54.72, 202.40) | 1160 (814, 1560) | 32.05 (22.51, 42.83) | 6100 (4524, 8286) | 68.16 (50.57, 92.11) | 112.68% (54.38, 204.00) |
| Republic of Cabo Verde | 590 (522, 667) | 174.40 (155.89, 195.94) | 1003 (896, 1122) | 182.14 (162.83, 204.33) | 4.44% (0.74, 8.26) | 730 (697, 763) | 295.30 (280.98, 308.69) | 1575 (1496, 1649) | 296.34 (281.44, 310.28) | 0.35% (-1.77, 2.47) | 109 (76, 152) | 43.81 (30.85, 60.64) | 235 (163, 324) | 43.90 (30.47, 60.35) | 0.21% (-5.54, 6.56) |
| Republic of Cameroon | 14497 (12900, 16229) | 159.42 (142.20, 177.34) | 47253 (42014, 53419) | 172.38 (153.46, 194.25) | 8.13% (3.79, 12.92) | 16353 (15694, 17132) | 249.20 (239.54, 260.54) | 52774 (50476, 55592) | 259.29 (249.16, 271.17) | 4.05% (2.22, 6.15) | 2430 (1719, 3275) | 36.54 (25.97, 48.93) | 7901 (5554, 10635) | 38.21 (26.98, 51.05) | 4.57% (-1.20, 11.07) |
| Republic of Chad | 11032 (9425, 13473) | 186.08 (160.27, 221.33) | 29005 (25549, 33450) | 182.11 (162.24, 204.40) | -2.13% (-9.04, 3.35) | 11336 (9912, 13242) | 271.52 (244.78, 308.09) | 28647 (26641, 31321) | 291.02 (269.75, 319.31) | 7.18% (2.51, 11.19) | 1675 (1231, 2141) | 39.77 (29.25, 51.27) | 4266 (3089, 5666) | 42.60 (30.77, 56.06) | 7.11% (-0.19, 15.12) |
| Republic of Chile | 51327 (40522, 67965) | 375.04 (296.96, 493.73) | 70812 (54480, 94156) | 394.75 (299.27, 532.24) | 5.25% (-1.28, 11.25) | 51774 (49047, 54685) | 436.85 (415.37, 459.78) | 98535 (93288, 104610) | 438.37 (413.91, 469.25) | 0.35% (-1.94, 2.65) | 6858 (4802, 9248) | 57.60 (40.35, 77.17) | 12896 (9142, 17326) | 57.50 (40.64, 77.47) | -0.16% (-4.95, 5.45) |
| Republic of Colombia | 153993 (136672, 172717) | 464.60 (414.02, 517.41) | 144129 (127956, 160799) | 289.90 (256.50, 325.87) | -37.60% (-40.38, -34.79) | 205747 (197034, 215303) | 805.47 (776.21, 837.98) | 293306 (278855, 309340) | 544.84 (517.54, 574.64) | -32.36% (-34.43, -29.95) | 30784 (21752, 41475) | 119.15 (84.33, 161.28) | 43367 (30620, 58257) | 80.61 (56.91, 108.16) | -32.35% (-35.57, -28.62) |
| Republic of Costa Rica | 8966 (7660, 10372) | 309.59 (265.60, 355.47) | 13873 (11972, 15815) | 287.58 (247.09, 330.69) | -7.11% (-9.14, -5.00) | 11444 (10930, 11966) | 499.41 (477.98, 521.78) | 25633 (24638, 26803) | 480.70 (461.54, 502.76) | -3.75% (-5.45, -2.17) | 1699 (1154, 2305) | 73.52 (50.38, 99.26) | 3757 (2618, 5109) | 70.54 (49.13, 95.91) | -4.05% (-7.79, 0.48) |
| Republic of Croatia | 32214 (27542, 37132) | 659.32 (562.78, 762.43) | 30211 (24212, 36931) | 531.82 (439.96, 628.90) | -3.80% (-6.06, -1.36) | 58211 (55806, 60687) | 1022.53 (978.10, 1068.18) | 55305 (52000, 59434) | 825.33 (778.72, 880.13) | 1.35% (-0.19, 3.18) | 8547 (5995, 11540) | 150.43 (105.46, 202.22) | 7968 (5638, 10654) | 120.69 (84.86, 161.35) | 2.69% (-3.09, 8.87) |
| Republic of Cuba | 33132 (28784, 37554) | 300.02 (259.07, 341.95) | 43339 (34894, 52607) | 306.79 (253.27, 364.07) | -19.34% (-24.46, -13.89) | 52923 (50650, 55452) | 491.69 (470.95, 514.20) | 75463 (71471, 79845) | 454.08 (431.21, 477.98) | -19.29% (-21.95, -16.61) | 7863 (5478, 10647) | 72.87 (50.83, 98.65) | 10935 (7680, 14903) | 66.40 (46.50, 91.03) | -19.77% (-23.70, -15.99) |
| Republic of Cyprus | 3379 (2778, 4125) | 436.81 (356.57, 533.39) | 4492 (3597, 5534) | 342.92 (272.82, 429.65) | 2.26% (-4.32, 8.51) | 4488 (4281, 4732) | 556.31 (530.37, 586.37) | 7500 (7139, 7939) | 427.27 (404.90, 453.89) | -7.65% (-10.19, -5.31) | 598 (414, 804) | 73.94 (51.22, 99.64) | 997 (696, 1350) | 56.90 (39.78, 77.27) | -8.87% (-13.70, -4.59) |
| Republic of C么te d'Ivoire | 18693 (16601, 21042) | 182.00 (164.04, 201.74) | 40821 (36397, 45714) | 175.08 (156.58, 194.15) | -21.49% (-26.50, -15.93) | 19400 (18588, 20242) | 272.36 (261.30, 283.39) | 49805 (47760, 51979) | 276.02 (265.12, 288.25) | -23.20% (-25.13, -20.70) | 2880 (1990, 3901) | 39.58 (27.49, 53.91) | 7467 (5163, 10175) | 40.65 (28.34, 55.46) | -23.05% (-26.90, -19.16) |
| Republic of Djibouti | 814 (691, 999) | 202.69 (176.04, 241.04) | 1839 (1647, 2052) | 164.67 (147.78, 183.64) | -18.76% (-28.41, -10.07) | 691 (659, 728) | 258.22 (247.60, 270.35) | 2635 (2509, 2785) | 266.50 (254.14, 280.84) | 3.21% (0.88, 6.08) | 104 (72, 142) | 38.32 (26.56, 52.46) | 394 (279, 529) | 39.28 (27.81, 52.80) | 2.49% (-3.68, 9.08) |
| Republic of Ecuador | 27093 (23857, 30586) | 281.97 (249.95, 316.67) | 52176 (45682, 59146) | 286.45 (250.77, 323.75) | 1.59% (-1.53, 4.77) | 37203 (35557, 38921) | 511.10 (491.04, 531.88) | 90243 (86552, 94278) | 515.59 (494.40, 538.44) | 0.88% (-1.18, 3.12) | 5558 (3920, 7488) | 75.61 (53.35, 102.16) | 13323 (9381, 18021) | 75.92 (53.51, 102.68) | 0.42% (-4.31, 4.84) |
| Republic of El Salvador | 27110 (23135, 33288) | 494.34 (428.48, 589.29) | 24689 (22011, 27509) | 376.11 (335.97, 419.34) | -23.92% (-33.95, -15.20) | 41301 (33532, 53563) | 966.52 (819.93, 1195.27) | 47093 (43379, 52776) | 756.32 (695.66, 851.33) | -21.75% (-29.31, -14.04) | 6092 (4606, 7952) | 141.78 (106.53, 181.57) | 6885 (5032, 9015) | 110.45 (81.05, 144.43) | -22.10% (-30.82, -13.67) |
| Republic of Equatorial Guinea | 730 (647, 818) | 185.37 (164.82, 206.16) | 1984 (1759, 2239) | 153.04 (135.43, 172.09) | -17.44% (-21.26, -13.69) | 798 (767, 842) | 278.42 (267.05, 293.90) | 2293 (2193, 2424) | 239.95 (229.46, 252.45) | -13.82% (-15.60, -12.18) | 118 (82, 161) | 40.64 (28.30, 55.34) | 344 (235, 467) | 35.15 (24.40, 47.79) | -13.50% (-18.80, -8.08) |
| Republic of Estonia | 11404 (9939, 12922) | 718.96 (627.18, 816.51) | 5313 (4536, 6127) | 388.73 (329.46, 451.91) | -45.93% (-48.40, -43.38) | 21089 (20279, 21988) | 1158.76 (1113.90, 1208.06) | 11969 (11393, 12590) | 643.66 (612.69, 675.97) | -44.45% (-45.87, -42.94) | 3093 (2167, 4209) | 170.75 (119.62, 232.13) | 1732 (1218, 2345) | 94.41 (65.79, 128.33) | -44.71% (-47.12, -42.28) |
| Republic of Fiji | 1047 (926, 1181) | 142.68 (126.57, 160.21) | 1185 (1053, 1332) | 132.67 (117.45, 148.59) | -7.02% (-9.90, -3.90) | 1430 (1364, 1507) | 244.25 (233.90, 256.12) | 2015 (1923, 2126) | 224.89 (214.71, 236.79) | -7.93% (-10.02, -5.97) | 215 (148, 292) | 36.15 (25.12, 49.39) | 298 (209, 400) | 33.09 (23.20, 44.38) | -8.46% (-14.36, -2.67) |
| Republic of Finland | 24444 (19459, 30399) | 494.20 (391.97, 616.36) | 22715 (17653, 29021) | 397.45 (304.83, 509.37) | -19.58% (-23.54, -15.85) | 37519 (35511, 39593) | 611.44 (577.06, 645.81) | 43034 (40244, 45964) | 492.29 (461.79, 526.45) | -19.49% (-21.65, -17.30) | 4946 (3507, 6598) | 80.95 (57.24, 108.20) | 5615 (3975, 7480) | 65.21 (45.73, 87.10) | -19.45% (-23.13, -15.42) |
| Republic of Ghana | 19066 (16988, 21403) | 143.75 (127.74, 160.63) | 44283 (39289, 49966) | 148.17 (130.58, 166.34) | 3.08% (-0.10, 6.20) | 21138 (20274, 22074) | 220.86 (211.94, 230.49) | 55386 (53091, 57889) | 227.28 (217.38, 237.18) | 2.91% (1.46, 4.30) | 3153 (2199, 4313) | 32.50 (22.78, 44.10) | 8264 (5802, 11174) | 33.48 (23.59, 45.37) | 3.00% (-2.86, 9.05) |
| Republic of Guatemala | 34085 (28933, 40431) | 416.79 (360.64, 484.37) | 56674 (49599, 64498) | 361.01 (315.85, 409.13) | -13.38% (-24.24, -5.66) | 39635 (34495, 48399) | 696.71 (623.19, 819.73) | 85670 (81006, 92082) | 645.56 (610.72, 698.20) | -7.34% (-15.34, -0.95) | 5840 (4220, 7733) | 101.76 (73.36, 133.60) | 12517 (8953, 16647) | 93.64 (67.12, 124.46) | -7.99% (-17.13, -0.25) |
| Republic of Guinea | 9322 (8204, 10449) | 157.81 (140.83, 175.73) | 19757 (17639, 22109) | 166.36 (149.87, 184.80) | 5.42% (2.71, 8.29) | 10278 (9885, 10706) | 239.79 (231.16, 249.76) | 22626 (21776, 23579) | 265.26 (255.46, 276.42) | 10.62% (8.72, 12.77) | 1520 (1058, 2059) | 35.18 (24.42, 48.03) | 3376 (2394, 4615) | 39.05 (27.80, 53.32) | 11.00% (4.87, 18.46) |
| Republic of Guinea-Bissau | 1962 (1742, 2200) | 214.10 (192.23, 237.21) | 3359 (3001, 3733) | 190.74 (171.01, 212.20) | -10.91% (-13.24, -8.54) | 2031 (1951, 2121) | 318.02 (306.06, 332.39) | 3893 (3735, 4088) | 300.70 (288.71, 314.57) | -5.45% (-7.40, -3.25) | 303 (208, 426) | 46.75 (32.35, 64.77) | 584 (411, 787) | 44.31 (31.45, 59.82) | -5.23% (-10.59, 0.43) |
| Republic of Guyana | 1863 (1660, 2091) | 250.65 (223.01, 281.16) | 2211 (1970, 2465) | 290.07 (258.82, 324.22) | 15.73% (12.40, 18.95) | 2199 (2112, 2292) | 383.51 (368.28, 399.72) | 3363 (3242, 3505) | 457.61 (441.54, 476.89) | 19.32% (17.36, 21.30) | 327 (227, 447) | 56.20 (39.60, 76.30) | 492 (346, 666) | 66.64 (46.87, 90.28) | 18.57% (12.77, 24.80) |
| Republic of Haiti | 16655 (14655, 19141) | 261.41 (231.45, 297.44) | 41005 (34972, 48510) | 311.39 (268.20, 364.81) | 19.12% (7.87, 36.23) | 19863 (19141, 20822) | 414.86 (400.38, 434.27) | 74280 (59645, 100195) | 644.02 (524.69, 849.35) | 55.24% (27.39, 103.32) | 2946 (2052, 4004) | 60.88 (42.31, 82.27) | 10974 (8056, 14417) | 94.12 (70.16, 122.82) | 54.60% (26.44, 105.52) |
| Republic of Honduras | 15693 (13681, 18073) | 328.82 (291.00, 371.29) | 31214 (27636, 35346) | 312.96 (278.77, 352.53) | -4.82% (-8.55, -1.07) | 17610 (16976, 18416) | 567.84 (548.97, 592.86) | 47203 (44970, 49698) | 561.55 (538.04, 588.17) | -1.11% (-4.13, 2.21) | 2633 (1826, 3573) | 84.00 (58.57, 113.95) | 7011 (4971, 9501) | 82.60 (58.41, 112.17) | -1.66% (-6.65, 3.96) |
| Republic of Iceland | 970 (779, 1208) | 378.85 (303.78, 471.38) | 992 (778, 1259) | 293.00 (223.53, 375.31) | -22.66% (-27.67, -17.53) | 1276 (1210, 1350) | 476.83 (451.86, 504.81) | 1638 (1541, 1746) | 361.39 (338.79, 386.46) | -24.21% (-26.41, -21.39) | 170 (118, 232) | 63.53 (44.27, 87.10) | 217 (151, 292) | 48.18 (33.35, 65.27) | -24.16% (-28.17, -19.69) |
| Republic of India | 2181010 (1890440, 2508152) | 300.53 (253.59, 348.74) | 3557175 (2997681, 4164652) | 268.45 (222.87, 314.93) | -10.67% (-14.31, -7.21) | 2529463 (2403563, 2660978) | 413.57 (392.84, 435.91) | 5359211 (5077920, 5661666) | 413.16 (389.72, 437.92) | -0.10% (-1.49, 1.27) | 370692 (261668, 509817) | 59.69 (42.40, 81.52) | 780244 (552571, 1057632) | 59.66 (42.35, 80.59) | -0.05% (-1.95, 1.76) |
| Republic of Indonesia | 393083 (340476, 454544) | 223.13 (193.88, 256.69) | 432654 (378797, 493650) | 164.77 (143.17, 188.97) | -26.15% (-29.06, -23.01) | 554715 (526463, 597270) | 384.37 (365.76, 408.81) | 781431 (741944, 829307) | 281.88 (267.85, 298.31) | -26.67% (-28.77, -24.05) | 83225 (58368, 111319) | 56.89 (40.07, 75.99) | 116291 (82912, 155561) | 41.60 (29.62, 55.76) | -26.87% (-29.25, -24.01) |
| Republic of Iraq | 75476 (65547, 87920) | 413.53 (361.32, 476.05) | 129119 (113593, 145608) | 313.23 (276.57, 351.25) | -24.26% (-28.09, -20.02) | 142543 (109620, 186292) | 1028.85 (828.05, 1299.21) | 291320 (237204, 370033) | 839.00 (684.53, 1054.35) | -18.45% (-20.99, -15.67) | 20947 (15575, 27427) | 150.10 (112.99, 191.98) | 42290 (32048, 53795) | 120.42 (91.52, 152.73) | -19.78% (-23.45, -15.82) |
| Republic of Italy | 273755 (218734, 344776) | 478.19 (384.01, 605.57) | 191226 (148198, 241797) | 330.97 (256.41, 421.69) | -30.79% (-36.09, -25.17) | 448390 (425295, 473287) | 616.92 (584.68, 653.05) | 399763 (376198, 425237) | 416.73 (390.50, 444.62) | -32.45% (-34.06, -30.53) | 59026 (41353, 78990) | 81.68 (57.14, 109.85) | 52308 (36438, 70302) | 55.43 (38.46, 75.32) | -32.14% (-34.05, -30.05) |
| Republic of Kazakhstan | 76462 (67417, 86237) | 455.49 (402.70, 512.74) | 70543 (62040, 79676) | 370.70 (324.23, 419.20) | -18.62% (-21.12, -16.13) | 111503 (106906, 116257) | 736.71 (706.92, 767.87) | 120317 (115113, 126007) | 610.40 (583.66, 639.25) | -17.15% (-18.78, -15.59) | 16599 (11619, 22773) | 109.13 (76.56, 149.62) | 17750 (12373, 24096) | 89.84 (62.66, 121.68) | -17.67% (-20.68, -14.20) |
| Republic of Kenya | 32434 (28291, 36807) | 171.16 (151.53, 193.27) | 70607 (62406, 79757) | 172.59 (153.75, 193.45) | 0.83% (-1.78, 3.18) | 34066 (32380, 35665) | 265.71 (252.63, 277.86) | 90586 (85702, 95129) | 270.35 (256.58, 282.87) | 1.74% (0.59, 2.96) | 5069 (3535, 6977) | 38.85 (27.18, 53.38) | 13500 (9526, 18530) | 39.61 (27.93, 54.12) | 1.96% (0.44, 3.70) |
| Republic of Kiribati | 121 (107, 135) | 165.92 (147.85, 185.54) | 150 (134, 168) | 126.63 (113.67, 142.03) | -23.68% (-27.36, -19.67) | 161 (153, 171) | 284.19 (269.84, 299.67) | 224 (214, 236) | 216.22 (207.22, 227.15) | -23.92% (-26.74, -21.29) | 24 (17, 33) | 42.15 (29.30, 57.36) | 34 (23, 45) | 32.06 (22.28, 43.07) | -23.95% (-29.51, -18.64) |
| Republic of Korea | 251612 (207886, 310297) | 545.80 (453.21, 667.11) | 167704 (135596, 206007) | 309.36 (247.82, 387.65) | -43.32% (-47.47, -38.21) | 276718 (263621, 290659) | 696.24 (664.25, 727.82) | 311099 (294712, 327983) | 397.94 (377.04, 420.40) | -42.84% (-44.53, -41.06) | 37046 (25906, 50452) | 92.15 (64.47, 124.98) | 41127 (28640, 55211) | 52.82 (36.70, 71.32) | -42.68% (-45.38, -39.59) |
| Republic of Latvia | 21778 (18941, 24737) | 799.49 (694.33, 907.57) | 8753 (7529, 10025) | 439.40 (379.81, 506.04) | -45.04% (-46.91, -42.85) | 40522 (38719, 42445) | 1287.05 (1228.98, 1347.19) | 19310 (18408, 20224) | 710.90 (679.79, 743.52) | -44.76% (-46.14, -43.43) | 5925 (4157, 8103) | 189.27 (132.76, 259.23) | 2789 (1979, 3777) | 104.24 (73.60, 141.15) | -44.92% (-47.17, -42.72) |
| Republic of Liberia | 15236 (10201, 22864) | 574.41 (392.11, 847.42) | 5988 (5302, 6796) | 129.50 (114.52, 145.22) | -77.46% (-84.85, -66.05) | 5034 (4348, 5974) | 265.71 (239.74, 300.53) | 10606 (8690, 13253) | 283.72 (238.82, 347.47) | 6.78% (-5.32, 22.32) | 762 (521, 1051) | 39.52 (27.17, 53.42) | 1540 (1144, 1964) | 40.72 (30.45, 51.26) | 3.04% (-7.92, 19.03) |
| Republic of Lithuania | 26551 (23151, 30022) | 703.17 (612.21, 795.05) | 13836 (11671, 15940) | 450.27 (385.54, 516.95) | -35.97% (-38.92, -33.43) | 48099 (45904, 50242) | 1156.04 (1103.71, 1206.56) | 30241 (28746, 31836) | 734.83 (700.37, 771.38) | -36.44% (-37.79, -34.95) | 7043 (4948, 9633) | 169.85 (119.41, 232.28) | 4363 (3059, 5918) | 107.58 (75.22, 147.07) | -36.66% (-39.05, -34.04) |
| Republic of Madagascar | 16158 (14140, 18350) | 140.85 (124.60, 158.29) | 30196 (26695, 34181) | 118.75 (105.72, 132.76) | -15.69% (-17.85, -13.24) | 16354 (15708, 17028) | 206.88 (199.14, 215.10) | 33054 (31640, 34604) | 175.82 (168.85, 183.62) | -15.01% (-16.57, -13.51) | 2426 (1681, 3325) | 30.30 (20.92, 41.58) | 4931 (3455, 6686) | 25.84 (18.19, 34.63) | -14.72% (-20.05, -8.70) |
| Republic of Malawi | 12580 (10994, 14335) | 135.36 (120.04, 152.30) | 21269 (18837, 24089) | 127.89 (113.27, 144.10) | -5.52% (-8.65, -2.60) | 11786 (11302, 12314) | 185.38 (178.07, 193.34) | 22395 (21444, 23354) | 183.63 (176.07, 191.86) | -0.94% (-2.50, 0.89) | 1742 (1227, 2385) | 27.02 (19.23, 37.07) | 3331 (2308, 4634) | 26.88 (18.83, 36.54) | -0.50% (-7.44, 6.63) |
| Republic of Maldives | 364 (316, 420) | 182.06 (157.86, 207.41) | 848 (721, 988) | 167.47 (141.84, 194.85) | -8.01% (-14.01, -2.12) | 409 (391, 426) | 297.98 (286.35, 309.96) | 1467 (1389, 1551) | 287.07 (270.91, 303.10) | -3.66% (-7.02, -0.04) | 61 (43, 83) | 43.97 (31.20, 59.63) | 220 (157, 298) | 42.46 (30.24, 56.96) | -3.43% (-9.44, 3.07) |
| Republic of Mali | 16624 (14688, 19119) | 202.22 (179.53, 228.00) | 39307 (34839, 44584) | 183.37 (162.64, 204.70) | -9.32% (-13.46, -5.87) | 15938 (15273, 16658) | 278.13 (265.57, 289.89) | 44005 (40848, 48314) | 308.39 (287.00, 336.74) | 10.88% (4.99, 19.05) | 2370 (1649, 3243) | 40.84 (28.58, 55.64) | 6561 (4732, 8741) | 45.28 (32.69, 60.07) | 10.89% (3.18, 20.92) |
| Republic of Malta | 1237 (979, 1562) | 346.90 (271.08, 438.56) | 1251 (977, 1593) | 311.13 (236.70, 399.98) | -10.31% (-14.05, -6.58) | 1705 (1621, 1805) | 420.58 (399.54, 444.49) | 2450 (2311, 2602) | 378.29 (354.77, 403.56) | -10.06% (-12.33, -7.73) | 227 (159, 304) | 55.91 (39.24, 75.37) | 323 (228, 437) | 50.34 (35.26, 68.76) | -9.96% (-14.59, -5.25) |
| Republic of Mauritius | 1703 (1507, 1921) | 154.02 (136.13, 173.05) | 2000 (1736, 2256) | 147.47 (128.30, 167.68) | -4.25% (-7.95, -0.39) | 2459 (2357, 2564) | 255.76 (245.29, 266.08) | 4266 (4089, 4461) | 258.35 (247.70, 269.91) | 1.01% (-1.40, 3.73) | 366 (254, 495) | 37.79 (26.18, 50.95) | 625 (432, 847) | 38.02 (26.17, 51.68) | 0.63% (-5.27, 7.43) |
| Republic of Moldova | 26209 (23047, 29651) | 586.46 (516.18, 663.12) | 12876 (11221, 14579) | 350.82 (305.94, 399.00) | -40.18% (-42.26, -38.25) | 43851 (42216, 45654) | 958.01 (922.86, 997.34) | 28148 (26963, 29408) | 577.84 (553.69, 602.78) | -39.68% (-40.98, -38.44) | 6471 (4544, 8790) | 141.23 (99.16, 192.01) | 4112 (2882, 5573) | 85.09 (59.40, 115.86) | -39.75% (-42.46, -37.22) |
| Republic of Mozambique | 26131 (21461, 34343) | 200.49 (168.53, 253.01) | 46804 (41698, 52877) | 176.89 (158.00, 198.14) | -11.77% (-25.59, -0.51) | 37271 (27748, 55612) | 367.20 (287.89, 526.67) | 52668 (47756, 60303) | 287.64 (258.64, 337.55) | -21.67% (-35.46, -9.44) | 5450 (3909, 8059) | 53.20 (38.97, 74.89) | 7738 (5561, 10063) | 41.51 (30.20, 53.25) | -21.98% (-36.75, -8.40) |
| Republic of Namibia | 2737 (2441, 3063) | 215.65 (193.62, 237.92) | 4983 (4476, 5519) | 211.62 (189.96, 234.34) | -1.87% (-4.53, 0.92) | 4247 (3843, 4840) | 427.10 (394.67, 472.37) | 7417 (7060, 7819) | 376.39 (358.51, 397.53) | -11.87% (-17.47, -7.71) | 634 (462, 815) | 63.22 (45.50, 82.61) | 1105 (771, 1496) | 55.41 (39.09, 74.93) | -12.36% (-19.43, -6.60) |
| Republic of Nauru | 23 (20, 25) | 245.23 (219.31, 274.17) | 26 (24, 30) | 256.78 (227.47, 287.69) | 4.71% (1.26, 8.10) | 31 (29, 33) | 420.06 (400.13, 443.96) | 37 (35, 38) | 422.97 (403.88, 444.30) | 0.69% (-1.25, 2.61) | 5 (3, 6) | 62.70 (43.49, 84.78) | 5 (4, 8) | 62.71 (44.02, 85.88) | 0.02% (-5.09, 5.55) |
| Republic of Nicaragua | 10657 (9173, 12458) | 268.80 (234.65, 306.49) | 14735 (12717, 17049) | 232.71 (200.45, 266.96) | -13.42% (-16.81, -9.86) | 21072 (15045, 31029) | 707.16 (545.85, 969.90) | 28429 (24175, 34973) | 489.56 (414.87, 608.85) | -30.77% (-37.68, -23.57) | 3095 (2167, 4439) | 103.17 (74.70, 142.22) | 4120 (3073, 5273) | 70.48 (52.61, 90.54) | -31.68% (-39.90, -23.70) |
| Republic of Niue | 4 (4, 5) | 192.68 (172.34, 215.06) | 3 (3, 3) | 175.42 (154.30, 197.19) | -8.96% (-12.61, -5.31) | 7 (7, 7) | 333.45 (317.75, 349.97) | 6 (6, 6) | 306.54 (293.12, 320.99) | -8.07% (-10.67, -5.66) | 1 (1, 1) | 49.68 (34.16, 67.95) | 1 (1, 1) | 45.15 (31.70, 61.25) | -9.12% (-14.24, -3.40) |
| Republic of Palau | 44 (38, 49) | 298.20 (261.45, 335.45) | 57 (49, 64) | 309.63 (266.66, 353.25) | 3.83% (0.39, 7.74) | 62 (59, 65) | 480.17 (458.68, 504.45) | 115 (110, 121) | 502.24 (479.50, 526.42) | 4.60% (2.44, 6.71) | 9 (6, 13) | 71.26 (49.89, 97.11) | 17 (12, 23) | 73.77 (50.86, 100.94) | 3.52% (-1.42, 9.09) |
| Republic of Panama | 7172 (6236, 8144) | 299.21 (261.88, 337.30) | 10670 (9313, 12123) | 247.94 (216.03, 281.86) | -17.14% (-19.37, -14.61) | 10036 (9479, 10969) | 520.05 (495.53, 558.24) | 19222 (18420, 20287) | 437.47 (419.13, 461.46) | -15.88% (-18.19, -14.03) | 1495 (1046, 2004) | 76.89 (54.00, 102.93) | 2828 (1990, 3838) | 64.38 (45.27, 87.36) | -16.26% (-20.51, -12.49) |
| Republic of Paraguay | 10699 (9047, 12639) | 266.57 (228.16, 311.65) | 20641 (17911, 23475) | 283.93 (246.17, 322.22) | 6.52% (1.71, 11.33) | 13550 (12819, 14370) | 457.62 (435.15, 483.19) | 33719 (32083, 35456) | 501.28 (477.62, 526.46) | 9.54% (6.37, 13.04) | 2006 (1369, 2728) | 67.24 (46.06, 91.14) | 4975 (3525, 6721) | 73.58 (52.10, 99.03) | 9.42% (3.71, 15.62) |
| Republic of Peru | 61905 (52806, 74218) | 276.48 (239.69, 324.73) | 75262 (65520, 86024) | 205.16 (178.89, 234.43) | -25.79% (-35.80, -16.87) | 67576 (62877, 74588) | 402.46 (380.79, 431.62) | 134277 (127903, 141657) | 373.34 (355.67, 393.19) | -7.24% (-10.19, -4.51) | 10088 (7310, 13334) | 59.56 (43.02, 78.95) | 19823 (14143, 26670) | 54.98 (39.30, 73.90) | -7.68% (-13.10, -2.17) |
| Republic of Poland | 244316 (209059, 283787) | 632.49 (540.70, 733.18) | 198090 (166446, 231182) | 466.00 (393.24, 545.22) | -26.32% (-29.05, -23.74) | 421793 (401118, 444253) | 1012.35 (964.23, 1065.59) | 423535 (399970, 447475) | 766.24 (726.67, 806.99) | -24.31% (-25.71, -22.86) | 61528 (43172, 83644) | 147.93 (103.75, 201.78) | 61188 (43139, 83359) | 111.95 (78.58, 153.59) | -24.32% (-25.92, -22.77) |
| Republic of Rwanda | 22375 (17883, 29974) | 311.48 (255.51, 403.58) | 16910 (15063, 18906) | 146.02 (130.18, 163.05) | -53.12% (-63.28, -42.52) | 14218 (13488, 15180) | 296.29 (284.34, 309.99) | 40061 (30594, 55299) | 421.98 (323.62, 579.89) | 42.42% (11.12, 90.97) | 2137 (1471, 2896) | 43.86 (30.63, 59.27) | 5847 (4352, 7651) | 60.95 (45.31, 79.49) | 38.96% (8.85, 90.17) |
| Republic of San Marino | 77 (63, 96) | 337.21 (270.13, 426.35) | 92 (74, 113) | 302.73 (235.36, 390.76) | -10.23% (-14.03, -6.31) | 122 (117, 129) | 423.72 (404.04, 448.04) | 188 (179, 198) | 373.76 (351.94, 398.53) | -11.79% (-14.01, -9.63) | 16 (11, 22) | 56.47 (39.37, 76.04) | 25 (17, 33) | 49.60 (34.40, 67.49) | -12.17% (-16.79, -6.91) |
| Republic of Senegal | 9877 (8711, 11241) | 138.67 (123.00, 155.67) | 18053 (15912, 20563) | 132.17 (115.42, 149.92) | -4.69% (-8.17, -1.08) | 9335 (8930, 9740) | 194.36 (185.78, 203.21) | 21268 (20380, 22282) | 199.56 (190.78, 209.33) | 2.67% (0.70, 4.64) | 1379 (948, 1862) | 28.40 (19.85, 38.20) | 3141 (2212, 4295) | 29.15 (20.64, 39.47) | 2.63% (-3.93, 10.04) |
| Republic of Serbia | 48008 (41450, 55473) | 508.29 (438.92, 587.87) | 38222 (32210, 44356) | 410.69 (346.26, 480.37) | -19.20% (-22.36, -15.73) | 90426 (86760, 94400) | 830.08 (795.60, 866.92) | 81584 (77735, 85936) | 668.93 (636.22, 707.08) | -19.41% (-21.81, -16.69) | 13334 (9288, 18306) | 122.57 (85.32, 167.89) | 11822 (8366, 15965) | 97.98 (69.28, 131.95) | -20.06% (-23.77, -15.98) |
| Republic of Seychelles | 141 (125, 158) | 197.23 (174.44, 220.41) | 190 (168, 212) | 174.05 (153.43, 194.85) | -11.75% (-15.02, -8.60) | 207 (199, 216) | 336.11 (322.58, 350.38) | 373 (358, 389) | 305.81 (294.05, 318.64) | -9.01% (-10.51, -7.48) | 31 (21, 43) | 50.10 (34.78, 69.04) | 55 (38, 74) | 45.23 (31.49, 61.02) | -9.73% (-14.98, -4.45) |
| Republic of Sierra Leone | 6146 (5418, 6905) | 146.44 (130.14, 163.61) | 10839 (9621, 12226) | 137.43 (122.03, 154.13) | -6.15% (-8.94, -3.66) | 6285 (6060, 6534) | 212.16 (204.67, 220.45) | 15880 (14059, 18294) | 264.27 (235.71, 303.23) | 24.56% (11.73, 42.39) | 930 (647, 1268) | 31.01 (21.77, 42.26) | 2341 (1743, 3003) | 38.54 (28.81, 49.12) | 24.26% (10.11, 44.24) |
| Republic of Singapore | 10249 (8249, 12890) | 316.24 (255.70, 397.04) | 12288 (9574, 15862) | 245.92 (189.83, 323.99) | -22.24% (-27.11, -17.63) | 11439 (10827, 12151) | 382.79 (363.55, 404.07) | 22430 (21233, 23847) | 297.40 (280.78, 316.89) | -22.31% (-24.41, -20.16) | 1540 (1073, 2091) | 51.07 (35.86, 69.06) | 2998 (2103, 4052) | 39.74 (27.75, 53.42) | -22.17% (-26.58, -17.34) |
| Republic of Slovenia | 17880 (14983, 20817) | 868.47 (732.18, 1014.04) | 16391 (13158, 19778) | 621.57 (512.13, 736.04) | -28.43% (-31.60, -25.08) | 30870 (29532, 32428) | 1347.16 (1291.13, 1414.09) | 32377 (30411, 34433) | 974.61 (924.09, 1031.16) | -27.65% (-29.49, -25.61) | 4502 (3164, 6151) | 197.05 (138.46, 269.28) | 4646 (3262, 6269) | 142.09 (98.72, 193.11) | -27.89% (-30.56, -25.08) |
| Republic of South Africa | 150972 (128623, 175746) | 417.04 (355.13, 483.40) | 160608 (137703, 182854) | 267.71 (232.05, 304.31) | -35.81% (-38.11, -33.06) | 231579 (216290, 249681) | 828.22 (771.82, 891.74) | 269099 (251944, 287535) | 471.61 (442.38, 503.43) | -43.06% (-44.08, -42.12) | 34659 (23920, 47039) | 122.66 (84.99, 166.49) | 39766 (27366, 54107) | 69.25 (47.72, 94.09) | -43.54% (-44.92, -42.21) |
| Republic of South Sudan | 8514 (7489, 9605) | 156.32 (139.10, 173.94) | 16174 (14134, 18997) | 180.65 (159.85, 205.50) | 15.56% (7.71, 26.42) | 10842 (9798, 12300) | 264.77 (245.83, 291.70) | 20152 (17375, 24285) | 306.88 (270.46, 362.30) | 15.91% (8.87, 24.87) | 1594 (1149, 2097) | 38.45 (27.69, 50.76) | 2958 (2179, 3807) | 44.41 (32.98, 57.21) | 15.48% (6.14, 27.21) |
| Republic of Sudan | 75126 (62873, 94022) | 372.88 (317.77, 455.14) | 100421 (88548, 113424) | 244.70 (216.77, 273.24) | -34.38% (-43.32, -26.41) | 70475 (66561, 75139) | 504.75 (480.77, 534.39) | 146281 (136394, 159396) | 473.22 (443.01, 511.89) | -6.25% (-9.23, -2.61) | 10531 (7364, 14235) | 74.54 (52.33, 100.48) | 21770 (15464, 28616) | 69.48 (49.63, 91.66) | -6.79% (-11.91, -1.05) |
| Republic of Suriname | 805 (712, 905) | 206.07 (183.02, 231.04) | 1211 (1070, 1372) | 208.71 (184.36, 236.92) | 1.28% (-1.62, 4.35) | 1224 (1130, 1434) | 373.12 (348.21, 422.78) | 2279 (2165, 2456) | 362.08 (344.39, 388.38) | -2.96% (-9.31, 0.16) | 182 (129, 248) | 55.19 (38.57, 75.87) | 333 (236, 444) | 52.94 (37.62, 70.72) | -4.08% (-11.22, 3.13) |
| Republic of Tajikistan | 17565 (15305, 20010) | 323.45 (283.44, 366.51) | 22330 (19372, 25541) | 217.25 (189.05, 247.36) | -32.83% (-34.95, -30.78) | 20109 (19218, 21074) | 534.06 (510.81, 557.77) | 34311 (31679, 37666) | 399.94 (369.89, 439.30) | -25.11% (-30.35, -18.11) | 3007 (2057, 4087) | 79.29 (54.48, 107.09) | 5094 (3604, 6706) | 58.92 (42.02, 77.35) | -25.69% (-31.67, -17.46) |
| Republic of the Congo | 4074 (3623, 4587) | 191.58 (170.89, 214.55) | 7804 (6948, 8724) | 161.96 (144.62, 180.99) | -15.46% (-17.98, -12.82) | 4594 (4418, 4836) | 288.42 (276.63, 304.45) | 13024 (11703, 14955) | 307.71 (281.00, 347.67) | 6.69% (-2.31, 20.48) | 684 (471, 931) | 42.38 (29.34, 57.21) | 1928 (1400, 2528) | 45.02 (32.73, 59.02) | 6.22% (-3.74, 21.14) |
| Republic of the Gambia | 1130 (981, 1293) | 129.82 (114.14, 146.40) | 2640 (2314, 2989) | 134.53 (116.87, 152.35) | 3.63% (0.30, 7.31) | 1216 (1140, 1308) | 201.58 (191.80, 213.94) | 2907 (2785, 3047) | 198.16 (189.17, 208.31) | -1.70% (-4.53, 0.78) | 181 (130, 240) | 29.58 (21.15, 39.10) | 429 (300, 582) | 28.86 (20.35, 38.82) | -2.43% (-8.91, 4.11) |
| Republic of the Marshall Islands | 82 (72, 91) | 210.42 (188.10, 233.69) | 105 (93, 117) | 194.85 (173.25, 217.90) | -7.40% (-10.33, -4.23) | 97 (92, 103) | 359.41 (341.87, 378.62) | 163 (155, 172) | 331.50 (315.30, 348.14) | -7.77% (-9.56, -6.02) | 15 (10, 20) | 53.53 (36.41, 73.69) | 24 (17, 34) | 48.81 (33.91, 67.00) | -8.83% (-13.61, -3.94) |
| Republic of the Niger | 13601 (12014, 15347) | 182.28 (163.14, 202.95) | 38643 (34095, 44202) | 176.20 (156.14, 197.58) | -3.33% (-6.25, -0.16) | 12823 (12319, 13343) | 268.21 (256.78, 279.91) | 36060 (34437, 37942) | 261.01 (249.50, 273.47) | -2.69% (-4.65, -0.64) | 1914 (1313, 2626) | 39.42 (27.09, 53.97) | 5413 (3800, 7304) | 38.54 (26.91, 52.53) | -2.21% (-7.59, 3.92) |
| Republic of the Philippines | 175512 (153744, 200694) | 285.30 (251.94, 324.13) | 204381 (181798, 230583) | 184.39 (163.84, 207.36) | -35.37% (-38.84, -31.86) | 221631 (206134, 244063) | 487.08 (459.26, 525.64) | 353281 (332399, 379467) | 347.22 (327.38, 373.43) | -28.71% (-30.30, -26.77) | 33277 (23495, 44568) | 72.14 (51.03, 96.87) | 52665 (37544, 69879) | 51.35 (36.64, 68.06) | -28.82% (-30.58, -26.63) |
| Republic of the Union of Myanmar | 100581 (88771, 113781) | 252.78 (223.35, 285.85) | 143726 (121984, 173744) | 257.01 (217.21, 308.52) | 1.67% (-7.69, 13.97) | 132724 (121519, 162609) | 408.22 (380.24, 478.86) | 214974 (193160, 249627) | 394.16 (355.89, 454.08) | -3.44% (-11.42, 6.30) | 19803 (13890, 26596) | 60.30 (42.90, 80.93) | 31769 (23711, 40225) | 57.92 (43.17, 73.28) | -3.96% (-13.52, 7.82) |
| Republic of Trinidad and Tobago | 2702 (2385, 3062) | 225.17 (199.29, 253.72) | 3138 (2801, 3498) | 229.87 (204.42, 258.80) | 2.09% (-1.74, 6.61) | 3662 (3524, 3824) | 354.22 (341.46, 369.44) | 6750 (6447, 7068) | 397.16 (379.14, 416.56) | 12.13% (9.40, 15.59) | 545 (387, 745) | 52.43 (37.19, 71.59) | 987 (695, 1336) | 58.42 (41.07, 79.25) | 11.43% (5.57, 18.56) |
| Republic of Tunisia | 26434 (23226, 30089) | 317.14 (279.39, 358.44) | 31611 (27470, 35741) | 263.72 (229.34, 298.83) | -16.84% (-20.08, -12.94) | 38056 (36498, 39845) | 579.76 (556.50, 607.34) | 64382 (61564, 67486) | 480.83 (460.31, 503.23) | -17.06% (-18.91, -15.24) | 5685 (3987, 7806) | 85.86 (60.32, 117.54) | 9449 (6649, 12818) | 70.57 (49.71, 95.78) | -17.81% (-21.44, -13.55) |
| Republic of Turkey | 136654 (120555, 156074) | 236.21 (209.54, 268.35) | 172085 (146720, 198098) | 204.87 (175.16, 236.29) | -13.27% (-18.52, -7.00) | 186760 (179612, 194546) | 397.00 (381.85, 413.03) | 330300 (315026, 345922) | 355.58 (338.62, 372.73) | -10.43% (-13.20, -7.59) | 27993 (19423, 38267) | 58.99 (41.19, 80.43) | 48459 (33819, 65060) | 52.14 (36.42, 70.25) | -11.61% (-16.49, -6.22) |
| Republic of Uganda | 27178 (23941, 31219) | 168.87 (151.08, 189.52) | 57281 (51054, 64292) | 155.31 (139.07, 171.97) | -8.03% (-14.06, -2.96) | 38706 (29754, 53669) | 316.39 (258.74, 411.97) | 69323 (63779, 77504) | 279.90 (254.57, 317.47) | -11.53% (-23.57, -0.23) | 5667 (4068, 7958) | 45.87 (33.60, 61.14) | 10301 (7311, 13577) | 40.83 (29.51, 53.76) | -11.00% (-25.88, 1.80) |
| Republic of Uzbekistan | 65673 (57519, 75329) | 304.60 (269.40, 347.11) | 88068 (77428, 100052) | 255.79 (224.69, 290.94) | -16.03% (-18.12, -13.94) | 77973 (74897, 81603) | 499.51 (481.00, 521.31) | 136516 (130714, 142510) | 406.24 (389.46, 423.78) | -18.67% (-20.15, -17.35) | 11629 (8109, 15747) | 73.96 (51.67, 100.37) | 20334 (14102, 27906) | 60.17 (41.68, 82.59) | -18.64% (-22.54, -14.93) |
| Republic of Vanuatu | 227 (202, 257) | 167.22 (147.89, 187.45) | 481 (426, 542) | 165.65 (146.38, 185.80) | -0.94% (-3.91, 2.17) | 299 (283, 321) | 293.24 (279.80, 310.99) | 719 (685, 762) | 293.04 (280.69, 309.27) | -0.07% (-1.74, 1.85) | 45 (31, 60) | 43.53 (30.10, 58.45) | 108 (75, 146) | 43.44 (30.25, 58.46) | -0.19% (-5.99, 6.18) |
| Republic of Yemen | 44673 (39025, 51097) | 347.00 (304.36, 391.60) | 169166 (135224, 228186) | 495.42 (406.84, 646.07) | 42.77% (18.14, 82.03) | 53124 (50436, 56166) | 633.49 (602.01, 670.21) | 168083 (150110, 190875) | 672.32 (613.57, 743.53) | 6.13% (-0.76, 14.39) | 7968 (5632, 10679) | 93.67 (65.68, 125.37) | 25159 (18160, 32279) | 99.28 (71.73, 128.10) | 5.98% (-1.67, 16.12) |
| Republic of Zambia | 11739 (10417, 13211) | 162.03 (145.56, 180.64) | 27442 (24551, 30787) | 172.71 (155.04, 192.12) | 6.59% (2.25, 10.78) | 11257 (10799, 11731) | 232.18 (222.86, 242.14) | 31097 (29657, 32551) | 260.87 (249.81, 273.22) | 12.36% (10.28, 14.60) | 1675 (1167, 2283) | 34.01 (23.74, 46.08) | 4638 (3299, 6307) | 38.19 (27.08, 52.55) | 12.29% (5.22, 19.30) |
| Republic of Zimbabwe | 16429 (14687, 18345) | 189.74 (170.45, 209.77) | 24157 (21694, 26915) | 174.76 (157.35, 192.97) | -7.90% (-10.28, -5.29) | 18991 (18211, 19910) | 310.66 (297.56, 325.05) | 29536 (28213, 30885) | 268.92 (257.05, 281.16) | -13.43% (-15.27, -11.67) | 2846 (1994, 3867) | 45.95 (32.05, 62.46) | 4413 (3033, 6013) | 39.61 (27.48, 53.85) | -13.80% (-18.82, -8.08) |
| Romania | 153471 (131600, 176536) | 660.07 (565.23, 763.11) | 91887 (78835, 105415) | 480.74 (410.11, 559.22) | -27.17% (-29.58, -24.49) | 275697 (264508, 288414) | 1055.53 (1012.88, 1104.50) | 207813 (198279, 217376) | 782.52 (747.41, 821.45) | -25.86% (-27.25, -24.35) | 40305 (28404, 54612) | 154.74 (109.01, 210.35) | 30200 (21101, 41084) | 115.13 (79.94, 157.62) | -25.60% (-28.62, -22.58) |
| Russian Federation | 1107179 (966670, 1257956) | 723.93 (632.18, 825.30) | 787800 (682139, 899696) | 525.79 (456.68, 604.46) | -27.37% (-30.35, -24.22) | 2075856 (1978470, 2182747) | 1215.72 (1159.00, 1277.08) | 1745451 (1662287, 1836037) | 898.70 (857.20, 944.69) | -26.08% (-27.52, -24.72) | 304381 (214062, 415207) | 178.73 (125.79, 244.52) | 253399 (177921, 344309) | 131.68 (92.24, 179.43) | -26.32% (-27.81, -24.76) |
| Saint Kitts and Nevis | 105 (92, 121) | 258.28 (227.48, 294.26) | 149 (130, 169) | 255.34 (221.77, 295.17) | -1.14% (-5.81, 3.27) | 142 (137, 148) | 404.23 (388.82, 420.65) | 291 (280, 303) | 409.23 (393.40, 425.34) | 1.24% (-1.41, 3.71) | 21 (14, 28) | 59.74 (41.34, 80.80) | 43 (30, 58) | 60.12 (42.42, 81.20) | 0.64% (-4.80, 6.29) |
| Saint Lucia | 301 (265, 343) | 224.07 (199.10, 253.66) | 399 (352, 449) | 221.63 (194.69, 252.74) | -1.09% (-4.98, 2.90) | 394 (378, 414) | 374.75 (360.91, 393.19) | 852 (819, 892) | 385.51 (370.22, 403.60) | 2.87% (0.03, 5.31) | 59 (41, 80) | 55.46 (39.01, 75.59) | 125 (88, 169) | 56.75 (40.05, 77.27) | 2.33% (-2.68, 7.70) |
| Saint Vincent and the Grenadines | 229 (201, 262) | 209.53 (185.04, 236.93) | 264 (232, 299) | 228.17 (200.27, 258.92) | 8.90% (5.17, 12.53) | 291 (280, 303) | 344.71 (332.68, 358.58) | 507 (487, 527) | 382.63 (367.55, 397.62) | 11.00% (8.68, 13.34) | 43 (30, 59) | 50.96 (35.48, 69.55) | 74 (52, 99) | 56.06 (39.13, 75.41) | 10.01% (4.04, 16.03) |
| Slovak Republic | 37767 (31904, 43578) | 700.49 (591.34, 811.00) | 32671 (27408, 38074) | 548.17 (458.25, 642.18) | -21.74% (-24.49, -19.23) | 61008 (58326, 64080) | 1071.67 (1026.19, 1124.53) | 66412 (63011, 69836) | 865.12 (823.06, 909.66) | -19.27% (-20.67, -17.78) | 8911 (6213, 12084) | 156.78 (109.22, 212.55) | 9629 (6832, 13056) | 126.54 (89.37, 172.15) | -19.29% (-22.21, -16.65) |
| Socialist Republic of Viet Nam | 140882 (124484, 159467) | 229.69 (202.22, 259.34) | 256385 (221233, 291109) | 259.27 (221.53, 297.54) | 12.88% (6.60, 18.28) | 185111 (178110, 193773) | 376.57 (362.88, 394.10) | 439900 (421986, 458911) | 418.96 (400.62, 438.19) | 11.26% (8.79, 13.78) | 27695 (19525, 37707) | 55.89 (39.30, 75.69) | 65617 (45728, 88749) | 62.16 (43.55, 84.24) | 11.22% (5.85, 16.72) |
| Solomon Islands | 713 (631, 801) | 261.94 (231.68, 294.72) | 1566 (1350, 1787) | 269.22 (228.58, 308.87) | 2.78% (-2.52, 7.77) | 893 (854, 939) | 433.50 (413.57, 454.91) | 2152 (2050, 2260) | 438.13 (417.18, 460.03) | 1.07% (-1.20, 3.57) | 134 (93, 185) | 64.29 (44.56, 88.40) | 321 (227, 439) | 64.45 (45.25, 87.45) | 0.25% (-5.06, 5.27) |
| State of Eritrea | 35993 (22545, 56367) | 946.14 (616.62, 1459.17) | 10406 (9308, 11586) | 180.84 (162.79, 201.26) | -80.89% (-87.68, -69.48) | 24455 (14819, 38333) | 814.88 (527.68, 1222.53) | 21919 (16818, 31073) | 490.25 (374.14, 692.68) | -39.84% (-47.72, -28.21) | 3629 (2473, 5499) | 119.79 (84.23, 174.53) | 3175 (2359, 4493) | 69.99 (52.08, 99.58) | -41.57% (-50.28, -30.59) |
| State of Israel | 16661 (13519, 21112) | 328.63 (267.11, 415.41) | 25844 (20493, 32577) | 272.98 (214.96, 346.73) | -16.93% (-21.62, -11.96) | 18690 (17674, 19924) | 392.40 (371.21, 417.65) | 35075 (33319, 37404) | 336.32 (318.53, 360.37) | -14.29% (-17.21, -11.03) | 2487 (1764, 3344) | 52.24 (37.13, 70.18) | 4653 (3285, 6201) | 44.86 (31.67, 60.04) | -14.13% (-19.18, -8.43) |
| State of Kuwait | 9550 (7508, 12924) | 544.13 (431.17, 730.10) | 10886 (9360, 12553) | 232.80 (202.03, 268.47) | -57.22% (-67.53, -45.75) | 8158 (7736, 8691) | 601.74 (573.30, 635.79) | 22781 (21546, 24191) | 458.90 (436.72, 483.44) | -23.74% (-26.15, -21.25) | 1238 (852, 1688) | 89.71 (61.79, 121.17) | 3377 (2418, 4507) | 67.12 (48.05, 89.76) | -25.18% (-28.79, -20.66) |
| State of Libya | 13691 (11998, 15622) | 339.12 (296.38, 382.11) | 26244 (23191, 29376) | 413.22 (364.49, 470.32) | 21.85% (12.12, 35.41) | 19061 (18140, 20151) | 670.18 (639.32, 710.96) | 52683 (49303, 57033) | 776.61 (729.83, 837.42) | 15.88% (10.55, 21.97) | 2870 (1998, 3889) | 99.66 (68.85, 135.10) | 7783 (5566, 10232) | 113.37 (81.72, 148.64) | 13.76% (7.15, 21.85) |
| State of Qatar | 1939 (1694, 2203) | 412.71 (363.15, 461.78) | 10330 (8887, 12011) | 330.35 (285.56, 377.92) | -19.96% (-24.06, -15.56) | 2864 (2731, 3026) | 755.87 (724.66, 792.83) | 18429 (17476, 19640) | 604.41 (577.04, 637.38) | -20.04% (-22.28, -17.80) | 436 (302, 600) | 112.51 (77.34, 154.66) | 2780 (1920, 3823) | 88.92 (61.75, 121.88) | -20.97% (-25.12, -16.94) |
| Sultanate of Oman | 11299 (9870, 12759) | 628.92 (554.64, 705.68) | 18046 (15874, 20328) | 413.27 (363.42, 465.32) | -34.29% (-37.70, -30.49) | 15276 (14473, 16368) | 1128.72 (1067.78, 1199.11) | 31653 (30154, 33565) | 738.93 (707.54, 775.70) | -34.53% (-36.58, -32.70) | 2321 (1597, 3180) | 168.18 (116.22, 230.41) | 4783 (3299, 6599) | 109.61 (76.27, 149.93) | -34.83% (-37.58, -32.12) |
| Swiss Confederation | 37675 (29876, 46869) | 545.52 (428.82, 684.09) | 33181 (25522, 42572) | 365.03 (276.91, 468.18) | -33.09% (-36.65, -29.18) | 57549 (54359, 60919) | 658.19 (621.17, 699.63) | 59285 (55759, 62998) | 440.23 (412.67, 469.43) | -33.12% (-35.18, -30.94) | 7543 (5303, 10087) | 86.75 (60.93, 116.95) | 7750 (5475, 10436) | 58.25 (40.60, 79.56) | -32.86% (-36.07, -29.83) |
| Syrian Arab Republic | 27908 (24475, 32219) | 220.32 (194.07, 250.23) | 33471 (28666, 39656) | 238.90 (204.33, 281.46) | 8.43% (-0.68, 21.42) | 34282 (31550, 40004) | 406.53 (378.84, 459.62) | 98756 (73917, 133816) | 746.03 (543.73, 1030.56) | 83.51% (37.28, 142.31) | 5129 (3627, 6884) | 60.10 (42.72, 80.50) | 14348 (10822, 18847) | 108.12 (80.91, 144.67) | 79.91% (37.04, 144.40) |
| Taiwan (Province of China) | 61272 (54151, 68589) | 302.14 (268.22, 338.43) | 47004 (40715, 53392) | 162.67 (142.02, 182.02) | -46.16% (-48.06, -44.31) | 106866 (102264, 112563) | 568.29 (544.75, 598.17) | 112223 (107632, 117649) | 314.11 (301.36, 328.11) | -44.73% (-46.47, -42.90) | 16126 (11049, 22069) | 85.12 (58.53, 116.52) | 16535 (11662, 22253) | 46.72 (32.81, 63.11) | -45.11% (-48.22, -41.84) |
| Togolese Republic | 5562 (4908, 6260) | 168.56 (150.98, 187.17) | 12285 (11041, 13661) | 166.98 (149.68, 185.39) | -0.94% (-3.40, 1.51) | 5713 (5493, 5972) | 263.72 (253.52, 274.88) | 15664 (15046, 16367) | 266.23 (255.82, 277.83) | 0.95% (-0.57, 2.42) | 855 (604, 1162) | 38.84 (27.33, 53.42) | 2350 (1606, 3214) | 39.40 (27.21, 53.47) | 1.43% (-4.51, 7.21) |
| Tokelau | 3 (2, 3) | 171.46 (152.31, 191.34) | 2 (2, 2) | 155.92 (136.73, 176.51) | -9.06% (-13.28, -4.83) | 4 (4, 4) | 300.13 (285.45, 315.65) | 4 (4, 4) | 270.86 (258.30, 283.54) | -9.75% (-11.60, -7.75) | 1 (0, 1) | 44.64 (31.19, 60.71) | 1 (0, 1) | 39.87 (27.59, 54.49) | -10.68% (-15.51, -4.08) |
| Turkmenistan | 11747 (10267, 13430) | 300.34 (264.58, 338.46) | 11877 (10199, 13856) | 225.67 (193.74, 263.14) | -24.86% (-28.23, -20.61) | 13693 (13121, 14316) | 487.92 (468.96, 509.02) | 18034 (17157, 18885) | 360.42 (343.23, 376.78) | -26.13% (-27.70, -24.36) | 2051 (1417, 2824) | 72.43 (50.14, 99.65) | 2677 (1888, 3731) | 53.25 (37.61, 74.18) | -26.48% (-30.37, -22.27) |
| Tuvalu | 20 (18, 22) | 217.52 (194.42, 242.66) | 25 (23, 28) | 209.55 (187.00, 234.00) | -3.66% (-7.28, -0.12) | 30 (29, 32) | 373.28 (356.76, 393.18) | 43 (40, 45) | 371.93 (353.65, 391.16) | -0.36% (-2.62, 1.83) | 5 (3, 6) | 55.73 (38.19, 76.23) | 6 (4, 9) | 55.39 (38.56, 76.16) | -0.61% (-6.43, 5.13) |
| Ukraine | 345025 (295842, 403746) | 653.02 (558.81, 769.20) | 238078 (204580, 274477) | 545.37 (471.66, 629.58) | -16.48% (-19.37, -13.40) | 683140 (648812, 717495) | 1094.65 (1039.23, 1148.70) | 555304 (526624, 585483) | 928.81 (881.55, 977.99) | -15.15% (-16.73, -13.45) | 99827 (70051, 135857) | 160.84 (112.59, 218.92) | 80700 (56394, 110447) | 136.36 (95.46, 186.68) | -15.22% (-18.37, -12.37) |
| Union of the Comoros | 712 (629, 804) | 171.67 (153.65, 191.22) | 1086 (972, 1211) | 158.85 (142.46, 177.27) | -7.47% (-10.47, -4.93) | 791 (760, 830) | 266.68 (256.15, 278.68) | 1611 (1545, 1693) | 261.31 (250.80, 273.78) | -2.02% (-3.86, -0.17) | 118 (81, 161) | 39.22 (27.15, 53.51) | 240 (168, 324) | 38.47 (27.05, 51.92) | -1.91% (-7.38, 4.09) |
| United Arab Emirates | 7564 (6618, 8612) | 419.66 (368.66, 470.94) | 41261 (35901, 46599) | 371.31 (326.77, 418.91) | -11.52% (-14.45, -8.46) | 10753 (10190, 11459) | 766.49 (731.52, 809.81) | 86717 (82049, 92152) | 734.22 (699.46, 773.81) | -4.21% (-6.41, -1.89) | 1628 (1124, 2225) | 113.36 (78.57, 153.98) | 13033 (9127, 17767) | 108.03 (75.66, 146.35) | -4.70% (-8.97, -0.34) |
| United Kingdom of Great Britain and Northern Ireland | 169137 (136600, 212473) | 307.08 (246.21, 393.90) | 167826 (129750, 213635) | 247.29 (188.59, 315.86) | -19.47% (-23.82, -15.41) | 269637 (255976, 284184) | 383.82 (363.47, 405.53) | 296551 (280206, 315907) | 308.02 (289.47, 327.91) | -19.75% (-21.39, -18.26) | 35523 (25198, 47569) | 50.91 (36.16, 68.48) | 38764 (27259, 52096) | 40.67 (28.71, 54.84) | -20.11% (-21.89, -18.42) |
| United Mexican States | 399385 (341603, 462873) | 485.84 (421.06, 557.54) | 422688 (368682, 481238) | 325.32 (283.82, 370.56) | -33.04% (-34.63, -31.45) | 502532 (479186, 525618) | 827.69 (790.30, 868.75) | 754693 (722727, 790499) | 564.57 (540.83, 591.21) | -31.79% (-32.75, -30.72) | 74422 (51984, 101796) | 121.01 (85.15, 164.23) | 110931 (77342, 150442) | 82.78 (57.82, 112.30) | -31.60% (-32.82, -30.39) |
| United Republic of Tanzania | 37813 (33241, 42534) | 155.06 (138.35, 172.72) | 75828 (67425, 85236) | 145.53 (129.31, 162.66) | -6.15% (-8.68, -3.25) | 38176 (36474, 39796) | 229.34 (220.03, 239.27) | 89086 (85270, 93313) | 228.08 (218.46, 239.01) | -0.55% (-2.20, 1.19) | 5643 (3924, 7684) | 33.50 (23.41, 45.88) | 13299 (9204, 18241) | 33.58 (23.35, 46.01) | 0.23% (-5.96, 7.56) |
| United States of America | 956916 (789072, 1183946) | 375.08 (309.70, 465.32) | 1000627 (816377, 1207968) | 273.44 (226.39, 329.68) | -27.10% (-31.28, -22.55) | 1450257 (1385573, 1534358) | 505.72 (483.19, 534.86) | 1874037 (1778513, 1973178) | 392.99 (373.51, 412.29) | -22.29% (-24.15, -20.14) | 191464 (133766, 257302) | 67.01 (46.85, 90.19) | 242469 (171125, 322667) | 51.43 (36.14, 68.26) | -23.25% (-25.42, -20.84) |
| United States Virgin Islands | 277 (247, 312) | 266.86 (237.66, 300.40) | 226 (198, 254) | 235.30 (206.88, 267.35) | -11.82% (-14.76, -8.90) | 483 (465, 504) | 474.24 (456.26, 494.13) | 502 (484, 522) | 390.67 (375.22, 405.26) | -17.62% (-19.22, -15.92) | 72 (50, 99) | 70.66 (48.83, 96.78) | 73 (51, 98) | 57.41 (39.65, 78.64) | -18.76% (-22.89, -14.63) |
|  | | | | | | | | | | | | | | | |
