# Supplementary material for: Global burden of traumatic brain injury from 1990 to 2021 and projections to 2050: A GBD 2021–based study using interpretable machine learning
Source: Medicine (Baltimore). 2026 Jul 24;105(30):e49918. doi: 10.1097/MD.0000000000049918 (PMC13406132; doi:10.1097/MD.0000000000049918)
Supplement: Supplementary file 9 [file medi-105-e49918-s009.docx]

| **Supplementary Table S6. Historical and projected ASIR, ASPR, and YLDs rates per 100,000 population for TBI for all ages by sex, 1990-2050.** | | | | | | | | |
| --- | --- | --- | --- | --- | --- | --- | --- | --- |
| **Year** | **ASIR per 100,000 No. (95% UI)** | |  | **ASPR per 100,000 No. (95% UI)** | |  | **YLDs rate per 100,000 No. (95% UI)** | |
|  | **Male** | **Female** |  | **Male** | **Female** |  | **Male** | **Female** |
| 1990 | 437.53(437.27, 437.79) | 209.86(209.68, 210.04) |  | 736.43(736.06, 736.80) | 342.40(342.16, 342.64) |  | 106.78(106.64, 106.92) | 48.39(48.30, 48.48) |
| 1991 | 442.32(442.06, 442.58) | 224.03(223.85, 224.22) |  | 732.30(731.94, 732.66) | 342.35(342.11, 342.59) |  | 106.25(106.11, 106.38) | 48.45(48.36, 48.53) |
| 1992 | 427.20(426.95, 427.45) | 204.36(204.18, 204.53) |  | 726.89(726.53, 727.24) | 339.25(339.01, 339.48) |  | 105.44(105.31, 105.58) | 47.97(47.88, 48.06) |
| 1993 | 422.63(422.38, 422.88) | 203.88(203.71, 204.05) |  | 722.53(722.18, 722.88) | 337.65(337.42, 337.88) |  | 104.85(104.72, 104.98) | 47.76(47.67, 47.85) |
| 1994 | 427.27(427.02, 427.52) | 206.08(205.91, 206.25) |  | 719.85(719.50, 720.19) | 336.71(336.48, 336.94) |  | 104.55(104.42, 104.68) | 47.65(47.57, 47.74) |
| 1995 | 418.34(418.10, 418.59) | 201.17(201.00, 201.34) |  | 716.32(715.98, 716.67) | 335.28(335.06, 335.51) |  | 104.01(103.88, 104.13) | 47.45(47.36, 47.53) |
| 1996 | 419.58(419.34, 419.82) | 200.94(200.78, 201.11) |  | 715.17(714.83, 715.51) | 334.65(334.42, 334.87) |  | 103.87(103.74, 103.99) | 47.36(47.28, 47.44) |
| 1997 | 418.47(418.23, 418.71) | 200.45(200.28, 200.61) |  | 715.55(715.21, 715.88) | 334.44(334.22, 334.66) |  | 103.94(103.82, 104.07) | 47.36(47.28, 47.44) |
| 1998 | 421.07(420.83, 421.31) | 202.59(202.42, 202.75) |  | 716.70(716.37, 717.03) | 334.54(334.32, 334.76) |  | 104.15(104.02, 104.27) | 47.38(47.30, 47.46) |
| 1999 | 420.95(420.71, 421.19) | 203.29(203.13, 203.46) |  | 716.93(716.60, 717.25) | 334.28(334.06, 334.49) |  | 104.23(104.10, 104.35) | 47.35(47.27, 47.43) |
| 2000 | 416.58(416.35, 416.82) | 198.20(198.04, 198.36) |  | 714.79(714.46, 715.11) | 332.76(332.54, 332.97) |  | 103.93(103.81, 104.05) | 47.14(47.06, 47.22) |
| 2001 | 409.87(409.64, 410.10) | 197.63(197.47, 197.79) |  | 708.49(708.17, 708.81) | 329.91(329.70, 330.13) |  | 103.03(102.91, 103.15) | 46.74(46.66, 46.82) |
| 2002 | 402.80(402.57, 403.03) | 192.63(192.47, 192.78) |  | 698.83(698.52, 699.14) | 324.95(324.74, 325.16) |  | 101.64(101.52, 101.76) | 46.03(45.95, 46.11) |
| 2003 | 396.57(396.34, 396.79) | 190.55(190.39, 190.70) |  | 687.97(687.67, 688.28) | 319.55(319.34, 319.75) |  | 100.09(99.98, 100.21) | 45.27(45.19, 45.34) |
| 2004 | 399.52(399.29, 399.74) | 199.98(199.82, 200.14) |  | 679.15(678.84, 679.45) | 316.11(315.91, 316.31) |  | 98.86(98.74, 98.97) | 44.82(44.75, 44.90) |
| 2005 | 388.15(387.93, 388.37) | 189.28(189.13, 189.43) |  | 671.79(671.49, 672.09) | 312.35(312.15, 312.54) |  | 97.79(97.68, 97.90) | 44.26(44.19, 44.33) |
| 2006 | 384.06(383.85, 384.28) | 184.95(184.80, 185.10) |  | 667.46(667.17, 667.76) | 310.22(310.03, 310.42) |  | 97.17(97.06, 97.28) | 43.94(43.87, 44.01) |
| 2007 | 381.38(381.16, 381.59) | 184.00(183.85, 184.14) |  | 664.13(663.84, 664.42) | 308.88(308.69, 309.07) |  | 96.73(96.62, 96.84) | 43.77(43.69, 43.84) |
| 2008 | 388.07(387.86, 388.29) | 199.15(199.00, 199.30) |  | 662.35(662.06, 662.63) | 309.80(309.61, 309.99) |  | 96.54(96.43, 96.65) | 43.95(43.88, 44.02) |
| 2009 | 377.83(377.62, 378.04) | 181.95(181.80, 182.09) |  | 658.97(658.69, 659.25) | 307.54(307.36, 307.73) |  | 96.05(95.95, 96.16) | 43.60(43.53, 43.67) |
| 2010 | 380.15(379.94, 380.36) | 190.09(189.95, 190.24) |  | 655.97(655.69, 656.25) | 307.11(306.92, 307.29) |  | 95.68(95.57, 95.78) | 43.58(43.51, 43.65) |
| 2011 | 370.33(370.12, 370.53) | 179.56(179.42, 179.70) |  | 650.01(649.73, 650.28) | 303.80(303.62, 303.98) |  | 94.81(94.71, 94.92) | 43.07(43.01, 43.14) |
| 2012 | 368.14(367.94, 368.35) | 177.83(177.69, 177.97) |  | 643.02(642.75, 643.29) | 299.96(299.78, 300.14) |  | 93.83(93.73, 93.93) | 42.53(42.47, 42.60) |
| 2013 | 364.45(364.25, 364.65) | 177.32(177.18, 177.46) |  | 635.48(635.22, 635.75) | 295.90(295.73, 296.08) |  | 92.77(92.67, 92.87) | 41.96(41.89, 42.03) |
| 2014 | 363.32(363.12, 363.52) | 174.52(174.38, 174.66) |  | 629.03(628.77, 629.29) | 292.00(291.83, 292.17) |  | 91.87(91.77, 91.97) | 41.40(41.34, 41.47) |
| 2015 | 359.69(359.50, 359.89) | 174.22(174.08, 174.35) |  | 624.20(623.94, 624.46) | 289.36(289.19, 289.53) |  | 91.18(91.08, 91.28) | 41.02(40.96, 41.09) |
| 2016 | 357.05(356.86, 357.25) | 172.09(171.96, 172.22) |  | 620.22(619.96, 620.47) | 287.10(286.93, 287.27) |  | 90.60(90.50, 90.69) | 40.71(40.65, 40.77) |
| 2017 | 355.25(355.06, 355.44) | 171.59(171.46, 171.72) |  | 616.21(615.96, 616.46) | 284.94(284.78, 285.11) |  | 90.03(89.93, 90.12) | 40.39(40.33, 40.45) |
| 2018 | 350.67(350.49, 350.86) | 170.19(170.06, 170.32) |  | 613.21(612.96, 613.46) | 283.24(283.08, 283.40) |  | 89.59(89.50, 89.68) | 40.13(40.07, 40.19) |
| 2019 | 348.74(348.55, 348.92) | 169.51(169.38, 169.64) |  | 613.21(612.96, 613.45) | 282.71(282.55, 282.87) |  | 89.59(89.50, 89.68) | 40.05(39.99, 40.11) |
| 2020 | 348.37(348.18, 348.55) | 170.23(170.10, 170.35) |  | 619.33(619.08, 619.57) | 284.78(284.62, 284.94) |  | 90.41(90.32, 90.50) | 40.30(40.24, 40.36) |
| 2021 | 347.84(347.65, 348.02) | 170.19(170.07, 170.32) |  | 617.91(617.67, 618.15) | 284.71(284.55, 284.87) |  | 90.15(90.05, 90.24) | 40.25(40.19, 40.30) |
| 2022 | 344.68(335.97, 353.40) | 169.04(160.81, 177.27) |  | 609.12(599.06, 619.19) | 281.51(276.32, 286.69) |  | 89.09(87.59, 90.60) | 39.85(39.09, 40.61) |
| 2023 | 340.44(329.33, 351.56) | 166.95(155.70, 178.20) |  | 603.10(590.23, 615.97) | 278.63(271.95, 285.32) |  | 88.22(86.30, 90.15) | 39.44(38.46, 40.42) |
| 2024 | 336.15(323.06, 349.24) | 164.82(151.18, 178.46) |  | 597.06(581.88, 612.25) | 275.72(267.81, 283.63) |  | 87.35(85.07, 89.62) | 39.02(37.87, 40.18) |
| 2025 | 331.75(316.94, 346.56) | 162.66(146.99, 178.33) |  | 590.94(573.72, 608.16) | 272.77(263.78, 281.75) |  | 86.46(83.88, 89.04) | 38.60(37.29, 39.91) |
| 2026 | 327.29(310.93, 343.64) | 160.49(143.02, 177.96) |  | 584.76(565.71, 603.81) | 269.83(259.88, 279.79) |  | 85.56(82.70, 88.41) | 38.18(36.72, 39.63) |
| 2027 | 322.78(305.02, 340.55) | 158.31(139.21, 177.41) |  | 578.41(557.66, 599.16) | 266.84(256.00, 277.69) |  | 84.64(81.53, 87.75) | 37.75(36.17, 39.33) |
| 2028 | 318.26(299.20, 337.33) | 156.12(135.53, 176.71) |  | 572.05(549.74, 594.37) | 263.83(252.16, 275.51) |  | 83.71(80.37, 87.06) | 37.32(35.61, 39.02) |
| 2029 | 313.68(293.41, 333.95) | 153.92(131.95, 175.88) |  | 565.70(541.92, 589.49) | 260.79(248.35, 273.23) |  | 82.79(79.22, 86.35) | 36.88(35.07, 38.69) |
| 2030 | 309.01(287.62, 330.41) | 151.68(128.44, 174.93) |  | 559.27(534.11, 584.42) | 257.73(244.56, 270.89) |  | 81.85(78.07, 85.62) | 36.44(34.52, 38.35) |
| 2031 | 304.29(281.85, 326.74) | 149.45(125.01, 173.88) |  | 552.77(526.32, 579.22) | 254.68(240.84, 268.52) |  | 80.89(76.93, 84.86) | 36.00(33.98, 38.01) |
| 2032 | 299.55(276.11, 322.98) | 147.21(121.66, 172.76) |  | 546.13(518.45, 573.80) | 251.59(237.10, 266.08) |  | 79.93(75.78, 84.08) | 35.55(33.44, 37.66) |
| 2033 | 294.80(270.43, 319.17) | 144.99(118.38, 171.59) |  | 539.50(510.66, 568.34) | 248.50(233.40, 263.60) |  | 78.96(74.63, 83.28) | 35.11(32.91, 37.31) |
| 2034 | 290.01(264.76, 315.27) | 142.74(115.16, 170.32) |  | 532.89(502.95, 562.84) | 245.38(229.70, 261.06) |  | 77.99(73.50, 82.48) | 34.66(32.38, 36.94) |
| 2035 | 285.18(259.09, 311.26) | 140.48(111.97, 168.99) |  | 526.22(495.23, 557.20) | 242.24(226.02, 258.47) |  | 77.00(72.36, 81.65) | 34.21(31.85, 36.57) |
| 2036 | 280.32(253.44, 307.19) | 138.23(108.85, 167.60) |  | 519.47(487.50, 551.45) | 239.13(222.38, 255.88) |  | 76.01(71.22, 80.81) | 33.76(31.32, 36.20) |
| 2037 | 275.45(247.82, 303.08) | 135.98(105.79, 166.17) |  | 512.60(479.68, 545.52) | 235.97(218.72, 253.22) |  | 75.00(70.07, 79.94) | 33.30(30.80, 35.81) |
| 2038 | 270.61(242.25, 298.97) | 133.74(102.78, 164.71) |  | 505.77(471.95, 539.59) | 232.82(215.09, 250.55) |  | 74.00(68.93, 79.07) | 32.85(30.27, 35.43) |
| 2039 | 265.76(236.71, 294.81) | 131.50(99.82, 163.18) |  | 498.98(464.30, 533.65) | 229.66(211.48, 247.84) |  | 73.00(67.80, 78.20) | 32.40(29.75, 35.04) |
| 2040 | 260.89(231.18, 290.61) | 129.25(96.90, 161.61) |  | 492.13(456.65, 527.61) | 226.49(207.88, 245.10) |  | 71.99(66.67, 77.31) | 31.94(29.23, 34.64) |
| 2041 | 256.03(225.67, 286.39) | 127.02(94.03, 160.01) |  | 485.23(448.99, 521.48) | 223.34(204.32, 242.36) |  | 70.97(65.54, 76.40) | 31.48(28.72, 34.24) |
| 2042 | 251.18(220.20, 282.17) | 124.80(91.22, 158.38) |  | 478.22(441.24, 515.19) | 220.15(200.74, 239.57) |  | 69.93(64.39, 75.48) | 31.02(28.20, 33.84) |
| 2043 | 246.38(214.79, 277.97) | 122.60(88.47, 156.74) |  | 471.27(433.60, 508.94) | 216.99(197.20, 236.78) |  | 68.91(63.26, 74.56) | 30.57(27.69, 33.44) |
| 2044 | 241.60(209.41, 273.78) | 120.41(85.75, 155.06) |  | 464.38(426.04, 502.71) | 213.83(193.68, 233.98) |  | 67.89(62.15, 73.63) | 30.11(27.19, 33.03) |
| 2045 | 236.82(204.06, 269.59) | 118.21(83.08, 153.34) |  | 457.45(418.49, 496.42) | 210.67(190.18, 231.16) |  | 66.86(61.02, 72.70) | 29.65(26.68, 32.63) |
| 2046 | 232.07(198.74, 265.41) | 116.04(80.47, 151.61) |  | 450.48(410.91, 490.04) | 207.52(186.71, 228.34) |  | 65.83(59.90, 71.75) | 29.20(26.18, 32.22) |
| 2047 | 227.36(193.46, 261.26) | 113.89(77.90, 149.88) |  | 443.41(403.27, 483.55) | 204.35(183.22, 225.48) |  | 64.78(58.77, 70.79) | 28.74(25.68, 31.80) |
| 2048 | 222.71(188.25, 257.16) | 111.77(75.40, 148.14) |  | 436.43(395.74, 477.13) | 201.21(179.78, 222.64) |  | 63.75(57.66, 69.84) | 28.29(25.18, 31.39) |
| 2049 | 218.10(183.08, 253.11) | 109.66(72.93, 146.38) |  | 429.53(388.30, 470.77) | 198.09(176.36, 219.81) |  | 62.73(56.55, 68.90) | 27.84(24.69, 30.98) |
| 2050 | 213.52(177.96, 249.08) | 107.56(70.51, 144.60) |  | 422.62(380.86, 464.38) | 194.97(172.97, 216.97) |  | 61.70(55.45, 67.95) | 27.39(24.20, 30.57) |
| ASIR, age-standardized incidence rates; ASPR, age-standardized prevalence rates; YLDs, years lived with disability; TBI, traumatic brain injury; UI, uncertainty interval | | | | | | | | |
